# Targeting GPVI with glenzocimab in COVID-19 patients: Results from a randomized clinical trial

**DOI:** 10.1371/journal.pone.0302897

**Published:** 2024-06-17

**Authors:** Julien Pottecher, Francois Raffi, Martine Jandrot-Perrus, Sophie Binay, Andrea Comenducci, Violaine Desort-Henin, Déborah François, Shahin Gharakhanian, Marilyn Labart, Adeline Meilhoc, Elie Toledano, Yannick Pletan, Gilles Avenard, Victor H. Sato, the GARDEN Investigators

**Affiliations:** 1 Strasbourg University Hospital, UR3072, FHU OMICARE, FMTS, Strasbourg, France; 2 Nantes Université, CHU Nantes, INSERM, Department of Infectious Diseases, CIC 1413, Nantes, France; 3 UMR_S1148 INSERM/Paris University, Paris, France; 4 Acticor-Biotech, Paris, France; 5 Shahin Gharakhanian MD Consulting LLC, Cambridge Innovation Center, Cambridge, MA, United States of America; 6 International Research Center, Hospital Alemão Oswaldo Cruz, Sao Paulo, Brazil; Sanatorio Britanico, ARGENTINA

## Abstract

**Background:**

Glenzocimab is a novel antithrombotic agent which targets platelet glycoprotein VI (GPVI) and does not induce haemorrhage. SARS-CoV-2 triggers a prothrombotic state and lung injury whose mechanisms include coagulopathy, endothelial dysfunction, and inflammation with dysregulated platelets.

**Methods and patients:**

GARDEN was a randomised double-blind, exploratory phase II study of glenzocimab in SARS-CoV-2 respiratory failure (NCT04659109). PCR+ adults in Brazil and France (7 centres) were randomized to standard-of-care (SOC) plus glenzocimab (1000 mg/dayx3 days) or placebo, followed for 40 days. Primary efficacy endpoint was clinical progression at Day 4. All analyses concerned the intention-to-treat population.

**Results:**

Between December 2020 and August 2021, 61 patients received at least one dose (30 glenzocimab vs 32 placebo) and 58 completed the study (29 *vs* 29). Clinical progression of COVID-19 ARDS was not statistically different between glenzocimab and placebo arms (43.3% and 29.0%, respectively; *p = 0*.*245*). Decrease in the NEWS-2 category at D4 was statistically significant (*p = 0*.*0290*) in the glenzocimab arm vs placebo. No Serious Adverse Event (SAE) was deemed related to study drug; bleeding related events were reported in 6 patients (7 events) and 4 patients (4 events) in glenzocimab and placebo arms, respectively.

**Conclusions:**

Therapeutic GPVI inhibition assessment during COVID-19 was conducted in response to a Public Health emergency. Glenzocimab in coagulopathic patients under therapeutic heparin was neither associated with increased bleeding, nor SAE. Clinical impact of glenzocimab on COVID-19 ARDS was not demonstrated. A potential role for GPVI inhibition in other types of ARDS deserves further experimentation. Glenzocimab is currently studied in stroke (ACTISAVE: NCT05070260) and cardiovascular indications.

## Introduction

COVID-19 is a multisystem disease which primarily manifests itself as viral pneumonia progressing to clinically complicated Acute Respiratory Distress Syndrome (ARDS). Global morbidity and mortality estimates for COVID-19 ARDS vary, with pulmonary symptoms exerting a burden on hospital-based care systems and notably Intensive Care Units. Two major mechanisms lead to lung injury: (a) directly induced lung lesions, and (b) pathophysiological consequences of overwhelming host defences triggered locally and systemically by massive immune cell/cytokine activation. Pulmonary endothelia express angiotensin converting enzyme 2 (ACE-2) to which the virus attaches and is subsequently internalized with membrane-bound ACE-2 [1, 2]. Vasculopathy is integral to COVID-19 pathophysiology with aberrant angiogenesis, endothelial cell injury, macro- and micro thrombosis and vascular dilation, to a greater extent than in other ARDS aetiologies [3, 4]. Coagulopathy and platelet hyperactivity “hallmarks” of COVID-19, contributing to an increased thrombotic burden [5, 6]. Platelets bridge the immune system and thrombosis *via* the activation and release of haemostatic and inflammatory mediators forming a ‘hub’ between thrombosis and inflammation. Increased platelet activity triggers the release of pro-inflammatory cytokines and growth factors, fuelling inflammation, favouring lung injuries documented by clinical and radiological signs [7–10]. Increase of platelet activation markers in COVID-19 patients, is associated with a higher risk of severe disease and mortality. [11–14]. There is consensus regarding the benefits of early anticoagulation in these patients [15].

Glycoprotein VI (GPVI) is a key receptor for fibrin(ogen) and collagen expressed exclusively on platelets; allowing platelet adhesion, activation, aggregation and pro-coagulant activity [16]. GPVI is not required for haemostasis and its deficiency in human and animal models is not associated with significant haemorrhage [17, 18]. While GPVI is critical in thrombosis, its blockade is protective in different animal models and is key in the thrombo-inflammatory process associated with acute ischaemic stroke [16, 19]. Additionally, GPVI-deficient mice were protected from mortality in an acute lung fibrosis model [20], suggesting a critical role for GPVI in lung fibrosis. Glenzocimab, a novel, fully humanized antibody fragment [21], binds to the extracellular domain of GPVI. It is an efficient reversible antagonist [22]. In phase I, glenzocimab at doses rising to 2000 mg proved safe and well-tolerated, no extended bleeding-time [23]. Its inhibitory effects on collagen-induced platelet aggregation, consistent pharmacokinetic ad pharmaco-dynamic properties and safety have provided foundations for the development of glenzocimab as a novel anti-thrombotic. The limited anticipated impact on physiological haemostasis suggests that glenzocimab could be combined effectively with agents such as heparin. The ARDS burden in the overall COVID-19 critical care health emergency called for studies using novel agents that might have both individual and public health impacts. A clinical trial called GARDEN [Glenzocimab in SARS-CoV-2 Acute Respiratory DistrEss syNdrome] was thus initiated to determine whether glenzocimab added to SOC could improve COVID-19 progression to ARDS via the therapeutic inhibition of glycoprotein VI.

## Methods

### Trial design oversight

GARDEN was a phase II, randomised, double blind, multicentre, placebo-controlled, parallel group, exploratory, fixed dose, efficacy, and safety trial of glenzocimab (EudraCT: 2020-002733-15, ClinicalTrial.gov: NCT04659109) conducted December 2020 to August/3Q2021 in Brazil and France. The GARDEN study was compliant with GCP guidelines and the Declaration of Helsinki, and it was approved by National Authorities. Central Ethics Committees have approved the study in France (Comité de Protection des Personnes IDF6) and in Brazil (Conselho Nacional de Saúde). Written informed consent was obtained prior to randomization. An appointed independent Data Safety Monitoring Board (DSMB), met after inclusion of 12 and 30 patients to enable study pursual, and did not request any additional safety analyses (S2 Appendix). Information allowing patient identification was restricted to local site and under supervision of local investigator.

### Patients

PCR-positive, COVID-19-hospitalised patients were enrolled, aged 18 to 80 years with moderate but progressive pulmonary disease (uni- or bilateral ground-glass opacities, or pulmonary infiltrates on chest X-Ray/CT scan, with progression over the past 48hrs). Screening was performed within 24 hrs to enable rapid inclusion (Complete list of inclusion/exclusion criteria is provided in S1 Table). Additionally, ≥ one clinical sign(s) associated with the onset of respiratory failure (e.g. respiratory rate (RR) 24 ≤ RR < 30 or SpO_2_ ≤ 93% in ambient air or 100 < PaO2/FiO2 ≤ 200), evidence of pro-thrombotic status (D-Dimers ≥ 0.5 mg/mL) and/or Troponin T/I > 2.5 mg/L and/or signs of micro-angiopathy on a vascular enhanced chest CT-scan) were also required. Informed consent was obtained from all patients. All baseline demographic and clinical characteristics (e.g., co-morbidities and concomitant treatments) were collected. Patients were recruited between December 2020 and August 2021.

### Randomisation and treatments

Eligible patients were screened for inclusion (n = 80) and the 62 patients included were randomly assigned at a 1:1 ratio to receive SOC plus either glenzocimab (n = 30) or placebo (n = 32). Permuted block randomization, with a block size of 2, was used to create a randomization list to allocate subjects to treatment arms. A random sequence number was included in each list to indicate the order in which vials should be shipped and dispensed without unblinding. Glenzocimab and placebo treatment were both colourless liquids, with identical viscosity and identical packaging and deemed indistinguishable. Study treatment was administered in 6-hour infusions of a fixed daily 1000 mg dose over 3 consecutive days. Patient recruitment was fractionated into sequential cohorts, 3 days apart, of increasing size (2, 4 then 6 patients), each balanced between the treatment arms to determine early patient tolerance and permit gradual safety verifications.

### Efficacy outcomes

The primary efficacy outcome was preventing the progression of clinical respiratory status from moderate to severe ARDS at Day 4 when glenzocimab was added to SOC, using a composite failure endpoint comprising at least one of the following at D4: 1) RR ≥ 30/min; 2) SpO_2_ decrease, provided there was no change to the type of oxygenation/ventilation system, with ventilator failure defined as an increase in oxygenation/ventilation needs regardless of SpO_2_; 3) PaO_2_/FiO_2_ ≤100mmHg, or 4) death occurring on or before D4.

When the FiO_2_ could not be precisely determined (e.g. using conventional oxygenation devices), it was estimated using validated formulae [24]. The secondary efficacy outcomes included all-cause deaths at D40, the WHO COVID-19 scale, NEWS-2 scale, RR, hypoxemia (normal: PaO_2_/FiO_2_>300mmHg, mild: 200 mmHg< PaO_2_/FiO_2_≤300 mmHg, moderate: 100 mmHg< PaO_2_/FiO_2_≤200 mmHg, severe: PaO_2_/FiO_2_≤100 mmHg).

### Safety outcomes

Any Serious Adverse Event were reported by the investigators using an SAE form emailed within 24 hours to an independent pharmacovigilance monitoring organization (AIXIAL, Boulogne-Billancourt, France). Other safety measurements included: incidence, nature/severity of AE, SAE, SUSARs and treatment-emergent AE (TEAE), bleeding-related events (BREs), hypersensitivity reactions, changes to vital signs to clinical laboratory assessments (haematology, biochemistry, urinalysis), and ECG findings *versus* screening. Bleeding-related adverse events, thrombotic events, and hypersensitivity events were to be subject to a specific analysis.

### Concomitant medications

The principal concomitant therapies administered to patients are presented in S3 Table. These mainly were antithrombotic agents (98.4%) and systemic corticosteroids (96.8%), both medication classes reached 100% in glenzocimab group, considered as SOC for COVID-19 at the time of the study.

### Dose, concentration and relation to response

Glenzocimab is currently being developed in acute ischemic stroke at a 1000 mg single dose. The dose in the GARDEN study was 1000 mg daily administered on three consecutive days. This dose was determined on the following basis (a) the time-pattern of glenzocimab pharmacology, (b) the pathophysiology of COVID-19-related ARDS which requires prolonged treatment, and (c) Expert Opinion as well as those of Regulatory Authorities when approval was sought by the study sponsor. Anti-drug antibodies (ADA) were assessed before the inclusion and at D40, Pharmacokinetics time points were taken before the start of the infusion, 3 hours after the start and 3 hours after the end of infusion during 3 consecutive days, then at D4, and sGPVI at baseline and D4.

### Statistical analysis

GARDEN study was an exploratory study which aimed at estimating a treatment difference, hence the sample size was not justified on a formal power calculation based on a reasonable expected difference. Yet assuming a true composite endpoint failure rate 0.3–0.4 in the placebo arm *versus* 0.04–0.1 in the glenzocimab arm, a sample size of 60 patients was considered to provide a statistical power of 80% to detect this difference in rates at the 1-sided alpha level of 0.025, using an unpooled variance Z test. The principle of intention-to-treat (ITT) analysis was followed, i.e., all patients randomised and assigned to a treatment arm entered the primary analysis as the “randomised set” (RS). The patient set used for the ITT primary analysis was the “full analysis set” (FAS). The FAS excluded patients from the RS if there was a lack of treatment or data after randomisation. A “per-protocol” analysis was also performed to identify the treatment effect in fully compliant, patients were in the absence of major deviations. Under the ITT principle, patients who withdrew prematurely, were non-compliant with the study treatment or received the wrong study treatment, were included in the primary analysis within the treatment arm to which they had been assigned at randomisation (“as randomised”). The primary endpoint analysis was defined as the difference in the degree of progression between treatment arms with its corresponding asymptotic 95% Wald confidence interval (95%CI) and p-value (chi-square test). Sensitivity analyses were also performed.

The distributions of categorical and quantitative (oxygen-free days only) data were respectively compared using the Cochran-Mantel-Haenszel and Wilcoxon rank sum tests, respectively. All analyses were performed using SAS version 9·4 (SAS Institute, Cary, NC, USA) and R version 4.0.3 (R Foundation, Vienna, Austria). Details of the pre-specified analytical plan are given in the Statistical Analysis Plan.

## Results

### Patients

**Fig 1:**
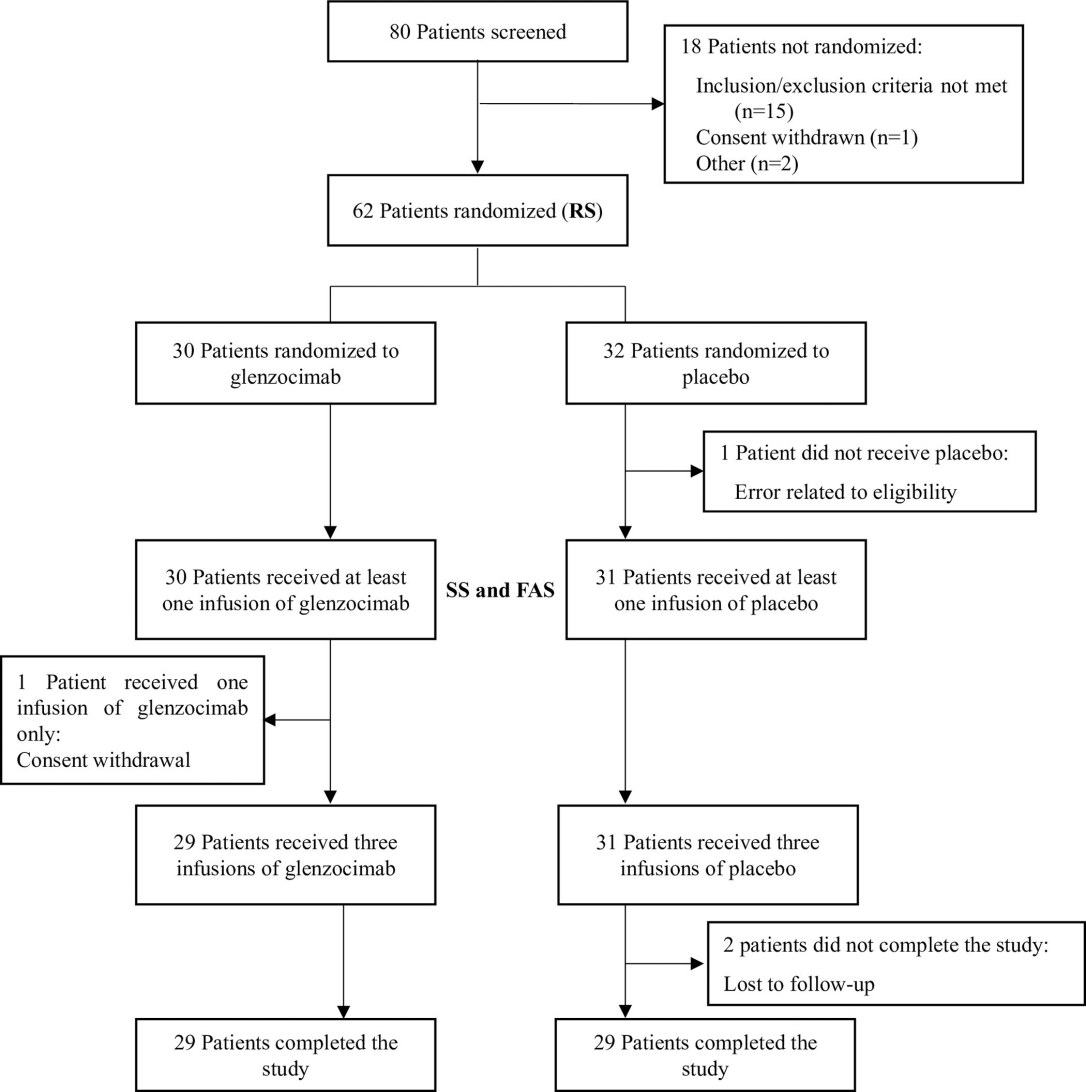
Patient disposition flow chart. RS: randomized set, used for sensitivity efficacy analyses, includes 62 patients. SS: safety set used for safety analyses, and the FAS: full analysis set, is the set for all patient having received at least a partial dose of study treatment, includes the same 61 patients.

Between December 2020 and August 2021, 80 patients were screened and 62 of them were included and followed for 40 days (91.9% Caucasian) (Fig 1). The patients were assigned randomly to glenzocimab (n = 30) or placebo (n = 32) arms; the male/female ratio was 47/15 (76%/24%) and there was a higher rate of concomitant co-morbidities in the glenzocimab arm (Table 1). The principal concomitant therapies administered to patients are presented in Table 1. These mainly consisted in antithrombotic agents (98.4%) and systemic corticosteroids (96.8%), both medication classes reached 100% in glenzocimab group, considered as SOC for COVID-19 at the time of the study.

**Table 1:** Baseline characteristics.

	Glenzocimab N = 30	Placebo N = 32	Total N = 62
**Gender**			
Female [n (%)]	9 (30.0)	6 (18.8)	15 (24.2)
Male [n (%)]	21 (70.0)	26 (81.3)	47 (75.8)
**Age (years)**			
mean (SD)	58.4 (8.1)	53.4 (11.5)	55.8 (10.2)
median	57.0	53.0	56.0
Q1 ; Q3	53.0 ; 64.0	43.5 ; 62.0	50.0 ; 63.0
**Age (categorical)**			
<60 years [n (%)]	17 (56.7)	22 (68.8)	39 (62.9)
≥60 years [n (%)]	13 (43.3)	10 (31.3)	23 (37.1)
**BMI (kg/m** ^**2**^ **)**			
mean (SD)	30.0 (4.8)	29.5 (5.9)	29.8 (5.4)
median	29.6	27.5	28.7
Q1 ; Q3	26.9 ; 33.3	25.3 ; 32.1	25.9 ; 32.3
**BMI (categorical)**			
< 30 kg/m^2^ [n (%)]	16 (53.3)	19 (61.3)	35 (57.4)
≥ 30 kg/m^2^ [n (%)]	14 (46.7)	12 (38.7)	26 (42.6)
**Race**			
American Indian or Alaska native	0 (0.0)	1 (3.1)	1 (1.6)
Asian	2 (6.7)	1 (3.1)	3 (4.8)
Black or African American	1 (3.3)	0 (0.0)	1 (1.6)
Caucasian	27 (90.0)	30 (93.8)	57 (91.9)
**Main Medical History**			
Hypertension	14 (46.7)	11 (34.4)	25 (40.3)
Lipids abnormal	11 (36.7)	9 (28.1)	20 (32.3)
Obesity	13 (43.3)	6 (18.8)	19 (30.6)
Diabetes mellitus	10 (33.3)	7 (21.9)	17 (27.4)
Hepatic steatosis	8 (26.7)	5 (15.6)	13 (21.0)
**Delay between the onset of first/earliest symptoms and** **the 1st infusion**			
mean (SD) (days)	3.6 (4.0)	4.8 (3.8)	4.2 (3.9)
**WHO COVID-19 Ordinal Scoring Scale** ^**‡**^			
Score 3 (Hospitalized, mild disease—no oxygen therapy) [n (%)]	0 (0)	1 (3.1)	1 (1.6)
Score 4 (Hospitalized, mild disease—oxygen by mask or nasal prongs) [n (%)]	21 (70.0)	20 (62.5)	(66.1)
Score 5 (Hospitalized, severe disease—Non-invasive ventilation or high-flow oxygen) [n (%)]	9 (30.0)	10 (31.3)	19 (30.6)

BMI: body mass index. Within the context of this study, patients with a “white” race, as well as patients with an “unknown” race but whose ethnicities were “Arabic”, “latino” and “mulattoes”, were classified as “Caucasian”. Main medical histort was defined as being present in more than 20% of the total population. ^**‡**^WHO COVID-19 ordinal scoring is missing for 1 patient.

### Primary efficacy outcome

**Fig 2:**
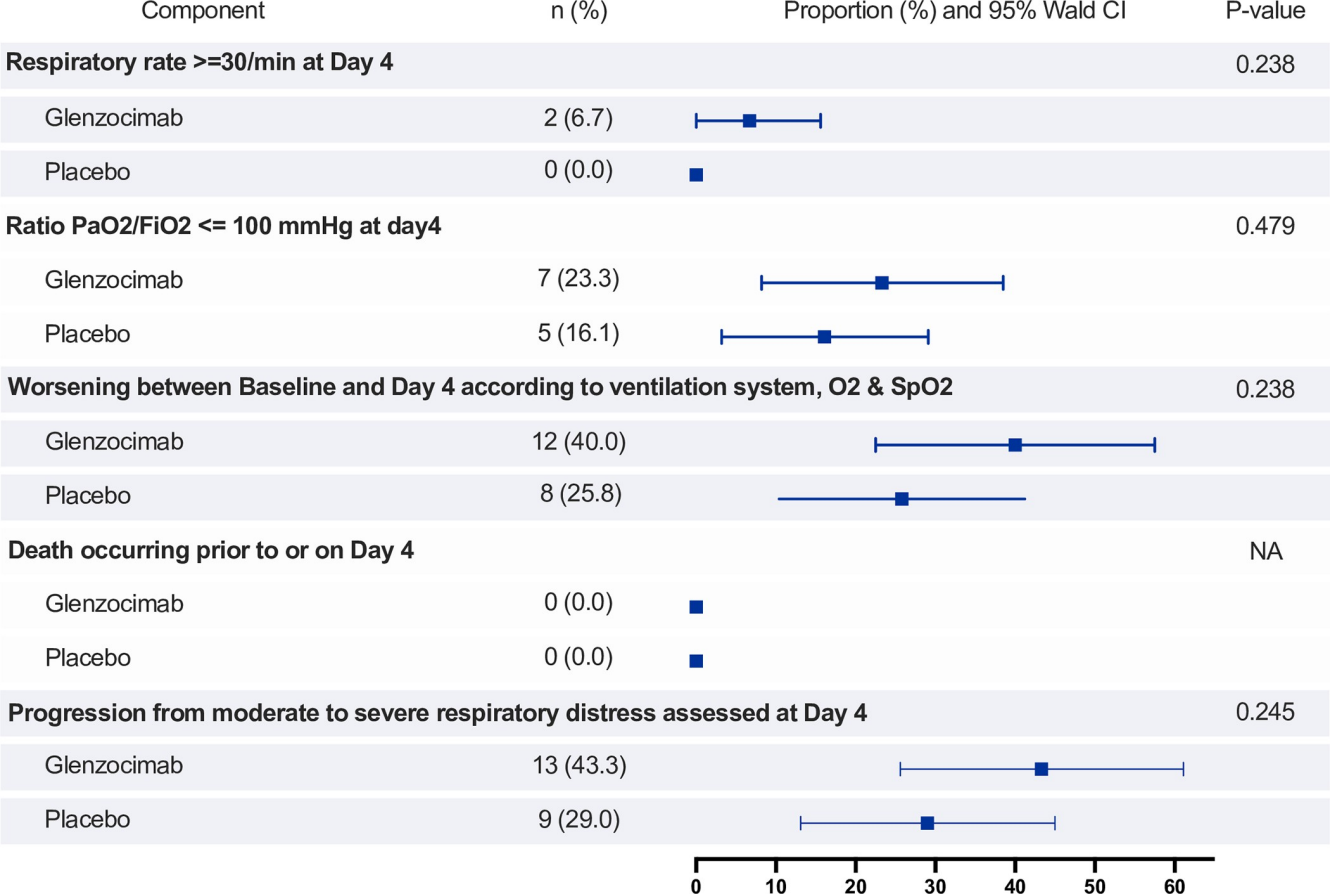
Primary Endpoint component analysis. Forrest Plot representing the Primary Efficacy Endpoint and its Components at Day 4 in the FAS (with primary method to handle missing data). Proportion of patients achieving each component and associated confidence interval (CI) and p-value.

Disease progression from moderate to severe respiratory distress at D4, as assessed using the composite failure endpoint, did not significantly differ in glenzocimab and placebo arms (43.3% and 29.0%, respectively; p = 0.245). This was confirmed in the PP analysis (S2 Table). Separate composite outcomes and subgroup analyses (by age, BMI) confirmed the absence of clinical benefit in the glenzocimab arm (S2 Table). No between-group differences in respiratory rate or the PaO_2_/FiO_2_ ratio, a worsening of escalation or the de-escalation of ventilation between baseline and D4 were observed (S2 Table and Fig 2).

### Secondary efficacy outcomes

No statistically significant between-group differences were seen in the WHO-Covid Ordinal Scoring Scale between baseline and D4, including after adjustment for age and BMI. The change in the NEWS-2 category at D4 was statistically significant (*p = 0*.*0290*) in the glenzocimab arm, with most patients recovering from the medium (baseline) to low categories (at Day 4). Placebo recipients remained in the medium category. Overall, with time, the overall number of patients in the high category decreased, with an increase in the medium category followed by the low one. Of note, there were slightly more patients from the glenzocimab group in the high category at most endpoints, including baseline. This could suggest that, initially, individuals in the glenzocimab group might have had a more severe condition compared to those in the placebo group. This statistical significance in NEWS-2 may be attributed to the differential progression of patients initially categorized as medium between the two groups. Most patients in the placebo group largely stayed within the medium category, whereas a significant number of patients in the glenzocimab group transitioned to the low category.

### Dose, concentration and relation to response

**Fig 3:**
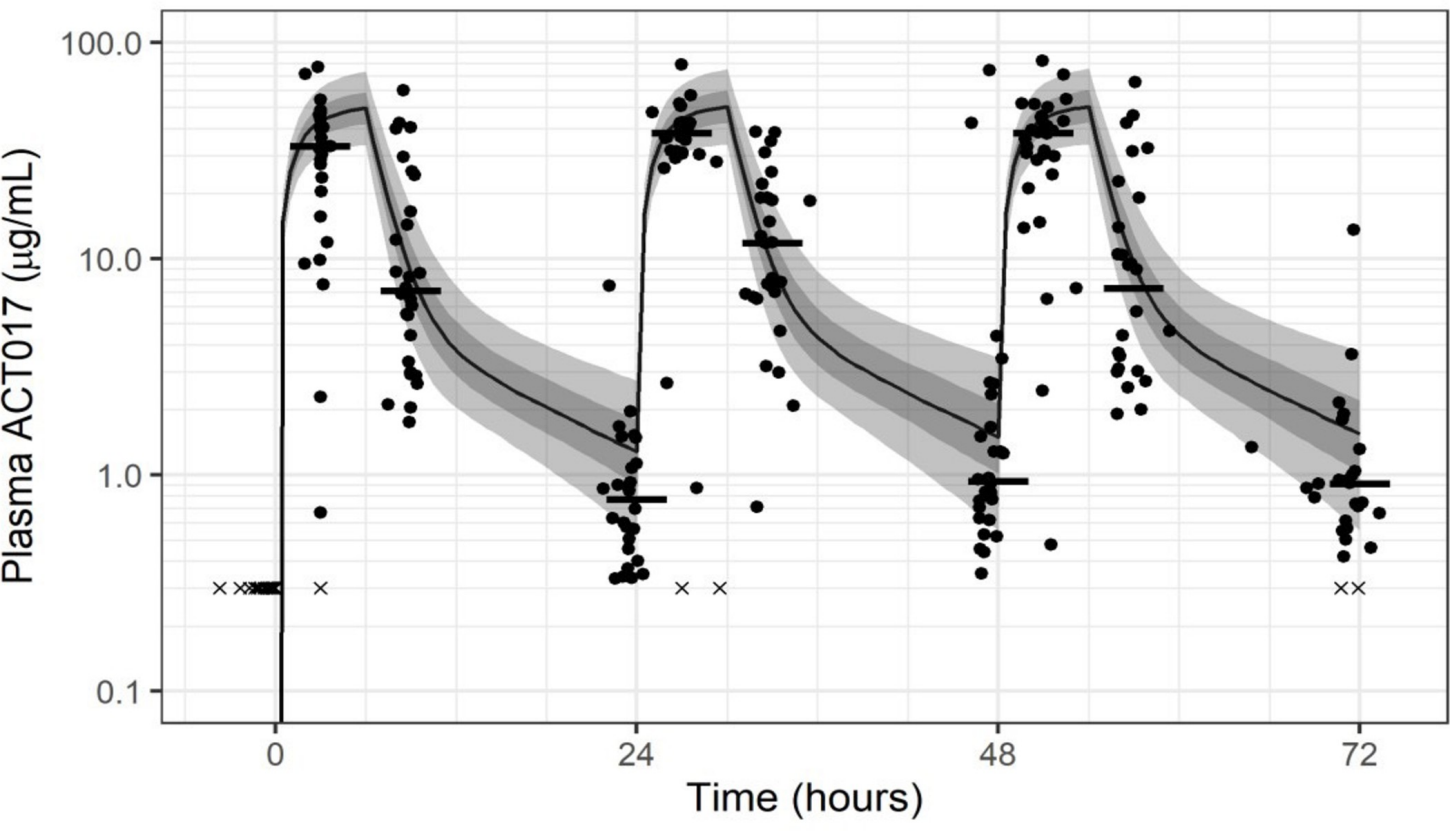
Pharmacological exposure of glenzocimab in patients. Overlay of glenzocimab concentrations from the GARDEN patients with the phase I population PK model predictions. Dots—PK measurements from GARDEN study. Crosses—samples below the limit of quantification. Horizontal lines—median of GARDEN PK measurements. Line–phase I population PK median predicted profile. Shaded area—prediction interval 5th-95th percentile. Dark shaded area - 25th-75th percentile. Note: GARDEN PK measurements plotted at actual times, median observed PK calculated with nominal times.

Glenzocimab plasma concentrations were consistent with what was expected from studies (Fig 3) and modelization in healthy volunteers, irrespective of renal function (as assessed by GFR), the repeated dosing scheme and concomitant medications. No ADA were detected at baseline or at D40. Levels of soluble GPVI in the plasma of healthy volunteers and patients from the GARDEN study were also assessed. Soluble GPVI levels were measure in the plasma in 112 samples, ranging from 1.08 ng/mL to 4.75 ng/mL. Two patients presented with elevated soluble GPVI levels at D1 and D4, higher than 5ng/mL.

### Safety outcomes

No patients died, no haemorrhagic signals, no new fact and SUSAR occurred during this study. A total of 31 SAEs (Table 2) occurred in 15 patients (24.6%), 20 on glenzocimab (9 patients; 30.0%) and 11 on placebo (6 patients; 19.4%). None of the SAEs was treatment-related. However more SAEs were observed in the glenzocimab arm than the placebo arm, which might have reflected the higher rate of concomitant co-morbidities in the glenzocimab arm. Bleeding Related Events (BREs) were considered as being of special interest. Thirteen BREs occurred in 10 patients: 7 events in 6 patients in the glenzocimab arm and 4 events in 4 patients in the placebo arm (Table 3). A single bleeding-related event (petechiae) was considered as related to glenzocimab; none were related to placebo (Table 3).

**Table 2:** Incidence and nature of SAEs per MedDRA system organ class and preferred term and per group in the safety set.

	Glenzocimab N = 30			Placebo N = 31			Total N = 61		
MedDRA System Organ Class / Preferred Term	N. AE	N. pat	% pat	N. AE	N. pat	% pat	N. AE	N. pat	% pat
Any Serious Adverse Events	20	9	30.0	11	6	19.4	31	15	24.6
**- Acute respiratory failure**	1	1	3.3	1	1	3.2	2	2	3.3
**- Pulmonary embolism**	1	1	3.3	1	1	3.2	2	2	3.3
**- Respiratory failure**	7	6	20.0	4	4	12.9	11	10	16.4
**- *Burkholderia cepacia* complex sepsis**	1	1	3.3	.	.	.	1	1	1.6
**- Sepsis**	1	1	3.3	.	.	.	1	1	1.6
**- Septic shock**	2	2	6.7	2	2	6.5	4	4	6.6
**- Staphylococcal sepsis**	1	1	3.3	.	.	.	1	1	1.6
**- Acute kidney injury**	1	1	3.3	1	1	3.2	2	2	3.3
**- Renal failure**	1	1	3.3	.	.	.	1	1	1.6
**- Disseminated intravascular coagulation**	2	2	6.7	.	.	.	2	2	3.3
**- Acute coronary syndrome**	1	1	3.3	.	.	.	1	1	1.6
**- Allergy to vaccine**	1	1	3.3	.	.	.	1	1	1.6
**- Haemoglobin decreased**	.	.	.	1	1	3.2	1	1	1.6
**- Ischaemic stroke**	.	.	.	1	1	3.2	1	1	1.6

Data are presented as the number of adverse events (N. AE) occurring in a given number of patients (N. pat). Several patients experienced more than one SAE. The percentage of patients (% pat) is calculated on the number of patients in the corresponding arm.

**Table 3:** AESI: Adverse events of special interest per MedDRA system organ class and preferred term and per group in the safety set.

	Glenzocimab N = 30			Placebo N = 31			Total N = 61		
MedDRA System Organ Class / Preferred Term	N. AE	N. pat	% pat	N. AE	N. pat	% pat	N. AE	N. pat	% pat
**Any AEs related to IMPs**	**2**	**1**	**3.3**	**2**	**2**	**6.5**	**4**	**3**	**4.9**
**- Nausea (mild)**	.	.	.	1	1	3.2	1	1	1.6
**- Hepatitis (moderate)**	.	.	.	1	1	3.2	1	1	1.6
**- Petechiae (mild)**	1	1	3.3	.	.	.	1	1	1.6
**- Hypotension (mild)**	1	1	3.3	.	.	.	1	1	1.6
**ANY BLEEDING-RELATED EVENTS**	**7**	**6**	**20.0**	**4**	**4**	**12.9**	**11**	**10**	**16.4**
**- Conjunctival haemorrhage**	.	.	.	1	1	3.2	1	1	1.6
**- Buccal haemorrhage**	1	1	3.3	.	.	.	1	1	1.6
**- Injection site hematoma**	1	1	3.3	.	.	.	1	1	1.6
**- Red blood cells urine**	1	1	3.3	1	1	3.2	2	2	3.3
**- Petechiae**	1	1	3.3	.	.	.	1	1	1.6
**- Epistaxis**	1	1	3.3	2	2	6.5	3	3	4.9
**- Hematoma**	2	2	6.7	.	.	.	2	2	3.3

Data are presented as the number of adverse events (N. AE) occurring in a given number of patients (N. pat). Several patients experienced more than one SAE. The percentage of patients (% pat) is calculated on the number of patients in the corresponding arm.

## Discussion

The key sign of COVID-19 lung injury is hypoxemia, with patients regularly reported as suffering from “silent hypoxemia” i.e., a measured PaO_2_ which is disproportionately low and contrasts with subtle clinical manifestations. This COVID-19-related hypoxemia is multifactorial: (a) vascular endothelitis, (b) diffuse alveolar damage, (c) possible failure in the compensatory mechanism of physiological hypoxic pulmonary vasoconstriction, and, (d) impaired oxygen sensing [10]. The complex pathophysiology of COVID-19 lung injury involves systemic platelet activation, the interplay between platelets, coagulation and the endothelium resulting in thrombotic complications, frequently observed in the Covid setting [25–31] and also found to be associated with a hyperactive platelet phenotype, characterized by increased activation, extracellular vesicle release, and platelet–leukocyte and platelet–endothelial cell interactions [9]. It has also been hypothesized that platelets may contribute to thrombosis in the COVID-19 setting, as platelet-rich thrombi have frequently been observed in COVID-19 patients [29, 30]. Hence, we hypothesized that a novel antiplatelet strategy based on glycoprotein VI inhibition might alleviate pulmonary symptoms in severe COVID-19. The GARDEN study was therefore initiated during the global public health emergency to explore GPVI inhibition by glenzocimab in preventing COVID-19-related ARDS progression. During this period, alpha variant was the main SARS-Cov-2 variant in circulation in Brazil and France. The glenzocimab dose selected was three times higher than the one evaluated in acute ischaemic stroke (a single dose of 1000 mg) in order to account for a more prolonged SARS-CoV-2 pathophysiological process. Based on the primary efficacy endpoint, glenzocimab failed to prevent clinical progression in this study.

Secondary efficacy endpoints (overall disease control, symptomatology and clinical recovery) were consistent with the primary endpoint, except one statistically significant difference concerning the NEWS-2 category improvement on Day 4, that could have been explained by different evolution rates between arms. Targeting GPVI at this stage of COVID-19 severity appeared to have no sufficient clinical effect to prevent the progression of ARDS. Overall, the safety results showed the safety and tolerability of 1000 mg glenzocimab administered daily on 3 consecutive days in COVID-19 patients presenting with ARDS. It should be emphasised that glenzocimab was associated with a broad panel of concomitant treatments without any clinical signals indicating drug interactions. Indeed, despite the severity of the disease, and the presence of comorbidities and association with concomitant drugs, no safety signals were recorded. The 2 cases of disseminated intravascular coagulation (DIC) were considered related to COVID-19, a complication well described in critical cases [5] and bleeding events were reported at similar frequency between treatment arms and only one case of petechiae was considered to be related to glenzocimab. Importantly, pharmacokinetic data showed that the glenzocimab concentrations in treated patients were consistent with those established in healthy volunteers [21, 23, 32], irrespective of kidney function, concomitant diseases and medications. Taken together with previous PK data, these findings confirmed that glenzocimab (which is eliminated via the renal route) did not accumulate, and kidney function within the GFR range observed was not a limiting factor for drug elimination. Previous trials had assessed the potential benefits of treating COVID-19 with antiplatelet drugs.

The RECOVERY, ACTIV-4a and REMAP-CAP trials evaluated the effects of aspirin or P2Y_12_ ADP-receptor antagonists in hospitalised patients admitted with mild to severe COVID-19 [26, 33, 34]. In ACTIV-4a and REMAP-CAP, enrolment was discontinued after the pre-specified criterion for futility was met. Overall, no additional benefits were observed in these studies. The results obtained with glenzocimab and other antiplatelet therapies seem to align and indicate that adding an antiplatelet to SOC is unlikely to improve the worsening of respiratory failure, whatever primary endpoint was used. These clinical findings contrast with the platelet activation usually detected in COVID-19 patients *ex vivo* [35] (including elevated plasma sGPVI concentrations [27, 31]) and evidence of platelet exhaustion *in vitro*. In our study, the only platelet biomarker measured was sGPVI and it was slightly elevated compared to healthy subjects, consistent with findings in the literature. However, the observation that plasma sGPVI levels did not differ between glenzocimab and placebo patients is not in favour of direct GPVI involvement in SARS-CoV-2 infection. In addition, mean plasma sGPVI levels were around 3 ng/mL, or 2% of total GPVI, thus limiting the interpretation of these data. Combined with findings from the RECOVERY, ACTIV4 and REMAP-CAP trials, GARDEN adds to the evidence that antiplatelet drugs (either marketed or not) may remain inactive on non-conventional platelet activation pathways (e.g. viral proteins, immune complexes, the low affinity Fc receptor Fcγ RIIA, complement fractions or IL6 in COVID-19) [36]. Another reason for the inefficacy of antiplatelet agents in severe COVID-19 could be a too late administration when the contribution of platelets to lung injury has become irreversible. Anticoagulants might also have altered potential effects of glenzocimab, impairing the important role of tissue factor and thrombin in platelet activation.

Results from two observational studies showed that pre-hospital antiplatelet therapy was associated with significantly lower in-hospital mortality and shorter mechanical ventilation duration in COVID-19 patients, suggesting the importance of the early administration of antiplatelet agents but the clinical benefit requires confirmation by prospective trials [36, 37].

### Limitations and strengths

The main strength of the GARDEN study was its randomised design and the strict selection of patients having both indication of rapid deterioration of respiratory status and of prothrombic state. Despite these criteria, there was a rather high heterogeneity in the platelet activation status as reflected by the wide range of sGPVI levels. Limitations included firstly a rather limited number of randomised patients which precluded demonstration of a small but still clinically relevant difference and sub-groups analyses by strata of hypercoagulability severity and/or oxygen requirement. Second, the 3 days regimen was selected based on pharmacokinetics/pharmacodynamics data in healthy volunteers and on the hypothesis that repeated dosing was needed to cover the COVID-19 pathophysiologic process. Whether higher dose or prolonged administration might provide benefit could not be assessed. Finally, genotyping was not performed during the study, but the introduction of vaccination and overall reduction of ARDS in COVID-19 patients limits further exploration of glenzocimab in the ability to prevent progression towards ARDS in SARS-Cov-2 infected patients.

## Conclusion

Further studies to understand the exaggerated inflammatory response and thrombotic events caused by SARS-Cov-2 could help to decipher novel platelet activation pathways. Glenzocimab, which is being developed in cardiovascular emergencies with a focus in acute ischemic stroke, was shown to be safe when used at duration significantly longer than those used for the primary indication, in a group of very ill patients. The safety data generated by GARDEN regarding repeated exposure, and the absence of interactions with a large panel of concomitant medications (including notably therapeutic LMWH) reinforced the therapeutic applicability of glenzocimab, i.e. safe and without entailing additional haemorrhagic risks.

## Supporting information

S1 Checklist (DOC)

S1 Fig Plasma concentration of sGPVI. The concentration of sGPVI in the plasma of patients was measured at baseline and D4. The variation in plasma concentration of sGPVI between these two times is presented for the glenzocimab (left) and the placebo (right) groups respectively.
(PDF)

S1 Table Inclusion and exclusion criteria. (PDF)

S2 Table Progression from moderate to severe respiratory distress at day 4 subgroup analysis. (PDF)

S3 Table Concomitant therapies in the randomized set. Treatments were grouped using ATC4 categories. Values are only displayed for classes received by more than 5% of all patients.
(PDF)

S1 Appendix List of investigators. (PDF)

S2 Appendix GARDEN protocol. (PDF)
