# Supplementary material for: Targeting GPVI with glenzocimab in COVID-19 patients: Results from a randomized clinical trial
Source: PLoS One. 2024 Jun 17;19(6):e0302897. doi: 10.1371/journal.pone.0302897 (PMC11182546; doi:10.1371/journal.pone.0302897)
Supplement: S1 Fig — The concentration of sGPVI in the plasma of patients was measured at baseline and D4. The variation in plasma concentration of sGPVI between these two times is presented for the glenzocimab (left) and the placebo (right) groups respectively. (PDF) [file pone.0302897.s002.pdf]

SUPPLEMENTARY MATERIAL

S1 Fig. Plasma concentration of sGPVI

The concentration of sGPVI in the plasma of patients was measured at baseline and D4. The variation in plasma concentration of sGPVI between these two times is presented for the glenzocimab (left) and the placebo (right) groups respectively.

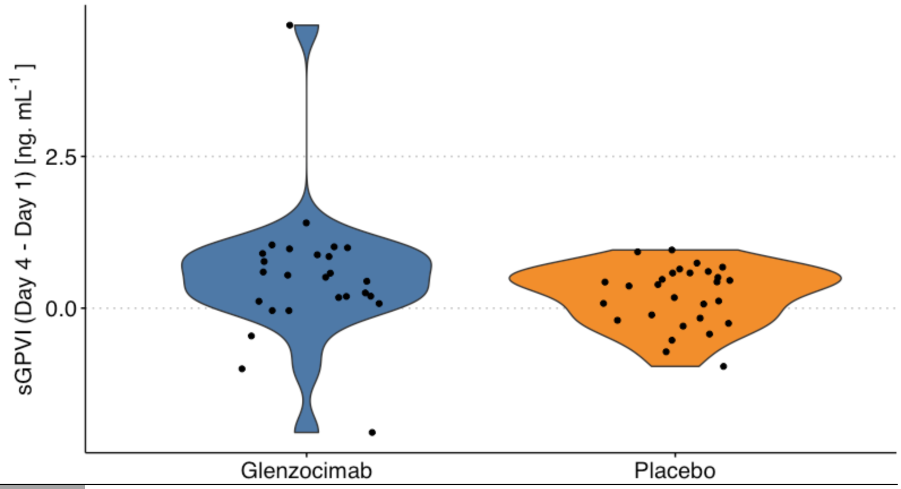

	Glenzocimab	Placebo
**n**	26	28
**Mean ± SD**	0.5 ± 1.1	0.2 ± 0.5
