# Supplementary material for: Targeting GPVI with glenzocimab in COVID-19 patients: Results from a randomized clinical trial
Source: PLoS One. 2024 Jun 17;19(6):e0302897. doi: 10.1371/journal.pone.0302897 (PMC11182546; doi:10.1371/journal.pone.0302897)
Supplement: S1 Table — (PDF) [file pone.0302897.s003.pdf]

S1 Table. Inclusion and exclusion criteria

Inclusion criteria	Exclusion criteria
1. Male or female hospitalized patients ≥ 18 years (i.e., at least 18 years old at the time of randomization) and < 80 years, having given their written consent.<br>2. Having a positive RT-PCR or antigenic test for COVID-19 or with a highly positive serology AND clear symptoms of COVID-19,<br>3. Presenting with symptoms of COVID-19, including:<br>• Cough<br>OR<br>• Shortness of breath or difficulty breathing<br>OR at least 2 of the following<br>• Fever, defined as any body temperature 38°C<br>• Chills<br>• Repeated shaking with chills<br>• Muscle pain<br>• Headache<br>• Sore throat<br>• New loss of taste or smell<br>4. Presenting with signs of moderate but progressive pulmonary disease with:<br>• respiratory symptoms (cough, dyspnea, etc.),<br>• uni- or bilateral ground-glass opacities, or pulmonary infiltrates on chest radiograph and/or CT scan performed within the past 96hrs,<br>• clinical and/or biological evidence of progression over the past 48hrs.<br><br>5. Presenting with one or several signs associated with the onset of ARDS such as:<br>• 24/min ≤ Respiratory rate (RR) < 30/min,<br>• $SpO_2 \leq 93\%$ in ambient air. In case where oxygenotherapy cannot be discontinued, please refer to NEWS 2 Scale (item SpO2, scale 2) for adequate conversion (e.g. 93-94 on O2 corresponding to 86-87 in ambient air),<br>$100 < PaO_2/FiO_2 \leq 200$mmHg (please refer to Appendix 3 for conversion).<br>6. Presenting with signs of a pro-thrombotic status characterized by<br>a. D-Dimers ≥ 0.5 µg/mL,<br>b. and/or Troponin T > 2.5 µg/L (or by default Troponin I greater than local laboratory reference),<br>c. and/or signs of micro-angiopathy on a vascular enhanced chest CT-scan.	1. Patients requiring invasive mechanical/assisted ventilation (intubation),<br>2. Obvious disseminated intravascular coagulation (DIC), (with e.g. a variable combination of the following: low platelet count (<100,000/mL), prolonged PT > 12sec and/or aPTT > 60sec, presence of fibrin degradation products in the plasma, with or without clinically visible hemorrhagic signs). An isolated change of one of these parameters does not qualify for DIC,<br>3. ARDS of another origin,<br>4. Concomitant pulmonary infection (pneumoniae) with another agent, notably bacterial or fungal,<br>5. Patients presenting with hemoglobin < 9g/dL,<br>6. Patients under immunosuppressive agents,<br>7. Patients receiving an anti-cancer treatment (radiotherapy, chemotherapy, immunotherapy),<br>8. Initiation of a treatment with aspirine (previous stable preventative aspirin regimen from 75 to 160 mg per day is allowed),<br>9. Patients under anticoagulant therapy (except heparin and low-molecular weight heparin), and anti-Xa drugs achieving effective anticoagulation, as assessed by appropriate tests, or having received thrombolytics ≤24 hrs,<br>10. Patients receiving NSAIDs or anti-platelet agents with platelet suppression within the past 7 days,<br>11. Patients treated concomitantly with another monoclonal antibody (e.g. tocilizumab)<br>12. Ischemic stroke or transient ischemic attack within the past year,<br>13. Deep venous thrombosis or pulmonary embolism within the past year,<br>14. Severe renal insufficiency (Grades 4-5) with a glomerular filtration rate <30mL/Min/1.73m^2,<br>15. One of the following severe organ failures:<br>a. Hepatic with either Child Pugh score ≥C, or ASAT/ALAT ≥5 U.N.L,<br>b. Cardiac with NYHA≥Class II, unstable angina pectoris, myocardial infarct <1

(Thrombocytopenia <150,000/mm^3 or prolonged Prothrombin Time (PT) >12s are additional signs of a pro-thrombotic status that are not necessary for eligibility).<br>7. With one or more of the following biological markers of progression:<br>• CRP ≥10 mg/L,<br>• LDH > 250 U/L,<br>• IL6 > 8 pg/mL,<br>• Lymphocyte count < 1x10^9/L,<br>• NT proBNP > 88 pg/mL,<br>• Pro-calcitonin> 0.5 ng/mL,<br>• Ferritin > 400 µg/L,<br>8. Effective birth control that should have been in place for at least 2 months in non-menopausal women and 4 months for men after IMP administration. Birth control methods considered to be highly effective include:<br>• combined (estrogen-progestogen) hormonal contraception associated with the inhibition of ovulation: oral, intravaginal, transdermal,<br>• progesterone-only hormonal contraception associated with the inhibition of ovulation: oral, injectable, implantable,<br>• intrauterine device,<br>• intrauterine hormone-releasing system,<br>• bilateral tubal occlusion,<br>• vasectomized partner.<br>9. Women of child-bearing potential must have negative results of a urinary or plasma pregnancy test (serum HCG).	year, supra-ventricular or ventricular arrhythmia,<br>16. Hereditary tendency to bleeding or coagulopathy,<br>17. Severe vascular disease (aneurysms, arterial surgery ≤6 months),<br>18. Unhealed wounds, gastrointestinal ulcers or perforation ≤6 months,<br>19. Major surgery <28 days, other surgery within the past 7 days,<br>20. Hemoptysis, GI bleeding, CNS bleeding <1 month,<br>21. Platelet count <50,000/mm3 (50G/L),<br>22. Absolute Neutrophil Count ≤1,000/mm3 (1.0G/L),<br>23. Terminal illness, including cancer (life expectancy <3 months),<br>24. Uncontrolled arterial hypertension (systolic blood pressure ≥185 mmHg and/or diastolic blood pressure ≥110 mmHg despite appropriate antihypertensive therapy,<br>25. Childbirth within <10 days,<br>26. Pregnancy or breastfeeding,<br>27. Prior cardiopulmonary resuscitation <10 days,<br>28. Allergy or hypersensitivity to drugs of the same class<br>Participation in another interventional clinical trial within 30 days prior to the inclusion.
