# Supplementary material for: Targeting GPVI with glenzocimab in COVID-19 patients: Results from a randomized clinical trial
Source: PLoS One. 2024 Jun 17;19(6):e0302897. doi: 10.1371/journal.pone.0302897 (PMC11182546; doi:10.1371/journal.pone.0302897)
Supplement: S2 Table — (PDF) [file pone.0302897.s004.pdf]

**S2 Table.** Progression from Moderate to Severe Respiratory Distress at Day 4 Subgroup analysis

| Number of patients (N (%)) | Glenzocimab N=30 | Placebo N=31 | Statistical test |
|---|---|---|---|
| **Full Analysis set** | | | |
| Yes | 13 (43.3) | 9 (29.0) | Chi-square test: p=0.245 |
| No | 17 (56.7) | 22 (71.0) | |
| **Per-protocol set** | | | |
| Yes | 12 (41.4) | 8 (28.6) | Chi-square test: p=0.311 |
| No | 17 (58.6) | 20 (71.4) | |

*Data are presented as n (%), unless otherwise stated.*
