# Supplementary material for: Targeting GPVI with glenzocimab in COVID-19 patients: Results from a randomized clinical trial
Source: PLoS One. 2024 Jun 17;19(6):e0302897. doi: 10.1371/journal.pone.0302897 (PMC11182546; doi:10.1371/journal.pone.0302897)
Supplement: S3 Table — Treatments were grouped using ATC4 categories. Values are only displayed for classes received by more than 5% of all patients. (PDF) [file pone.0302897.s005.pdf]

S3 Table. Concomitant therapies in the randomized set. *Treatments were grouped using ATC4 categories. Values are only displayed for classes received by more than 5% of all patients.*

	Glenzocimab			Placebo			Total		
	(N=30)			(N=32)			(N=62)		
Medication Class / ATC4	No. TRT	No. pat	% pat	No. TRT	No. pat	% pat	No. TRT	No. pat	% pat
- Heparin group	66	30	100.0	54	31	96.9	120	61	98.4
- Glucocorticoids	70	30	100.0	73	30	93.8	143	60	96.8
- Other cough suppressants	17	12	40.0	11	11	34.4	28	23	37.1
- Osmotically acting laxatives	17	12	40.0	12	10	31.3	29	22	35.5
- Insulins and analogues for injection, intermediate-acting	42	14	46.7	23	7	21.9	65	21	33.9
- HMG-CoA reductase inhibitors	15	11	36.7	17	9	28.1	32	20	32.3
- Other nasal preparations	23	10	33.3	9	9	28.1	32	19	30.6
- Third-generation cephalosporin	9	8	26.7	11	10	31.3	20	18	29.0
- Sulphonamides, plain	19	8	26.7	15	6	18.8	34	14	22.6
- Combinations of penicillin, incl. Beta-lactamase inhibitors	9	6	20.0	10	7	21.9	19	13	21.0
- Adrenergic in combination with corticosteroids or other drugs, excl. anticholinergics	10	9	30.0	7	3	9.4	17	12	19.4
- Opium alkaloids and derivatives	4	4	13.3	8	8	25.0	12	12	19.4
- Electrolyte solutions	17	8	26.7	8	4	12.5	25	12	19.4
- Solutions affecting the electrolyte balance	12	7	23.3	11	5	15.6	23	12	19.4
- Angiotensin ii receptor blockers (arbs), plain	9	7	23.3	4	4	12.5	13	11	17.7
- Direct factor Xa inhibitors	8	5	16.7	6	6	18.8	14	11	17.7
- Other quaternary ammonium compounds	17	7	23.3	6	4	12.5	23	11	17.7
- Other general anaesthetics	13	6	20.0	5	4	12.5	18	10	16.1
- Biguanides	11	7	23.3	4	3	9.4	15	10	16.1
- Adrenergic and dopaminergic agents	10	6	20.0	5	4	12.5	15	10	16.1
- Benzodiazepine derivatives	14	7	23.3	8	3	9.4	22	10	16.1
- Opioid anaesthetics	15	5	16.7	5	4	12.5	20	9	14.5
- Serotonin (5ht3) antagonists	5	4	13.3	7	5	15.6	12	9	14.5
- Platelet aggregation inhibitors excl. Heparin	7	6	20.0	7	3	9.4	14	9	14.5
- Benzodiazepine related drugs	5	3	10.0	9	6	18.8	14	9	14.5
- Other hypnotics and sedatives	8	5	16.7	6	4	12.5	14	9	14.5
- Diazpines, oxazepines, thiazepines and oxepines	14	4	13.3	10	4	12.5	24	8	12.9
- Beta blocking agents, selective	7	4	13.3	7	4	12.5	14	8	12.9
- Insulins and analogues for injection, long-acting	20	3	10.0	14	5	15.6	34	8	12.9
- Dihydropyridine derivatives	9	6	20.0	2	2	6.3	11	8	12.9
- Selective beta-2-adrenoreceptor agonists	7	4	13.3	5	3	9.4	12	7	11.3
- Selective serotonin reuptake inhibitors	8	5	16.7	4	2	6.3	12	7	11.3
- Macrolides	5	5	16.7	2	2	6.3	7	7	11.3

Concomitant treatment (TRT) are classified with the Anatomical Therapeutic Chemical classification Level 4 (ATC4). Data are presented as the number of adverse events (No. AE) occurring in a given number of patients (No. pat). Several patients experienced more than one SAE. The percentage of patients (% pat) is calculated on the number of patients in the corresponding arm.
