# Supplementary material for: Targeting GPVI with glenzocimab in COVID-19 patients: Results from a randomized clinical trial
Source: PLoS One. 2024 Jun 17;19(6):e0302897. doi: 10.1371/journal.pone.0302897 (PMC11182546; doi:10.1371/journal.pone.0302897)
Supplement: S1 Appendix — (PDF) [file pone.0302897.s006.pdf]

S1 Appendix. List of investigators

The following investigators participated in the conduct of the GARDEN study and had at least one patient randomized to receive treatment at their site.

Site	Principal investigator name
CHD Vendée, La Roche sur Yon, France	Dr. Gwenhaël COLIN
CHR d'Orléans, Orléans, France	Dr. Mai-Anh NAY
Pesquisare Saúde, Santo André , Brazil	Dr. Rita PELLEGRINI
Hôpital de Hautepierre, Strasbourg, France	Pr. Julien POTTECHER
Hôtel Dieu, Nantes, France	Pr. François RAFFI
Hospital Christovao da Gama, Santo André , Brazil	Dr. Edouardo RAMACCIOTTI
Hospital Alemão Oswaldo Cruz, Sao Paulo , Brazil	Pr. Victor SATO
