# Supplementary material for: Targeting GPVI with glenzocimab in COVID-19 patients: Results from a randomized clinical trial
Source: PLoS One. 2024 Jun 17;19(6):e0302897. doi: 10.1371/journal.pone.0302897 (PMC11182546; doi:10.1371/journal.pone.0302897)

S2 appendix. GARDEN Protocol

CLINICAL TRIAL PROTOCOL

Protocol number: ACT-CS-006

GARDEN STUDY

Protocol version n° 6.0 of February, 17, 2021

A RANDOMIZED, DOUBLE BLIND, MULTICENTER, PLACEBO CONTROLLED, PARALLEL GROUP, EXPLORATORY EFFICACY AND SAFETY STUDY OF GLENZOCIMAB IN SARS-Cov-2-RELATED ACUTE RESPIRATORY DISTRESS SYNDROME

EudraCT: 2020-002733-15

Phase II

Global Stud Coordinator

[redacted]

National Coordinator Investi ator for Brazil

[redacted]

SPONSOR

***ACTICOR* BIOTECH**

[redacted]

[redacted]

Sponsor

Signature Page

Study Title: A RANDOMIZED, DOUBLE BLIND, MULTICENTER, PLACEBO CONTROLLED, PARALLEL GROUP, EXPLORATORY EFFICACY AND SAFETY STUDY OF GLENZOCIMAB IN SARS-Cov-2-RELATED ACUTE RESPIRATORY DISTRESS SYNDROME

[redacted]	**Acticor-Biotech Corporate Headquarters**	**Acticor-Biotech Mail Address**
[redacted]	[redacted]	[redacted]

Global Study Coordinator

Signature Page

Study Title: A RANDOMIZED, DOUBLE BLIND, MULTICENTER, PLACEBO CONTROLLED, PARALLEL GROUP, EXPLORATORY EFFICACY AND SAFETY STUDY OF GLENZOCIMAB IN SARS-Cov-2-RELATED ACUTE RESPIRATORY DISTRESS SYNDROME

Name & Title [redacted]

Date **Si nature**

[redacted]

Investigator Statement

Signature Page

Study Title: A RANDOMIZED, DOUBLE BLIND, MULTICENTER, PLACEBO CONTROLLED, PARALLEL GROUP, EXPLORATORY EFFICACY AND SAFETY STUDY OF GLENZOCIMAB IN SARS-Cov-2-RELATED ACUTE RESPIRATORY DISTRESS SYNDROME

By my signature below, I hereby confirm that I agree to the points below:

- To conduct the trial described in the protocol, in compliance with the Good Clinical Practices (ICH-GCP), with the standard operating procedures and with all applicable regulations
- To document the delegation of significant study-related duties and to notify the Sponsor of changes in site personnel involved in the study
- To dispense, track and retain study drug in accordance with Good Clinical Practice (GCP) and protocol
- To comply with procedures and all applicable regulations for data recording and reporting including Serious Adverse Event (SAE)
- To authorize direct access to source data for monitoring, auditing and inspection
- To retain the trial-related essential documents until the Sponsor informs these documents are no longer needed.

Furthermore, I hereby confirm that I will have and will use the availability of adequate resources personnel and facilities for conduct of this trial.

Name & Title ____________________________________

Date **Signature**

___/___/______ ____________________________________

STUDY ROLES

**STEERING COMMITTEE**	A Steering Committee comprised of the following experts with [redacted] as the Chair, will monitor the study the whole duration of the clinical trial to ensure patient safety, scientific integrity of the study, interpretation of results and publications:<br>1) [redacted]
**Data Safety Monitoring Board Members**	[redacted]
**Study Monitoring**	**ACTICOR Biotech and its representatives**

**Pharmacovigilance**	[redacted]

Synopsis

**Title**	**A RANDOMIZED, DOUBLE BLIND, MULTICENTER, PLACEBO CONTROLLED, PARALLEL GROUP, FIXED DOSE, EXPLORATORY EFFICACY AND SAFETY STUDY OF GLENZOCIMAB IN SARS-Cov-2-RELATED ACUTE RESPIRATORY DISTRESS SYNDROME**
**Study Short Title**	**Glenzocimab in SARS-Cov-2 Acute Respiratory DistrEss syNdome**
**Study Acronym**	**GARDEN Study**
**Clinical Trial Number**	ACT-CS-006
**EUDRACT Number**	2020-002733-15
**Sponsor**	ACTICOR BIOTECH<br>[redacted]
**Coordinator Investigators**	Global Stud Coordinator :<br>[redacted]<br>National Brazil Coordinator :<br>[redacted]
**Clinical Phase**	II

**ADVISORY MEETING**	Given the current public health emergency, ACTICOR-BIOTECH organized an Advisory Meeting on 23 April 2020 and consulted the following advisors on the scientific relevance of our therapeutic approach, GLENZOCIMAB, a therapeutic antibody to platelet GPVI (glycoprotein VI)] added to Standard-of-Care in Covid-19 Acute Respiratory Distress Syndrome: [redacted]
**DSMB:** **Data Safety Monitoring Board**	An independent data safety monitoring board (DSMB) will be set up, before the first patient enrollment in the study. The DSMB will includes 3 experts: 2 infectious diseases or pulmonary medicine clinicians, one clinical pharmacologist. A dedicated statistician supports the DSMB members. All of them are independent of the conduct of the study. The mission of the DSMB will be to provide an informed and fully independent recommendation to the Study Steering Committee and the Study Sponsor about the conditions for continuing, amending or stopping all or part of study, based on the analysis of patient safety data. DSMB's mission and responsibilities are those described in the Good Clinical Practice *(ICH E6-R2, 2017).* DSMB is governed by a charter.
**Pharmacovigilance**	AIXIAL, [redacted]
**Study Monitoring**	ACTICOR Biotech and its representatives
**Country/Centers**	France (up to 7 sites) and Brazil (up to 4 sites).
**Study Background**	**STUDY RATIONALE: DISEASE BACKGROUND**<br><br>In January of 2020, a new virus was grown from bronchoalveolar lavage fluid samples of patients presenting with pneumonia and respiratory failure in Wuhan province, China. This virus was found to be a

	betacoronavirus, SARS-Coronavirus, Sars-Co2, the etiological agent for coronavirus disease 2019 (Covid-19) infection rapidly developing into a pandemic. The most common initial symptoms of Covid-19 are anorexia, cough, diarrhea, fatigue, fever and myalgias. Severe illness usually begins approximately 1 week after the onset of symptoms. Dyspnea is the most common symptom of severe disease and is often accompanied by hypoxemia. A striking feature of Covid-19 is notable for the rapid progression of respiratory failure soon after the onset of dyspnea and hypoxemia and these patients pmeet the criteria for the acute respiratory distress syndrome (ARDS), defined as the acute onset of bilateral infiltrates, severe hypoxemia, and lung edema that is not fully explained by cardiac failure nor fluid overload. Covid-19 diagnosis can be established on the basis of a suggestive clinical history and the detection of SARS-CoV-2 RNA in respiratory secretions. Chest radiography shows bilateral consolidations or ground-glass opacities. The epidemiological definition of, severe Covid-19 in adults is comprised of dyspnea, a respiratory rate of > 30 breaths/minute, a blood oxygen saturation of 93%, ratio of the partial pressure of arterial oxygen to the fraction of inspired oxygen (PaO_2/FiO_2) of less than 300 mm Hg, or infiltrates in more than 50% of the lung field within 24 to 48 hours from the onset of symptoms. COVID-19), a viral respiratory illness caused by the severe acute respiratory syndrome coronavirus 2 (SARS-CoV-2), may predispose patients to thrombotic disease, both in the venous and arterial circulations, due to excessive inflammation, platelet activation, endothelial dysfunction, and stasis.<br><br>**STUDY RATIONALE: PATHOPHYSIOGICAL BACKGROUND**<br><br>**Platelets and acute lung injury:**<br>Recent studies have implicated platelets in experimental lung injury and clinical Acute Respiratory Distress Syndrome *(for a review see Middelton EA. et al. AJRCMB 2018)*. Platelets are recognized for their inflammatory role and as immune effector cells in the lungs, a reservoir of numerous soluble mediators that include pro-inflammatory cytokines and pro-fibrotic growth factors. Platelets interact with leukocytes and endothelial cells impacting on endothelial permeability and vascular integrity.<br><br>Platelet count is altered in a context of critical illness, and thrombocytopenia is associated with a poor prognosis in ARDS notably caused by H1N1 influenza infection *(Lopez-Delgado JC et al. Swiss Med Wkly 2013; 143:w13788)*. The inflamed lung is a site of platelet sequestration with fibrin thrombi in micro-vessels. Intravascular fibrin

	deposition and the formation of platelet-fibrin thrombi are key features of septic acute lung injury (ALI) in humans *(see review by Bozza FA et al. AJRCMB 2009; 40:123134)*. The observation of low plasma fibrinogen levels together with elevated D-dimers that are associated with a poor prognosis in Covid-19-infected patients is in favor of a pathogenic accumulation of fibrin and platelets in their lungs, contributing to ALI and its evolution toward lung fibrosis. Genetic deficiency and pharmacological inhibitors of platelet activation diminished histologic markers of acute lung inflammation and mortality in infected animals while the activation of protease-activated receptor 4, a key platelet receptor for thrombin in mice, worsened ALI and increased mortality, indicating that platelets contributed to lung damage in this model.<br><br>Antiplatelet therapy may therefore offer a useful adjunctive strategy to fight virus-induced ALI. Most studies in human have focused on aspirin (ASA) yet but no definitive results have been obtained so far.<br><br>**COVID-19 pandemics and pro-thrombotic status**:<br><br>SARS-Cov-2 infection is increasingly being recognized as causing both inflammatory and vascular injuries *(Wadman Science 2020)*. Typically, severe pulmonary disease in SARS-Cov-2-infected patients is associated with uncontrolled inflammation and cytokine production (‘cytokine storm’) and displays the characteristics of ARDS. Indeed, an interim analysis of two clinical trials indicated that controlling the level of IL6 receptor activation can improves the prognosis in patients with severe Covid-19 respiratory distress *(in press)*. The presence of platelet and fibrin thrombi in the lungs has been observed during the histological analysis of lungs from deceased patients *(Fox et al, MedRXiv pre-print, 2020)*. Additionally, the huge quantities of growth factors released by activated platelets may favor the development of lung fibrosis as assessed from clinical and radiological signs and CT scans *(Xu VH, J infection 2020)*. Microvascular and macrovascular thrombosis have been observed in several territories (such as the lung and skin micro-vessels, as well as in large cerebral arteries) in SARS-Cov-2-infected patients *(Tchachil J Thromb Haemost 2020, Chen J Lancet Infect dis 2020, Oxley TJ NJM 2020)*. Biological findings highlight a prothrombotic state (including high plasma fibrinogen, FVIII, and platelet counts). Importantly, elevated D-dimers, the end product of fibrin degradation by fibrinolytic enzymes, are associated with an elevation of inflammation markers and have a poor prognosis in seriously affected Covid-19 patients. In the most severe cases, signs

	of intravascular coagulation are shown, including platelet consumption and a prolonged prothrombin time (PT). As a consequence, the administration of low molecular weight heparin to prevent DIC has been recommended by several Learned Societies, including the ISTH.<br><br>**Inhibiting platelet activation in SARS-Cov-2 infection**:<br><br>The potential benefit of inhibiting platelet activation in SARS-Cov-2-infected patients has been suggested by two reports: the first was a proof-of-concept trial where dipyridamole, which inhibits platelet cAMP phosphodiesterase, improved clinical outcomes in patients with Covid-19 *(Liu X et al Acta Pharm Sin B 2020)*; the second concerned the observation that the administration of Ibrutinib, a Bruton kinase inhibitor with a potent antiplatelet effect *(Rigg Am J Cell Physiol, 2016)* to a patient with a Waldenstom disease and Covid-19 symptoms rapidly improved their oxygenation parameters *(Treon et al Blood 2020)*.<br><br>To date, the use of more conventional antiplatelet agents in Covid-19 patients has not been reported. However, it should be noted that due to the aggravating effect of non-steroid anti-inflammatory drugs in Covid-19 infections, ASA at doses >100 mg/day may constitute a risk in this case. Furthermore, ASA has limited effects on activated platelets and in particular it is not a good inhibitor of the externalization of P-selectin and leukocyte-platelet interactions. Antiplatelet agents targeting the platelet ADP receptor P2Y12 or fibrinogen receptor (integrin $\alpha IIb\beta 3$) involve too high a risk of bleeding for them to be administered in these patients.<br><br>**PLATELET GLYCOPROTEIN VI (GPVI)**<br><br>GPVI is a platelet membrane glycoprotein involved in the formation, growth and stability of thrombi. GPVI belongs to the immunoglobulin receptor family, signaling via the $FcR\gamma$ chain immune tyrosine-based activation motif (ITAM) cascade that includes Bruton tyrosine kinase (Btk). An important point is that GPVI is not strictly required for physiological hemostasis since its deficiency or pharmacological blockade does not provoke significant bleeding *(Jandrot–Perrus M. et al. Platelets 2019; 30:708; Nurden AT. Blood Reviews 2019; 38:100592)*. Further, GPVI is exclusively expressed in platelets and their precursors, megakaryocytes. GPVI is therefore generally considered to be a promising target for the treatment of thrombosis *(Mackman et al Nat Rev Drug Discover 2020 March 4)*. Besides its role

	in thrombosis, GPVI is involved in platelet-monocyte interactions *(Schulz C. et al. J Thromb Haemost. 2011;9(5):1007)* and has been reported to play a critical role in the thrombo-inflammation associated with ischemia-reperfusion *(Rayes J. et al J.Clin.Invest. 2019;129:12)*. GPVI blockade reduces the inflammatory response after cerebral reperfusion, possibly by limiting the release of IL-1β and polyphosphates and by attenuating inflammatory cell recruitment *(Nieswandt B et al. J Physiol (Lond). 2011;5894115; Nieswandt B et al. J Thromb Haemost. 2011;9(Suppl 1):92)*. Similarly, GPVI blockade causes a reduction in inflammatory cell recruitment and infarct size following myocardial ischemia-reperfusion injury *(Pachel C. et al. Arterioscler Thromb Vasc Biol. 2016;36(4):629)*. GPVI has also been identified as a player in platelet interactions with pathogens. For example, staphylococcal superantigen-like 5 induces platelet activation via a mechanism involving GPIb and GPVI *(Hu H. et al Plos One 2011)*. In terms of viruses, hepatitis C virus interacts with human platelets in vivo and native viral particles have been found to bind to the extracellular domain of GPVI *(Zahn A. et al J Gene Virol 2006)*.<br><br>**Overall, GPVI could therefore be considered as a valuable candidate target to limit the contribution of platelets to uncontrolled lung inflammation without inducing unwanted bleeding.**<br><br>**GLENZOCIMAB**<br>Glenzocimab (ACT017) is a fully humanized anti human GPVI fragment of antibody (Fab) *(Lebozec K et al. N Biotechnol 2018)*. Glenzocimab blocks GPVI-triggered platelet activation in vitro. Intravenous administration of glenzocimab is antithrombotic in animal models without inducing bleeding *(Lebozec K et al. Mabs 2017)*. In a placebo-controlled phase 1 clinical trial in 48 healthy subjects, glenzocimab administered via a 6-hr infusion at escalating doses up to 2 g proved to be safe and well tolerated, with no SAE being reported, no dose or time-related adverse event, and no bleeding-time prolongation. Its pharmacokinetic/pharmacodynamic properties have been characterized *(Voors-Pette et al. Arterioscler Thromb Vasc Biol 2019;39:956)*. Glenzocimab is currently being tested in a double-blind randomized, placebo-controled, phase 2a clinical trial (ACTIMIS NCT 038030037) in patients with a severe acute ischemic stroke on top of the standard of care with doses escalating from 125 to 1000 mg administered as a single 6-hour infusion. 37 patients have already been enrolled. No specific safety signal has been reported, while the dose of 1000mg has just been reached. A Data Safety Monitoring Board

	adjudicates each new patient cohort, and, so far, after 3 scheduled meetings, no specific warning was issued.<br><br>In ARDS that entails a rapidly progressing pathological process a different pattern of administration for glenzocimab is required to cover a minimum of 3 days.<br>PK-PD modeling based on pre-existing glenzocimab PK and PD data, shows that a constant 167 mgh^{-1} infusion rate for 6 consecutive hours allows 90% of patients to have a ≥80% inhibition of collagen-induced platelet activation for 16 to 18 hrs, a threshold that is arbitrarily admitted as being representative of a full pharmacological effect for GPVI receptor inhibition. Repeating the administration at 24hr-intervals provides the assurance that there would not be any product accumulation in the blood.
**Rationale and Relevance for Patients and the Community**	To offer an additional treatment option for SARS-CoV-2 infected patients who present with acute respiratory distress syndrome. This treatment might also prevent downstream complications due to pro-thrombotic conditions. If positive, other viral sepsis conditions could benefit from this exploratory approach.
**Current Standard Therapy**	Apart from symptomatic measures, no Covid-specific standard therapy has yet been approved. Heparin and Low Molecular Weight Heparin are now recommended as per ISTH interim guidance on recognizing and managing coagulopathy in Covid-19 (and according to GIHP/GFHT joint guidance for France) for all hospitalized patients at a risk of thrombosis. Further recommendations may arise during the course of the trial, pending the results of numerous ongoing trials.
**Number of patients**	60 evaluable patients
**Objectives**	**Primary Objective:**<br>To evaluate the effect of glenzocimab in preventing clinical progression of disease, when added to Standard-of-Care in Covid-19 patients presenting with acute respiratory distress syndrome.<br><br>**Secondary Objectives:**<br>*Efficacy:*<br>• To assess the impact of treatment on overall disease control,<br>• To assess the impact of treatment on symptomatology and biological parameters.<br>*Safety:*

	• To assess the number of the following events:<br>o deaths,<br>o serious adverse events (SAEs),<br>o suspected unexpected serious adversereactions (SUSARs),<br>o medically important events,<br>o bleeding-related events.<br>*Pharmacokinetics:*<br>• To verify that actual PK profile in patients does not differ from that in healthy volunteers, and matches with PK-PD simulation.<br><br>*Exploratory*:<br>• Evolution of pulmonary lesions on chest imaging,<br>• Evolution of biological parameters related to hemostasis, coagulation and inflammation,<br>• Determination of predictive factors for a response.
**Study design**	A randomized, double blind, multicenter, placebo-controlled, parallel group, fixed dose, phase II study. The study evaluates the efficacy and safety of glenzocimab. Patients will be screened for eligibility and all tests should have results prior to any randomization, so as to avoid screening failures to a maximum extent. The turn-around time for these tests should be comprised within 24hrs to allow for rapid inclusions if needed. Eligible patients (n=68) will be randomized in a 1:1 ratio to glenzocimab or placebo. Patient inclusions will be fractioned into sequential (3-day apart) cohorts of growing size (2, 4 then 6 patients), each balanced between glenzocimab and placebo in order to check safety in a gradual manner. A Data Safety Monitoring Board (DSMB) will meet after 12 patients will have been accrued, and again after the first 30 patients. Glenzocimab will be administered by IV infusion. The dosing regimen will be 1000mg over 6 hrs, every 24 hrs for 3 consecutive days. All patients will receive in parallel the best medical care at the discretion of the investigating center, or per local guidelines. Unless, patient's condition worsens after the first infusion and/or an untoward adverse drug reaction precludes it, the treatment should be readministered 24 hrs after the initiation of the first infusion, and likewise after the second infusion, so that a total of 3 subsequent

	infusions, that represent the expected standard treatment should be administered.<br>The allocation of each patient in any given center to an active treatment or placebo will strictly follow a central randomization scheme. Clinical supplies allocation to centers should provide the necessary material so that any eligible patient can receive the assigned treatment. An IxRS will be used to manage randomization and drug shipment. The whole process will be handled in a manner that is blinded for the treatment received to all involved study personnel. The study period will be of a maximum of 40 days per patient. Patients will be closely monitored during the first 7 days following randomization with complete evaluations being performed at 24 hrs, 48 hrs, 72 hrs, then on Days 4 (96 hrs), 5 (120 hrs), 7 (+/-1 day), 14 (+/-2 days), 20 (+/-2 days), 40 (+/-3 days). Should a patient being discharged before Day 40, distant consultations by telemedicine may be undertaken if it is not deemed desirable that the patient comes back to the institution.
**Patient Safeguards and Early Stopping Rules**	Decisions regarding patient's safety are primarily made by the principal investigator and/or under his/her responsibility. This could entail discontinuing the IV infusion, not undertaking the following infusion(s), prescribing any other treatments as warranted by patient's status or evolution, terminating patient's participation to trial. However, in this latter case, close follow-up should be undertaken, notably in the presence of unresolved SAE/SUSAR. In all case, the Sponsor should be informed urgently. A DSMB: *Data Safety Monitoring Board* will be composed of at least two clinicians with expertise in the relevant clinical field, and a clinical pharmacologist. They will be supported by a dedicated statistician. The statistician will be available to analyze the data for the DSMB (either blinded or unblinded). The DSMB will be forwarded key data on a continuous basis. Two fixed interim DSMB evaluations will take place throughout study performance (i.e. after the first 12 and then 30 patients). Additional ad-hoc DSMB meetings may be set up on a need basis, in particular if SAEs/SUSARs were to be recorded, or in case of any unforeseen significant medical event that would warrant an informed safety judgment, as per DSMB charter. The Sponsor Medical Team will set up a procedure to monitor this study on an ongoing basis and closely coordinate with the PI, Steering Committee and DSMB in all safety matters
**Expected Timelines**	FPI Dec 2020, 6-month enrollment period, LPO Jul2021

**Study Duration for Patients**	40 days: 3-day treatment period + follow-up until day 40 post first treatment initiation.
**Study Visits**	For each given patient, schedule will include:<br>• A screening assessment upon admission to ensure of diagnosis and inclusion criteria<br>• At Day 1:<br>a baseline visit followed by a randomization procedure,<br>initiation of first infusion,<br>a post-infusion evaluation 3 hrs (+/-1hr) after the end of administration.<br>• At Day 2:<br>initiation of second infusion (pending results of prior evaluation)<br>a post-infusion evaluation 3 hrs (+/-1hr) after the end of second administration.<br>• At Day 3:<br>initiation of third infusion (pending results of prior evaluation)<br>a post-infusion evaluation 3 hrs (+/-1hr) after the end of third administration.<br>• At Day 4: a follow-up evaluation at 96 hrs (+/- 3 hrs), post beginning of first administration (primary end-point recording).<br>• At Day 5: a follow-up evaluation at 120 hrs (+/-3 hrs), post beginning of first administration.<br>• In case, the administration is not repeated, post-infusion evaluation at 3hrs at Day 2 and Day 3, Day 4 (96 hrs), Day 5 (120 hrs) visits should take place anyway.<br>• A follow-up visit at Days 7 (+/-1), 14 (+/-2) and 20 (+/-2 days)<br>• A 40-day (+/-3 days) post-treatment final evaluation, (End-Of- Study visit).<br>Additional follow-up visits, or phone calls may be required on a need basis notably in case of emerging or prolonged adverse events. Remote visits utilizing specific devices can be undertaken if it is not desirable that the patient, if discharged comes back to the institution

**Target population**	A total of 60 evaluable adult patients, hospitalized and presenting with acute respiratory distress syndrome that meets the following study criteria will be enrolled in the study.
**Study population**	**<u>Inclusion Criteria</u>:**<br>1. Male or female hospitalized patients ≥ 18 years (i.e., at least 18 years old at the time of randomization) and < 80 years, having given their written consent.<br><br>2. Having a positive RT-PCR or antigenic test for COVID-19 or with a highly positive serology AND clear symptoms of COVID-19,<br><br>3. Presenting with symptoms of COVID-19, including:<br>• Cough<br>OR<br>• Shortness of breath or difficulty breathing<br>OR at least 2 of the following<br>• Fever, defined as any body temperature 38°C<br>• Chills<br>• Repeated shaking with chills<br>• Muscle pain<br>• Headache<br>• Sore throat<br>• New loss of taste or smell<br><br>4. Presenting with signs of moderate but progressive pulmonary disease with:<br>• respiratory symptoms (cough, dyspnea, etc.),<br>• uni- or bilateral ground-glass opacities, or pulmonary infiltrates on chest radiograph and/or CT scan performed within the past 96hrs,<br>• clinical and/or biological evidence of progression over the past 48hrs.<br><br>5. Presenting with one or several signs associated with the onset of ARDS such as:<br>• 24/min ≤ Respiratory rate (RR) < 30/min,<br>• SpO_2 ≤ 93% in ambient air. In case where oxygenotherapy cannot be discontinued, please refer to

	NEWS 2 Scale (item SpO2, scale 2) for adequate conversion (e.g. 93-94 on O2 corresponding to 86-87 in ambient air),<br>100 < $PaO_2/FiO_2 \leq$ 200mmHg (please refer to Appendix 3 for conversion).<br>6. Presenting with signs of a pro-thrombotic status characterized by<br>a. D-Dimers ≥ 0.5 µg/mL,<br>b. and/or Troponin T > 2.5 µg/L (or by default Troponin I greater than local laboratory reference),<br>c. and/or signs of micro-angiopathy on a vascular enhanced chest CT-scan.<br>*(Thrombocytopenia <150,000/mm^3 or prolonged Prothrombin Time (PT) >12s are additional signs of a pro-thrombotic status that are not necessary for eligibility).*<br><br>7. With one or more of the following biological markers of progression:<br>• CRP ≥10 mg/L,<br>• LDH > 250 U/L,<br>• IL6 > 8 pg/mL,<br>• Lymphocyte count < 1x10^9/L,<br>• NT proBNP > 88 pg/mL,<br>• Pro-calcitonin> 0.5 ng/mL,<br>• Ferritin > 400 µg/L,<br><br>8. Effective birth control that should have been in place for at least 2 months in non-menopausal women and 4 months for men after IMP administration. Birth control methods considered to be highly effective include:<br>• combined (estrogen-progestogen) hormonal contraception associated with the inhibition of ovulation: oral, intravaginal, transdermal,<br>• progesterone-only hormonal contraception associated with the inhibition of ovulation: oral, injectable, implantable,<br>• intrauterine device,<br>• intrauterine hormone-releasing system,

- bilateral tubal occlusion,
- vasectomized partner.

9. Women of child-bearing potential must have negative results of a urinary or plasma pregnancy test (serum HCG).

<u>Non-Inclusion Criteria</u>:

1. Patients requiring invasive mechanical/assisted ventilation (intubation),
2. Obvious disseminated intravascular coagulation (DIC), (with e.g. a variable combination of the following: low platelet count (<100,000/mL), prolonged PT > 12sec and/or aPTT > 60sec, presence of fibrin degradation products in the plasma, with or without clinically visible hemorrhagic signs). An isolated change of one of these parameters does not qualify for DIC,
3. ARDS of another origin,
4. Concomitant pulmonary infection (pneumoniae) with another agent, notably bacterial or fungal,
5. Patients presenting with hemoglobin < 9g/dL,
6. Patients under immunosuppressive agents,
7. Patients receiving an anti-cancer treatment (radiotherapy, chemotherapy, immunotherapy),
8. Initiation of a treatment with aspirine (previous stable preventative aspirin regimen from 75 to 160 mg per day is allowed),
9. Patients under anticoagulant therapy (except heparin and low-molecular weight heparin), and anti-Xa drugs achieving effective anticoagulation, as assessed by appropriate tests, or having received thrombolytics ≤24 hrs,
10. Patients receiving NSAIDs or anti-platelet agents with platelet suppression within the past 7 days,
11. Patients treated concomitantly with another monoclonal antibody (e.g. tocilizumab)
12. Ischemic stroke or transient ischemic attack within the past year,
13. Deep venous thrombosis or pulmonary embolism within the past year,

	14. Severe renal insufficiency (Grades 4-5) with a glomerular filtration rate <30mL/Min/1.73m^2,<br>15. One of the following severe organ failures:<br>a. Hepatic with either Child Pugh score ≥C, or ASAT/ALAT ≥5 U.N.L,<br>b. Cardiac with NYHA≥Class II, unstable angina pectoris, myocardial infarct <1 year, supra-ventricular or ventricular arrhythmia,<br>16. Hereditary tendency to bleeding or coagulopathy,<br>17. Severe vascular disease (aneurysms, arterial surgery ≤6 months),<br>18. Unhealed wounds, gastrointestinal ulcers or perforation ≤6 months,<br>19. Major surgery <28 days, other surgery within the past 7 days,<br>20. Hemoptysis, GI bleeding, CNS bleeding <1 month,<br>21. Platelet count <50,000/mm3 (50G/L),<br>22. Absolute Neutrophil Count ≤1,000/mm3 (1.0G/L),<br>23. Terminal illness, including cancer (life expectancy <3 months),<br>24. Uncontrolled arterial hypertension (systolic blood pressure ≥185 mmHg and/or diastolic blood pressure ≥110 mmHg despite appropriate antihypertensive therapy,<br>25. Childbirth within <10 days,<br>26. Pregnancy or breastfeeding,<br>27. Prior cardiopulmonary resuscitation <10 days,<br>28. Allergy or hypersensitivity to drugs of the same class<br>29. Participation in another interventional clinical trial within 30 days prior to the inclusion.
**Efficacy Endpoints**	**Primary efficacy endpoint: Progression from moderate to severe respiratory distress assessed at Day 4.**<br><br>The primary efficacy endpoint is a composite failure endpoint defined as the occurrence of at least one of the following failure events :<br>o RR ≥ 30/min, or<br>o SpO2 decrease > 5% in ambient air. In case where oxygenotherapy cannot be discontinued, please refer to NEWS 2 Scale (item SpO2, scale 2) for adequate conversion (e.g. 93-94 on O2 corresponding to 86-87 in ambient air),<br>o $PaO_2/FiO_2 \leq$ 100mmHg,<br>o Death occurring prior to or on Day 4

	**Secondary efficacy endpoints :**<br>• All cause Death at day 40 and Overall Survival<br>• WHO-COVID-19 Scale<br>• NEWS-2 Scale<br>• Respiratory Rate status defined as::<br>o Normal: < 20/min,<br>o Mild: 20/min ≤ RR < 24/min,<br>o Moderate: 24/min ≤ RR < 30/min,<br>o Severe: ≥ 30/min,<br>o Death.<br>• Hypoxemia status defined as::<br>o Normal: > 300 mmHg,<br>o Mild: 200 mmHg < PaO2/FiO_2 ≤ 300 mmHg,<br>o Moderate: 100 mmHg <PaO2/FIO2 ≤200mmHg,<br>o Severe: PaO2/FIO2 ≤ 100mmHg,<br>o Death.<br>• SpO2 status defined as:<br>o Normal: > 95%<br>o Mild: 93% < SpO2 ≤ 95%,<br>o Moderate: 90% < SpO2 ≤ 93%,<br>o Severe: ≤ 90%,<br>o Death.<br>• Chest CT-Scan (or in exceptional cases, chest radiogram)<br>• Oxygen-free days (over the study period = 40 days),<br>• Need for mechanical ventilation,<br>• Mechanical ventilation-free days,<br>• Hospital-free days (over study period = 40 days),<br>• Clinical recovery and Time to Clinical recovery (over study period = 40 days),<br>• Cure and Time-to-cure (over study period = 40 days).
**Safety endpoints**	• Incidence, nature and severity of Adverse Events, SAEs, SUSARs and Treatment-Emergent Adverse Events (TEAEs),<br>• Incidence of bleeding-related events,<br>• Incidence of hypersensitivity reactions,<br>• Changes to vital signs over the course of the study versus screening,

	• Change to clinical laboratory assessments (hematology, biochemistry, urinalysis) over the course of the study versus screening,<br>• ECG over the course of the study versus screening.<br><br>**Soluble GPVI**:<br>Soluble GPVI (sGPVI) levels will be measured in aliquots of frozen citrated platelet poor plasma (PPP) using a sandwich immunoassay using the MesoScale Discovery (MSD) technology.<br>The concentrations will be extrapolated from standard curves generated by serial dilutions of recombinant GPVI ectodomain.
**Investigational Medicinal Product**	Glenzocimab is formulated for IV administration as a sterile product with 20 mM sodium citrate and 130 mM sodium chloride buffer at pH of 5.0. It is supplied for clinical trial use in vials containing 50 mL of the drug product at a concentration of 10 mg/mL. Each vial contains 500 mg of Glenzocimab.<br>Two vials (2x500 mg) of glenzocimab should be administered concomitantly for eligible patients for a total daily dose of 1g and a global dose of 3g during the three days of treatment.<br>The administered study treatment will be glenzocimab or placebo, as per central randomization allocation to the study group into which the patient is included.<br>In each study arm, patients will receive either glenzocimab or its matching placebo. Glenzocimab or the matching placebo is intended to be administered as an IV infusion over 6 hours.<br>The objective is therefore to cover a period of time of 12 to 24hrs, for 3 consecutive days, a period of time assumed to be long enough to turn around the ongoing pro-thrombotic events and prevent later complications.
**Concomitant treatments**	**Non-Authorized Concomitant Treatments**:<br>Patients should not receive any other experimental therapies.<br>In addition to the aforementioned exclusion criteria, patients should not receive any of the following:<br>• Anticoagulants, except LMWH or heparin, whether oral or injectable, throughout the 3-day treatment period and during the 24 hrs following the last infusion;

	• Anti-platelet agents, whether oral or injectable, throughout the 3-day treatment period and during the 24 hrs following the last infusion;<br>• Any off-label treatment, except if officially recommended for COVID-19 treatment as standard of care (SOC).<br>**Authorized Concomitant Treatments:**<br>All other symptomatic treatments used routinely for disease-related symptoms will be allowed in all patients involved in this trial. Specific treatments for any adverse events will also be permitted.<br>The use of both antibiotics and antiviral agents will be authorized.<br>The use of treatments recently recommended for the treatment of SARS-CoV-2 infection is permitted. This includes corticosteroids. However, the use of other monoclonal antibodies, even recommended is not permitted.<br>Details of all treatments or procedures must be recorded in the eCRF.
**Sample size justification**	As this is essentially an exploratory estimation study which aim is to estimate the treatment difference in order to better design the future trials, the sample size cannot be justified on a formal power calculation based on a reasonable expected difference.<br><br>60 (sixty) patients are considered sufficient to estimate the treatment effect on the efficacy parameters.<br><br>Assuming a dropout rate of 12%, 68 patients will be enrolled in this study.
**Statistical Methods**	Complete details of the statistical analyses will be provided in the Statistical Analysis Plan (SAP), which will be finalized prior to the locking and unblinding the database.<br>In general, summary tabulations will be presented by treatment arm and will display the number of observations, mean, standard deviation, median, minimum, and maximum for continuous variables, and the number and percent per category for categorical data. The Kaplan-Meier survival curves and 25th, 50th (median), and 75th percentiles will be provided along with their 2-sided 95% confidence intervals (CIs) for time-to-event data.<br><br>The nominal one-sided alpha level of significance considered for statistical tests will be 0.025.

	As this is an estimation study, no adjustment for multiplicity will be proposed.<br><br>Methods for handling missing data will be further detailed in the SAP.<br><br>**Analysis Sets:**<br><br>• **Safety set:** All enrolled patients having received at least one dose of the prescription of study medication (either ACT017 or placebo). The Safety set will be the basis for safety analyses. Patients will be assigned to the treatment group as treated for the Safety set.<br><br>• **All Randomized Set (ITT set):** All patients who are randomized into the study and considered as randomized, i.e. with study drug assignments designated according to initial randomization, regardless of whether subjects received what was assigned. Sensitivity efficacy analyses conducted on ITT set could be considered.<br><br>• **Full Analysis Set (FAS):** All randomized patients having received at least one dose of the prescription of study drug (either glenzocimab or placebo). Patients will be assigned to the treatment group as randomized (i.e. with study drug assignments designated according to initial randomization, regardless of whether subjects received what was assigned). The FAS will be considered as the primary set for efficacy analyses.<br><br>• **Per protocol (PP) set:** All FAS patients without any major protocol deviation. Major protocol deviations will be identified during data blind review meetings (i.e.) held before unblinding and database lock.<br><br>**Efficacy Analyses**<br><br>**Primary efficacy endpoint: Progression from moderate to severe respiratory distress assessed at Day 4**<br><br>Progression from moderate to severe respiratory distress assessed at Day 4 is a composite failure endpoint defined as the occurrence of at least one of the following failure events :<br><br>- RR ≥ 30/min, or<br>- SpO2 decrease > 5% in ambient air. In case where oxygenotherapy cannot be discontinued, please refer to

	NEWS 2 Scale (item SpO2, scale 2) for adequate conversion (e.g. 93-94 on O2 corresponding to 86-87 in ambient air), PaO2/FiO2 ≤ 100mmHg,<br>- Death occurring prior to or on Day 4<br><br>The composite event rate will be estimated within each group. The difference in composite event rates and the odds ratio will be estimated along with their asymptotic and exact 95% confidence intervals. p-values from the chi-square and exact Fisher test will also be provided.<br><br>Methods for handling missing components will be detailed in the SAP.<br><br>A component-wise secondary analysis will also be provided. No adjustment for multiplicity will be used.<br><br>An additional multi-component analysis will be performed to assess the proportion of patients with one failure only, 2 failures and 3 failures among the 3 components (except death).<br><br>Sensitivity analyses will be proposed and detailed in the SAP.<br><br>Additional analyses of the primary endpoint: the primary composite endpoint will be analyzed in a logistic regression model including treatment effect and relevant covariates (e.g. baseline WHO-Covid-19 scale) that will be specified in the SAP.<br><br>Subgroup analyses:<br>If feasible (i.e. when subgroup sizes are sufficient), subgroup analyses of the primary endpoint will be performed by BMI (<30 vs ≥30), Age (<65 vs ≥65 years), by baseline WHO-Covid-19 scale, comorbidity, concomitant medications will be provided and further detailed in the SAP.<br><br>The same analysis proposed for the primary efficacy endpoint assessed at day 4 will be provided at other time points (Days 1, 2, 3, 7, 14, 20, and 40). The primary endpoint and its components will also be described via plots (e.g., stacked bar plots representing the proportion of subjects in each category (failure or not) , by treatment arm, over time).<br><br>**Secondary efficacy endpoints**<br><br>o **All-Cause Mortality at day 40 (ACM40)**

	Death rates will be estimated at day 40 within each group.<br>The difference in ACM40 rates and the odds ratio will be estimated along with their asymptotic and exact 95% confidence intervals. p-values from the chi-square and exact Fisher test will also be provided.<br><br>o **Overall survival**<br><br>Overall Survival will be analyzed with the Kaplan-Meier (KM) method and the log-rank test will be used to compare the treatment arms.<br>In addition, a Proportional Hazard Cox model will be fitted to estimate the treatment effect expressed in terms of a hazard ratio with the 95% CI and p-value.<br><br>o **WHO COVID-19 Ordinal Scoring Scale**<br><br>The WHO COVID-19 Ordinal Scoring Scale is 9 point ordinal scale (i.e. not an interval scale).<br><br>A descriptive analysis of each category (count and percentage) will be performed by treatment group at each assessment day. The distribution of outcomes on WHO COVID-19 Ordinal Scoring Scale will also be described via plots (e.g., stacked bar plots representing the proportion of subjects in each category, by treatment arm, over time).<br><br>The distribution of the 9-point ordinal scale at each post randomization time point will be compared between groups with a Cochran-Mantel Haenszel test using modified ridit scores and stratified by baseline WHO COVID-19.<br><br>In addition, an analysis will be performed on the change from baseline to each time point which will be transformed into a categorized 3-point ordinal scale as follows: worsened, unchanged, and improved.<br><br>Further analyses of the WHO COVID-19 Scale will be detailed in the SAP.<br><br>**Other secondary endpoints**<br><br>The analysis of the other secondary endpoints will be detailed in the the protocol and the SAP.

	**Safety Analyses**<br>Usual descriptive statistics will be used and detailed in the SAP to analyze the safety parameters as follows:<br>• Adverse events, SAEs and death<br>• Safety laboratory tests: hematology, coagulation, biochemistry, biological markers<br><br>**Pharmacokinetics Analyses**<br><br>Pharmacokinetics will be described using all time-points and will be represented graphically.
Independent Data Safety Monitoring Board **and safety reviews.**	Safety data will be reviewed by an independent Data Safety Monitoring Board (DSMB).<br><br>- First review: the first safety review will occur after the first 12 patients enrolled have received 3 days of infusion and have been followed for 3 days after the last infusion.<br>- Second review: the second safety review will occur after the first 30 patients enrolled have received 3 days of infusion and have been followed for 3 days after the last infusion.<br>- In addition to the scheduled meetings, **ad hoc DSMB meetings** for any safety issue can be hold on request of the Glenzocimab study team (study investigators, Acticor and PV-CRO).<br><br>Further details on safety reviews and the responsibilities of the DMSB will be provided in the DSMB Charter.

Table of Contents

SYNOPSIS 6
LIST OF ABBREVIATIONS AND DEFINITIONS 32
1. INTRODUCTION AND BACKGROUND INFORMATION 34
1.1 COVID-19 Pandemic 34
1.2 PLATELET GLYCOPROTEIN VI (GPVI) 37
2. GLENZOCIMAB (ACT017) 37
2.1 RATIONALE 38
2.2 PHARMACOLOGY 38
2.2.1 Safety Pharmacology 39
2.2.2 Toxicology 39
2.2.3 Metabolism in Animals 40
2.2.4 Preliminary Clinical Experience: FIH Healthy Subjects Relevant Results 40
2.2.5 Ongoing First-in-patient Clinical Trial: ACTIMIS Study 41
2.2.6 Rationale of the Study 41
2.2.7 Study Dose Selection 42
3. STUDY OBJECTIVES 47
3.1 PRIMARY OBJECTIVE 47
3.2 SECONDARY OBJECTIVE(S) 47
3.2.1 Efficacy: 47
3.2.2 Safety: 47
3.2.3 Pharmacokinetics 47
3.2.4 Exploratory: 47
4. STUDY DESCRIPTION 48
4.1 STUDY DESIGN 48
4.2 STUDY DURATION FOR THE PATIENT 50
4.3 PREMATURE STUDY DISCONTINUATION FOR A PATIENT 51
4.3.1 Discontinuation from Study Treatment 51
4.3.2 Discontinuation from the Study 51
4.3.3 Lost to Follow-Up 51
4.4 END OF STUDY 51
4.5 INDEPENDENT DATA SAFETY MONITORING BOARD (DSMB) AND SAFETY REVIEWS 52
4.6 OVERSIGHT COMMITTEES 53
4.6.1 Mission of the Study Steering Committee 53
5. STUDY POPULATION 53
5.1 ELIGIBILITY CRITERIA 53
5.1.1 Inclusion Criteria 53
5.1.2 Non-Inclusion Criteria 55
5.2 PATIENT IDENTIFICATION 57
5.3 SCREEN FAILURES 57
5.4 EVALUABLE PATIENTS 58

6. INVESTIGATIONAL MEDICAL PRODUCT 58
6.1 ACT017 OR MATCHING PLACEBO 58
6.1.1 Glenzocimab (ACT017) 58
6.1.2 Glenzocimab (ACT017) Matching Placebo 58
6.2 MANAGEMENT OF IMP 58
6.2.1 Shipment and Receipt 58
6.2.2 Storage Condition 59
6.2.3 Dispensing and Administration of IMP 59
6.2.4 IMP Preparation and Dispensation 59
6.2.5 Dose and Administration 59
6.3 IMP ALLOCATION 60
6.3.1 Randomization 60
6.3.2 Double-Blinding 60
6.3.3 Unblinding Procedure 60
6.4 COMPLIANCE AND TREATMENT ACCOUNTABILITY 60
6.5 MANAGEMENT OF INFUSION RELATED REACTION (IRR) AND HYPERSENTIVITY REACTIONS 61
6.6 EMERGENCY PROCEDURES REGARDING SAEs 61
6.7 CONCOMITANT MEDICATIONS 62
6.7.1 Non-Authorized Concomitant Treatments 62
6.7.2 Authorized Treatments 62
7. SCHEDULE OF ASSESSEMENTS 63
7.1 STUDY FLOWCHART 63
7.2 SCREENING 64
7.3 BASELINE / RANDOMIZATION 64
7.4 TREATMENT PERIOD: 1st INFUSION AND DAY-1 EVALUATION (3 hrs (+/-1 hr) after the end of the first infusion) 65
7.5 TREATMENT PERIOD: 2nd INFUSION and DAY-2 EVALUATION (3 hrs (+/-1 hr) after the end of the second infusion) 66
7.6 TREATMENT PERIOD: 3rd INFUSION and DAY-3 EVALUATION (3 hrs (+/-1 hr) after the end of the third infusion) 66
7.7 DAY-4 EVALUATION (96 hrs) 67
7.8 DAY-5 EVALUATION (120 hrs) 68
7.9 DAY-7 EVALUATION 68
7.10 DAY-14 EVALUATION 68
7.11 DAY-20 EVALUATION 69
7.12 DAY-40: END OF STUDY VISIT 69
7.13 ASSESSMENTS AND PROCEDURES 70
7.13.1 Efficacy Assessment 70
7.13.2 Pharmacokinetics, soluble GPVI and Immunogenicity Assessments 73
8. PRIMARY ENDPOINT 74
8.1 EFFICACY 74
8.1.1 Primary Efficacy Endpoint : Progression from moderate to severe respiratory distress assessed at Day 4 74

9. SECONDARY ENDPOINTS 74
9.1 EFFICACY 74
9.2 SAFETY 75
9.3 EXPLORATORY VARIABLES (wherever possible) 75
9.4 OTHER BIOLOGICAL 75
9.4.1 Pharmacokinetics (PK) 75
9.4.2 Immunogenicity-ADA 76
9.5 DEFINITIONS 76
9.5.1 Adverse Event (AE) 76
9.5.2 Treatment Emergent Adverse Event (TEAE) 76
9.5.3 Adverse Drug Reaction (ADR) 76
9.5.4 Unexpected Adverse Drug Reaction (ADR) 76
9.5.5 Adverse Event of Special Interest (AESI) 77
9.5.6 Laboratory Test Abnormalities 77
9.6 REPORTING OF ADVERSE EVENTS 77
9.6.1 Intensity of AE 77
9.6.2 Causality 78
9.7 SERIOUS ADVERSE EVENT 78
9.7.1 Definitions 78
9.7.2 Reporting 79
10. DATA HANDLING 80
10.1 CASE REPORT FORM (CRF) 80
10.2 DATA MANAGEMENT 81
11. STATISTICAL METHODS 81
11.1 General considerations 82
11.2 Randomization and Stratification 82
11.3 Analysis Sets 82
11.4 Sample Size Determination 82
11.5 Analyses 83
11.5.1 Efficacy Analyses 83
11.5.2 Safety Analyses 86
11.5.3 Pharmacokinetic Endpoints Analysis 87
12. QUALITY CONTROL/ MONITORING 88
13. RESPONSIBILITIES 88
13.1 SPONSOR'S RESPONSIBILITIES 88
13.2 INVESTIGATOR'S RESPONSIBILITIES 88
14. CONFIDENTIALITY AND ARCHIVING 89
14.1 PERSONAL DATA PROTECTION AND CONFIDENTIALITY 89
14.2 STUDY DOCUMENTATION & ARCHIVING 89
15. ETHICAL AND REGULATORY ASPECTS 90
15.1 PATIENT'S INFORMATION NOTE AND WRITTEN CONSENT FORM 90

15.2 ACCESS TO SOURCE DATA 90
15.3 STUDY CONDUCT 90
15.4 ETHICS COMMITTEES AND COMPETENT AUTHORITIES 90
15.5 AMENDMENTS 91
15.6 AUDIT AND INSPECTION 91

16. PUBLICATION OF DATA 91

17. BIBLIOGRAPHY 92

18. APPENDICES 93

APPENDIX 1: NEWS-2 SCALE 93

APPENDIX 2: WHO COVID-19 ORDINAL SCORING SCALE 94

APPENDIX 3 : CONVERSION TABLES FOR ESTIMATION OF PaO_2 AND FiO_2 95

APPENDIX 4: HELSINKI DECLARATION 95

APPENDIX 5: STEPS FOR HANDLING ACT-CS-006 SAES 102

List of Figures

Figure 1: PK/PD model was built on glenzocimab (ACT017) plasma concentration and ex vivo platelet aggregation data from a phase I study in healthy volunteers

Figure 2: PK/PD model was built on glenzocimab (ACT017) plasma concentration and ex vivo platelet aggregation data from a phase I study in healthy volunteers

Figure 3: 166.67 mg/h as 6-h IV infusion q1day for 3 days (total dose of 3'000 mg): Predicted plasma glenzocimab (ACT017) concentration

Figure 4: 166.67 mg/h as 6-h IV infusion q1day for 3 days (total dose of 3'000 mg): Predicted inhibition of collagen-induced platelet aggregation

Figure 5: Overall Study Conduct

List of Abbreviations and Definitions

Abbreviation	Definition
ACM40	All Cause Mortality at day 40
ADA	Anti-glenzocimab antibodies
AE	Adverse Event
ADR	Adverse Drug Reaction
aPTT	activated Partial Thromboplastin Time
ARDS	acute respiratory distress syndrome
Clt	Total Clearance
CONSORT	Consolidated Standards of Reporting Trials
CRA	Clinical Research Associate
CRF/eCRF	Case Report Form/ electronic Case Report Form
CRO	Contract Research Organization
cPRP	concentrated platelet rich plasma
GCP	Good Clinical Practice
GFR	Glomerular Filtration Rate
DSMB	Independent Data Safety Monitoring Board
Fab	Fragment of Monoclonal Antibody
FIH	First In Human
FIP	First In Patient
GCP	Good Clinical Practice
GPVI	Platelet Glycoprotein VI
IEC/ IRB	Independent Ethics Committee / Independent Review Board
IMP	Investigational Medicinal Product
INR	International Normalized Ratio
MED	Minimum Effective Dose
MTD	Maximum Tolerated Dose
NCI-CTCAE	National Cancer Institute-Common Terminology Criteria for Adverse Events
NOAEL	No Observed Adverse Effect Level
PD	PharmacoDynamics
PK	PharmacoKinetics
PPS	Per Protocol Set
PT	Preferred Term
SAE	Serious Adverse Event
SAP	Statistical Analysis Plan
SDV	Source Data Verification
SmPC	Summary of Product Characteristics
SOC	Standard Of Care
SS	Safety Set
SUSAR	Suspected Unexpected Serious Adverse Reaction
TCR	Tissue Cross Reactivity

TEAE	Treatment Emergent Adverse Event
WHO	World Health Organization

1. INTRODUCTION AND BACKGROUND INFORMATION

1.1 COVID-19 Pandemic

Recent observations have demonstrated that as well as their hemostatic function, platelets contribute to tissue injury. In particular, studies during the past decade have implicated platelets in experimental lung injury and clinical Acute Respiratory Distress Syndrome *(for a review see Middelton EA. et al. AJRCMB 2018)*. Indeed, platelets are recognized for their inflammatory role and as immune effector cells in the lungs. On the one hand, platelets are a reservoir of numerous soluble mediators that include pro-inflammatory cytokines and pro-fibrotic growth factors. On the other hand, platelets interact with leukocytes and endothelial cells.
The P-selectin/PSGL-1 axis has been extensively documented for its role in the interaction between platelets and polymorph nuclear cells (PMNs) and is also involved in platelet interactions with inflammatory endothelial cells.
Platelets amplify the formation by PMNs of neutrophil extracellular traps (NETs), the pathogenic role of which has been well established in so called thombo-inflammatory processes. Platelets have complex interactions with endothelial cells which has consequences regarding endothelial permeability and vascular integrity.

The platelet count is altered in a context of critical illness, and thrombocytopenia is associated with a poor prognosis in ARDS, including H1N1 influenza infection *(Lopez-Delgado JC et al. Swiss Med Wkly 2013; 143:w13788)*. Platelet-derived TLT-1, a marker of platelet activation, is also a poor prognostic indicator in ALI/ARDS *(Morales-Ortiz J. Blood 2018; 132:2495)*. The inflamed lung is a site of platelet sequestration with fibrin thrombi in micro-vessels. Intravascular fibrin deposition and the formation of platelet-fibrin thrombi are key features of septic acute lung injury (ALI) in humans *(see review by Bozza FA et al. AJRCMB 2009; 40:123134)*. The observation of low plasma fibrinogen levels together with elevated D-dimers that are associated with a poor prognosis in Covid-19-infected patients is in favor of a pathogenic accumulation of fibrin and platelets in their lungs, contributing to ALI and its evolution toward lung fibrosis.

In the particular case of viral infection, activated platelets and platelet-monocyte aggregates have been identified in blood samples from patients with ARDS caused by influenza *A (Rondina MT et al. Chest 2012;141:1490)*. It has been proposed that activated platelets mediate aberrant hemostasis and maladaptive "hyper" inflammation by interacting with leukocytes and endothelial cells in the context of severe influenza infection *(Yang Y. et al. Cell Mol Immunol. 2016;13:432)*. The infection of lung endothelial cells by viruses, resulting in platelet adhesion, is suggested to be a mechanism for influenza-induced ALI, based on parallel studies of human and mice lung microvascular endothelial cells and mice infected with H3N2 influenza A *(Katz JN et al. Chest 2011;138 :658; Sugiyama MG et al J Virol. 2016;90/1812)*. In a mouse model of severe infection by influenza virus A, histopathologic studies demonstrated an accumulation of activated platelets in the lungs *(Lê VB et al. Am J Respir Crit Care Med 2015 ;191:804)*. During the same study, viral proteins were detected within platelets.

Serotonin and Il-1β were found in bronchoalveolar lavage specimens, with serotonin being largely stored in platelets that released it following activation, while Il-1β was being produced by activated platelets. Genetic deficiency and pharmacological inhibitors of platelet activation diminished histologic markers of acute lung inflammation and mortality in infected animals. By contrast, the activation of protease-activated receptor 4, a key platelet receptor for thrombin in mice, worsened ALI and increased mortality, indicating that platelets contributed to lung damage in this model and suggesting that antiplatelet drugs might interrupt the course of inflammatory injury in influenza pneumonia. Observations during a second study indicated that H1N1 influenza activates platelets via a complex mechanism that includes the FcγRIIA immunoreceptor and thrombin generation, thus involving both the innate and adaptive immune pathways *(Boilard E et al. Blood 2014; 123:2854)*. Antiplatelet therapy may therefore offer a useful adjunctive strategy to fight virus-induced ALI. Most studies in human have focused on aspirin (ASA) yet but no definitive results have been obtained so far.

It should also be noted that platelets may contribute to iatrogenic complications, particularly when mechanical ventilation is excessive *(Matthay MA. et al. J Clin Invest 2012;122:2731; Yadav H et al. Am J Respir Crit Care Med 2017;195:725)*.

More specifically, infection by SARS-Cov-2 is increasingly being recognized as causing both inflammatory and vascular injuries *(Wadman Science 2020)*. Typically, severe pulmonary disease in SARS-Cov-2-infected patients is associated with uncontrolled inflammation and cytokine production (‘cytokine storm’) and displays the characteristics of ARDS. Indeed, an interim analysis of two clinical trials indicated that controlling the level of IL6 receptor activation can improves the prognosis in patients with severe Covid-19 respiratory distress *(in press)*. As mentioned above, platelets have been shown to be important contributors in ARDS and the presence of platelet and fibrin thrombi in the lungs has been observed during the histological analysis of lungs from deceased patients *(Fox et al, MedRXiv pre-print, 2020)*. Additionally, the huge quantities of growth factors released by activated platelets may favor the development of lung fibrosis as assessed from clinical and radiological signs and CT scans *(Xu VH, J infection 2020)*.

Specifically, SARS-Cov-2 infects many cells and organs beyond the lung epithelium. In particular it enters blood cells. Endothelial cells have been shown to be infected by the virus, causing “endothelitis” *(Varga et al Lancet 2020)*. Inflamed endothelial cells acquire an ability to trigger the adhesion of blood cells, including platelets. Furthermore, they lose their capacity to inhibit the coagulation cascade, allowing the generation of thrombin at the surface of activated platelets and fibrin formation that ultimately recruits more platelets. Microvascular and macrovascular thrombosis have been observed in several territories (such as the lung and skin micro-vessels, as well as in large cerebral arteries) in SARS-Cov-2-infected patients *(Tchachil J Thromb Haemost 2020, Chen J Lancet Infect dis 2020, Oxley TJ NJM 2020)*. Biological findings highlight a prothrombotic state (including high plasma fibrinogen, FVIII, and platelet counts). Importantly, elevated D-dimers, the end product of fibrin degradation by fibrinolytic enzymes, are associated with an elevation of inflammation markers and have a poor prognosis in seriously affected Covid-

19 patients. In the most severe cases, signs of intravascular coagulation are shown, including platelet consumption and a prolonged prothrombin time (PT). As a consequence, the administration of low molecular weight heparin to prevent DIC has been recommended by several Learned Societies, including the ISTH. Importantly, sera from Covid-19 patients has been shown to contain elevated levels of cell-free DNA, myeloperoxidase (MPO)-DNA, and citrullinated histone H3 (Cit-H3); the latter two are highly specific markers of NETs. Cell-free DNA is strongly correlated with acute phase reactants and the neutrophil count. Cit-H3 correlates with platelet levels, thus favoring a role for platelets in the formation of NETs in this context *(Zhuo et al JCI insight 2020)*.

One potential benefit of inhibiting platelet activation in SARS-Cov-2-infected patients has been suggested by two reports: the first was a proof-of-concept trial where dipyridamole, which inhibits platelet cAMP phosphodiesterase, improved clinical outcomes in patients with Covid-19 *(Liu X et al Acta Pharm Sin B 2020)*; the second concerned the observation that the administration of Ibrutinib, a Bruton kinase inhibitor with a potent antiplatelet effect *(Rigg Am J Cell Physiol, 2016)* to a patient with a Waldenstom disease and Covid-19 symptoms rapidly improved their oxygenation parameters *(Treon et al Blood 2020)*.

To date, the use of more conventional antiplatelet agents in Covid-19 patients has not been reported. However, it should be noted that due to the aggravating effect of non-steroid anti-inflammatory drugs in Covid-19 infections, ASA at doses >100 mg/day may constitute a risk in this case. Furthermore, ASA has limited effects on activated platelets and in particular it is not a good inhibitor of the externalization of P-selectin and leukocyte-platelet interactions. Antiplatelet agents targeting the platelet ADP receptor P2Y12 or fibrinogen receptor (integrin $\alpha IIb\beta 3$) involve too high a risk of bleeding for them to be administered in these patients.

1.2 PLATELET GLYCOPROTEIN VI (GPVI)

GPVI is a platelet membrane glycoprotein involved in the formation, growth and stability of thrombi. GPVI belongs to the immunoglobulin receptor family, signaling via the FcRγ chain immune tyrosine-based activation motif (ITAM) cascade that includes Bruton tyrosine kinase (Btk). An important point is that GPVI is not strictly required for physiological hemostasis since its deficiency or pharmacological blockade does not provoke significant bleeding *(Jandrot–Perrus M. et al. Platelets 2019; 30:708; Nurden AT. Blood Reviews 2019; 38:100592)*. Further, GPVI is exclusively expressed in platelets and their precursors, megakaryocytes. GPVI is therefore generally considered to be a promising target for the treatment of thrombosis *(Mackman et al Nat Rev Drug Discover 2020 March 4)*. Besides its role in thrombosis, GPVI is involved in platelet-monocyte interactions *(Schulz C. et al. J Thromb Haemost. 2011;9(5):1007)* and has been reported to play a critical role in the thrombo-inflammation associated with ischemia-reperfusion *(Rayes J. et al J.Clin.Invest. 2019;129:12)*. GPVI blockade reduces the inflammatory response after cerebral reperfusion, possibly by limiting the release of IL-1β and polyphosphates and by attenuating inflammatory cell recruitment *(Nieswandt B et al. J Physiol (Lond). 2011;5894115; Nieswandt B et al. J Thromb Haemost. 2011;9(Suppl 1):92)*. Similarly, GPVI blockade causes a reduction in inflammatory cell recruitment and infarct size following myocardial ischemia-reperfusion injury *(Pachel C. et al. Arterioscler Thromb Vasc Biol. 2016;36(4):629)*. GPVI has also been identified as a player in platelet interactions with pathogens. For example, staphylococcal superantigen-like 5 induces platelet activation via a mechanism involving GPIb and GPVI *(Hu H. et al Plos One 2011)*. In terms of viruses, hepatitis C virus interacts with human platelets in vivo and native viral particles have been found to bind to the extracellular domain of GPVI *(Zahn A. et al J Gene Virol 2006)*.

Overall, GPVI could therefore be considered as a valuable candidate target to limit the contribution of platelets to uncontrolled lung inflammation without inducing unwanted bleeding.

2. GLENZOCIMAB (ACT017)

Glenzocimab (ACT017) is a fully humanized anti human GPVI fragment of antibody (Fab) *(Lebozec K et al. N Biotechnol 2018)*. Glenzocimab blocks GPVI-triggered platelet activation in vitro. Intravenous administration of glenzocimab is antithrombotic in animal models without inducing bleeding *(Lebozec K et al. Mabs 2017)*. In a placebo-controlled phase 1 clinical trial in 48 healthy subjects, glenzocimab administered via a 6-hr infusion at escalating doses up to 2 g proved to be safe and well tolerated, with no SAE being reported, no dose or time-related adverse event, and no bleeding-time prolongation. Its pharmacokinetic/pharmacodynamic properties have been characterized *(Voors-Pette et al. Arterioscler Thromb Vasc Biol 2019;39:956)*. Glenzocimab is currently being tested in a double-blind phase 2a clinical trial (ACTIMIS NCT 038030037) in patients with a severe acute ischemic stroke on top of the standard of care. Patients are randomly assigned to a placebo or to glenzocimab (125 up to 1000 mg), administered as a 6-hour infusion. This initial escalating dose phase is in progress, and 37 patients have already been enrolled. No specific safety signal has been reported, while the dose of 1000mg has just been reached. A Data

Safety Monitoring Board adjudicates each new patient cohort, and, so far, after 3 scheduled meetings, no specific warning was issued.

COVID-related ARDS represents another disease condition wherein both micro and sometimes macro-thrombotic events are observed together with inflammation, and therefore a potential new, and complementary indication for glenzocimab, consistent with its mode of action.

However, by contrast with acute ischemic stroke where a decisive therapeutic action is required in a matter of hours, a more prolonged pharmacologic activity may be required in ARDS that entails a rapidly progressing process. It therefore requires a different pattern of administration for glenzocimab, so as to cover a minimum of 3 days, though, admittedly this choice is an arbitrary one, in the absence of any precise indicator of when the platelet activation cascade takes place in the pathogenic process.

PK-PD modeling based on pre-existing glenzocimab PK and PD data, has shown that a constant infusion rate of 167 mgh^{-1} for 6 consecutive hours allows for 90% of patients having a ≥80% inhibition of collagen-induced platelet activation for 16 to 18 hrs, a threshold that is arbitrarily admitted as being representative of a full pharmacological effect for GPVI receptor inhibition. Repeating the administration at 24hr-intervals provides the assurance that there would not be any product accumulation in the blood.

2.1 RATIONALE

2.2 PHARMACOLOGY

In vitro, glenzocimab dose-dependently inhibited collagen-induced platelet aggregation with a full inhibition for concentration ≥ 5 µg/mL. In transgenic mice model expressing the hGPVI, glenzocimab showed a dose-dependent inhibition of the ex-vivo aggregation induced by collagen (1 µg/ml of collagen) on concentrated platelet rich plasma (cPRP). Administration of glenzocimab did not modify either the platelet count or induced any modification in hGPVI expression. In cynomolgus monkeys, the minimum effective dose (MED) is 2 mg/kg. Glenzocimab administered as a 15-min injection at 2 to 8 mg/kg doses fully inhibited collagen-induced platelet aggregation 0.5 hrs after the end of the administration. Neither the platelet count nor bleeding time were modified. Slow administration of glenzocimab (1- hr infusion) at 8 mg/kg resulted in a prolonged inhibition of collagen-induced platelet aggregation lasting for 7 hrs, with a total inhibition for all animals lasting 2 hrs. A 6-hr infusion of glenzocimab administered at 8 mg/kg, with ¼ of the dose administered in 15 min and ¾ of the dose administered in 5 hrs 45 min, resulted in a profound inhibition of collagen-induced platelet aggregation lasting 9 hrs. At 24 hrs, the effect of glenzocimab was fully reversed for 3 of the 4 animals.
A model of platelet-dependent intravascular thrombosis induced by a collagen/adrenaline mixture was developed in hGPVI transgenic mice. At 8 mg/kg, glenzocimab has been shown to protect animals from thrombosis.

Co-administration of glenzocimab (8 mg/kg, 6-hr infusion) and tPA (1 mg/kg, 1-hr infusion) in cynomolgus monkeys showed neither impact on rtPA activity nor on its PK profile, as well as no impact on glenzocimab activity on platelet aggregation induced by collagen and no impact on glenzocimab PK profile. ADP-induced platelet aggregation was also not modified either by the co-administration.
In a photochemically-induced thrombotic stroke model in cynomolgus monkeys, administration of glenzocimab (8 mg/kg) resulted in no increase of intracerebral hemorrhage rate and volume. It also showed a trend in favor of a reduced infarct size.

2.2.1 Safety Pharmacology

A study of the safety pharmacology was conducted to evaluate the effect of glenzocimab on the CNS, respiratory and cardiovascular functions. This study was integrated in the pivotal toxicology study (ACT-NCS-002) conducted on Cynomolgus monkeys.
At all doses assessed (8, 25 and 80 mg/kg), glenzocimab had no effects on the CNS (according to the functional observation battery), and on the cardiovascular and respiratory function (assessed with a jacketed external telemetry).

2.2.2 Toxicology

Safety and toxicity of glenzocimab were assessed in two toxicology studies ACT-NCS-001 (ACT-NCS-001 2016) and ACT-NCS-002 (ACT-NCS-002 2017) after a single 6-hr infusion in cynomolgus monkeys. The target pharmacological dose of 8 mg/kg was tested as well as a high dose of 80 mg/kg and an intermediate dose of 25 mg/kg.
No signs of systemic toxicity or bleeding were observed at dose-levels of 8, 25 or 80 mg/kg. Local reactions at the injection site were observed in some animals, mainly on the day following the i.v. infusion and without dose-relationship. Body weight and food consumption were not affected by the treatment. Changes observed in hematological parameters (decreases in red blood cell count, hemoglobin concentration and packed cell volume) were attributed to the several blood samplings collected over a 48-hr period. At blood biochemistry, increases in creatine kinase and lactate dehydrogenase activity observed in almost all control and test item-treated animals were considered to be likely associated with muscle damage caused by the contention in restraint chairs for an extended period of time.
A tissue cross reactivity (TCR) study (ACT-NCS-003) was performed to confirm that glenzocimab does not bind to extra target sites. GPVI expression is limited to platelets and megakaryocytes and therefore no binding to tissues was expected. A panel of 42 tissues and blood smears from 3 unrelated human donors was screened. Binding was observed in blood smears, spleen (red pulp) and bone marrow, which is consistent with platelets and/or megakaryocytes sites. No staining was observed with other tissues. In an *in vitro* hemolysis study conducted with human blood samples, glenzocimab showed no hemolytic potential.
In summary, the results of the toxicity studies in Cynomolgus monkeys have not revealed any potential toxicities that would preclude the use of glenzocimab in a healthy subject. It was well tolerated at doses up to 80 mg/kg. Consequently, a no observed adverse effect level (NOAEL) of 80 mg/kg was extrapolated.

2.2.3 Metabolism in Animals

A dedicated biodistribution study was also conducted in hGPVI transgenic mice using radiolabeled glenzocimab with Iodine-125 to determine its blood, plasma and tissue distribution. Results showed a fast clearance from blood circulation as 98% of the injected dose was eliminated after 3 hrs. The analyzed data tended to confirm a specific binding of glenzocimab to platelets. Activities measured in organs and tissues did not reveal unexpected exposure of the drug and clearly identified that renal elimination was the main route of elimination (it contributed for about 99% of the elimination process), which was consistent with the elimination of a Fab fragment of 48 kDa. A moderate splenic sequestration (less than 3% ID/g) of radiolabeled glenzocimab was highlighted probably due to the presence of platelets. Finally, there was no bone marrow sequestration of the radiolabeled drug, though it could have been expected as bone marrow is the production site for platelets.

In Cynomolgus monkeys, the PK profiles following a 6-hr i.v. infusion in two successive phases (a 15-min loading dose phase of ¼ of the total dose and a maintenance dose phase of ¾ of the total dose), yielded a rapid steady state starting between the first and the third hour after the beginning of the infusion and maintained throughout all of it.

No consistent sex-related differences were reported in Cynomolgus monkeys and the linearity of concentration increments was demonstrated within the dose range of 8 mg/kg to 80 mg/kg. There was no dose-dependency of the total clearance.

An elimination half-life (t½) of around 9.4 hrs and an average total clearance (Clt) of about 90 mL/h/kg were estimated for glenzocimab in Cynomolgus monkeys. At the higher dose level tested (80 mg/kg), considered to be the No Observed Adverse Effect Level (NOAEL), the mean exposure values (males/females), namely C_{max} and $AUC_{0-\infty}$ were determined at 316/263 µg/mL and 989/928 h.µg/mL.

A pharmacokinetic/pharmacodynamics (PK/PD) analysis showed that the estimated 50% inhibitory concentration (IC50) was 0.72 µg/mL (95%CI: 0.47-0.99), confirming the strong potency of glenzocimab to inhibit platelet aggregation in cynomolgus monkeys at the target pharmaciological dose.

2.2.4 Preliminary Clinical Experience: FIH Healthy Subjects Relevant Results

A preliminary FIH study was conducted in healthy subjects from October 30th 2017 through January 2018. The study was designed as a single-center, randomized, double blind, placebo-controlled, single ascending-dose escalation trial. Healthy subjects received either glenzocimab or a placebo as an 6-hr IV infusion, with a 15-min loading dose representing ¼ of the total dose and a maintenance dose phase of 5hrs and 45 min representing the remaining ¾ of the total dose. Total doses of 62.5, 125, 250, 500, 1000, 2000 mg, and a matching placebo were administered. Safety, clinical tolerability, biological safety as well as PK were the outcome parameters. Ex-vivo collagen-induced platelet inhibition as the main pharmacodynamic (PD) parameter was also tested at serial time points.

There were no Serious Adverse Event reported at any of the doses tested, nor was there any dose- or time-related trend in any non-serious adverse effect (AEs). None of the AEs were identified as bleeding related and neither modifications of the hemostatic parameters nor of hemoglobin

concentration were observed. Bleeding time was not affected in a clinically significant manner by any of the glenzocimab doses. The detailed description of all adverse events reported can be found in the Investigator's Brochure.
Results showed that glenzocimab dose-dependently inhibited collagen-induced platelet aggregation. At the dose of 62.5 mg, collagen-induced platelet aggregation was observed in 3 out of 6 subjects, and results returned to baseline 1 hr after dosing. At the dose of 125 mg, platelet aggregation was rapidly inhibited in 4 out of 6 subjects and the effect lasted 6 hrs. At 250 mg, the inhibition was more homogeneous across the subjects (inhibition observed in 5 out of 6 subjects) with an effect lasting around 6 hrs. At the doses of 500 mg, 1000 mg and 2000 mg, the inhibition was observed in all subjects, with an effect lasting around 8 hrs, 18 hrs and 24 hrs, respectively. At 48 hrs, platelets aggregation returned to baseline levels at all dose levels, except for the 2000 mg, for which return to baseline levels was observed at follow-up (Day 7).
For all doses investigated, the applied dosing regimen indeed showed a fast increase in glenzocimab plasma concentrations within the first 15 min of administration. Thereafter, the speed of infusion was reduced, resulting in more or less stable plasma concentrations for the remaining infusion time. Total clearance was constant for all doses. The primary PK parameters C_{max} and AUC, demonstrated dose proportionality across the investigated dose range. Recovery of glenzocimab in urine is only significant (> 1%) at the two highest doses.
These preliminary data concur with those observed in non-human primates.

2.2.5 Ongoing First-in-patient Clinical Trial: ACTIMIS Study

The first-in-patient study in acute stroke combines a phase Ib dose escalation of glenzocimab as add-on therapy to the best standard of care (rtPA +/- thrombectomy) to assess the safety and to identify the recommended phase 2 dose (RP2D), and a phase 2a dose-expansion in order to confirm the safety of the RP2D and to assess glenzocimab potential activity on AIS assessment parameters. Due to its specific mechanism of action showing no evidence of any additional bleeding risk both in animals or in healthy subjects, glenzocimab candidate drug seems to be a promising agent administrable at the acute phase of the stroke in addition to the best standard of care with the following medical management objectives:

1) Reduce the size of the clot,
2) Favor cerebral reperfusion, thus decreasing the infarct volume

Decrease the occurrence of ischemia-reperfusion injury, thereby increasing the percentage of salvageable brain tissue and improving neurological recovery.

2.2.6 Rationale of the Study

To offer an additional treatment option for SARS-CoV-2 infected patients who present with acute respiratory distress syndrome. This treatment might also prevent downstream complications due to pro-thrombotic conditions. If positive, other viral sepsis conditions could benefit from this exploratory approach.

2.2.7 Study Dose Selection

In the ongoing phase 1b/2a clinical trial (ACTIMIS; NCT 038030037) in acute ischemic stroke, glenzocimab is administered in a single 6h infusion with ¼ of the dose administered in a 15-minute bolus injection and the rest of the dose in 5h45.

In the proposed GARDEN study, by contrast with acute ischemic stroke where a decisive therapeutic action is required in a matter of hours, a more prolonged pharmacologic activity may be required in ARDS that entails a rapidly progressing process. It therefore requires a different pattern of administration for glenzocimab, so as to cover a minimum of 3 days, though, admittedly this choice is an arbitrary one, in the absence of any precise indicator of when the platelet activation cascade takes place in the pathogenic process.

The proposed administration is 1000 mg over 6 hours without bolus injection at D1, D2 and D3. This is based on the below PK-PD modeling.

- **PK-PD modeling**

PK-PD modeling based on pre-existing glenzocimab PK and PD data, has shown that a constant 1000 mg infused for 6 consecutive hours allows 90% of patients having a ≥80% inhibition of collagen-induced platelet activation for 16 to 18 hrs, (see graph below), a threshold that is arbitrarily admitted as being representative of a full GPVI receptor pharmacological inhibition.

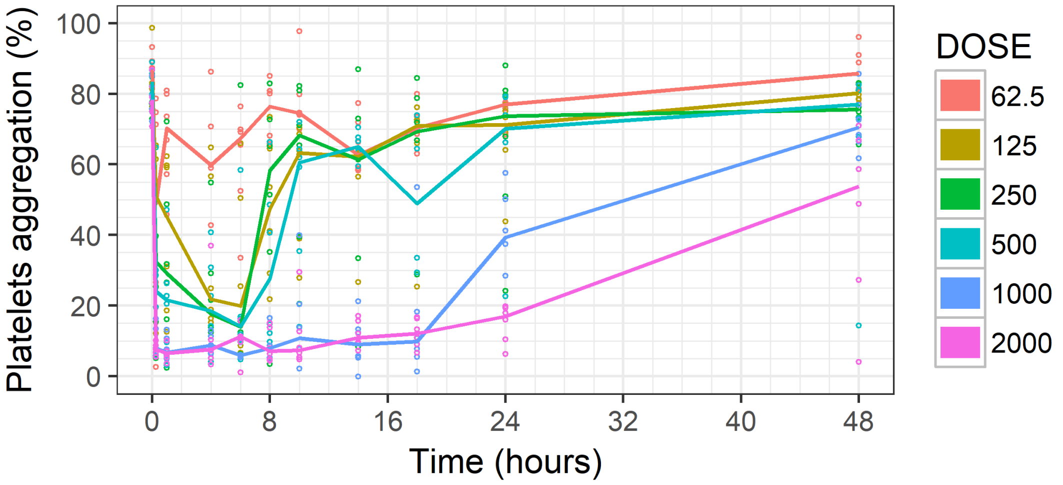

Figure 1: PK/PD model was built on glenzocimab (ACT017) plasma concentration and ex vivo platelet aggregation data from a phase I study in healthy volunteers (median concentration-time profiles by dose overlaid on individual observations - ACT017 LLOQ = 0.3 µg/mL).

From a PK standpoint, glenzocimab concentrations (PK) were determined in the 48-subject escalating dose, phase 1 study in each individual receiving glenzocimab (n=36 subjects), both pre-dose and then 15 minutes, 1, 4, 6, 8, 10, 14, 18, 24 and 48 hours after the start of the infusion. Glenzocimab concentrations in all individuals were also determined at 144 ± 48 hours after the start of infusion. A total of 390 PK observations were included in the PK analysis.

Glenzocimab was found to have dose-proportional, two-compartmental PK with a central distribution volume of 4.1 L, and first and second half-lives of 0.84 and 9.6 hours (see graph below).

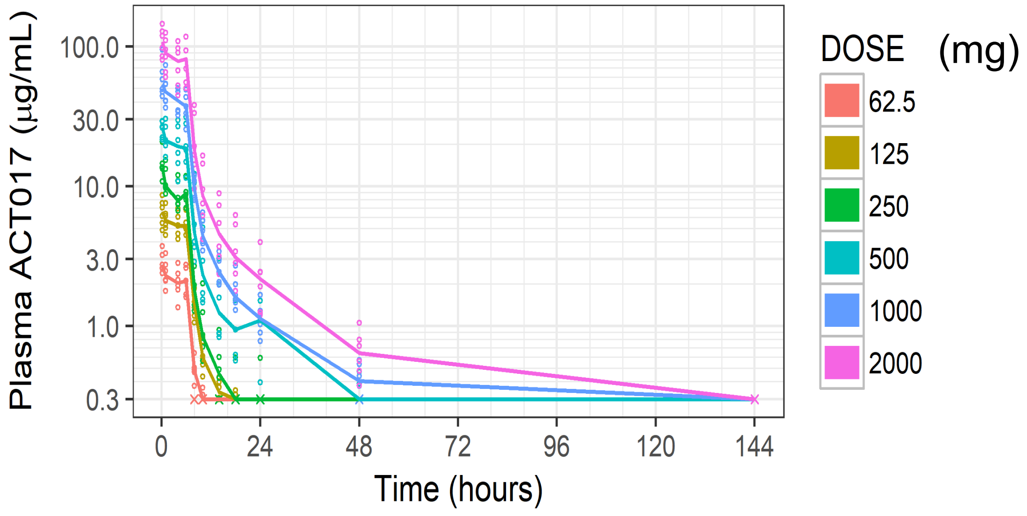

Figure 2: PK/PD model was built on glenzocimab (ACT017) plasma concentration and ex vivo platelet aggregation data from a phase I study in healthy volunteers (median concentration-time profiles by dose overlaid on individual observations - ACT017 LLOQ = 0.3 µg/mL).

On the basis of these preliminary data, a dose of 1000mg infused constantly over a 6-hr period at a constant infusion rate of 167 mg/hr every 24 hrs for 3 dauys was selected for the proposed GARDEN study.

PK simulation (see graph below), upon readministration of 1000mg at 24hr interval, following a 6-hr infusion, shows that less than 1% of the drug can be found in the plasma, making accumulation totally unlikely. Cmax values remain similar over the 3-day period of administration. Based on this, each administration should be regarded as a ‘single’ new administration in the same patient.

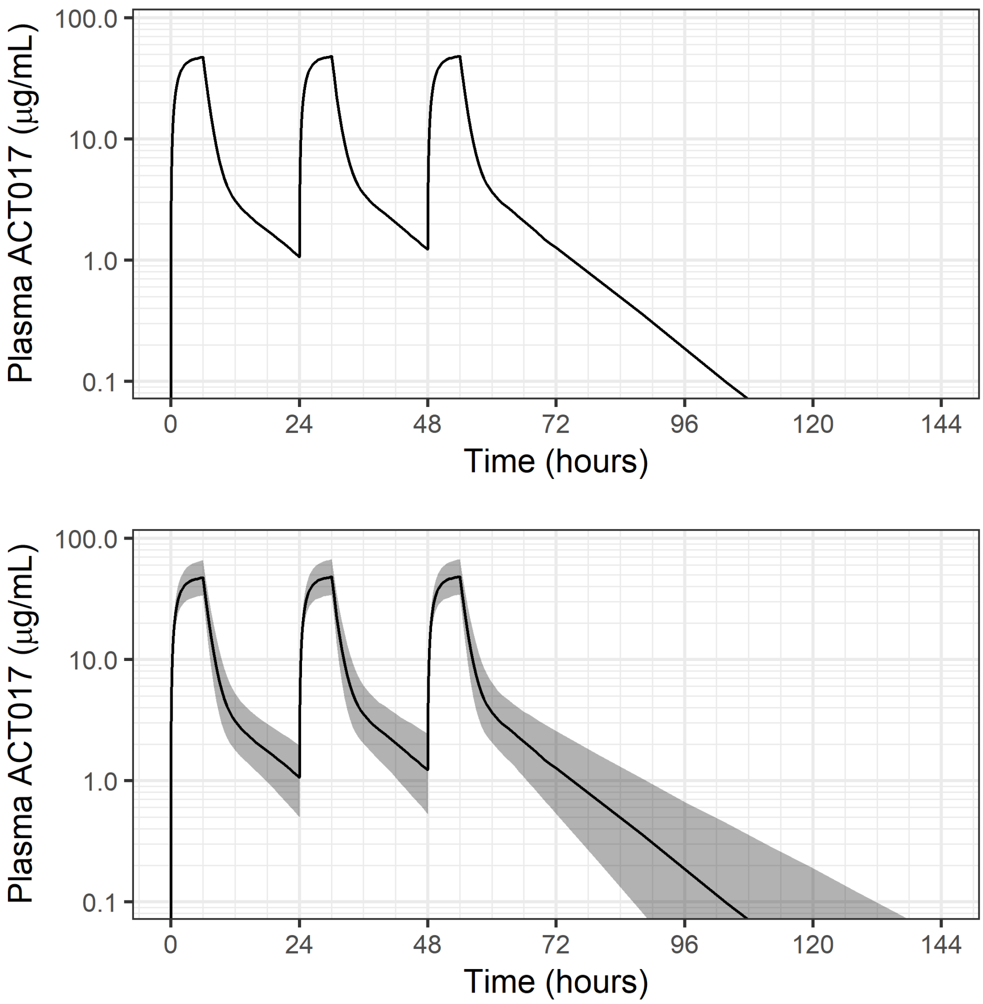

Figure 3: 166.67 mg/h as 6-h IV infusion q1day for 3 days (total dose of 3'000 mg): Predicted plasma glenzocimab (ACT017) concentration (1'000 simulations. Line = median, shaded area = 10th to 90th percentile).

With this pattern of administration, 90% of subjects should have an inhibition of platelet activation between 50 and 95% for the 3-day period of treatment.

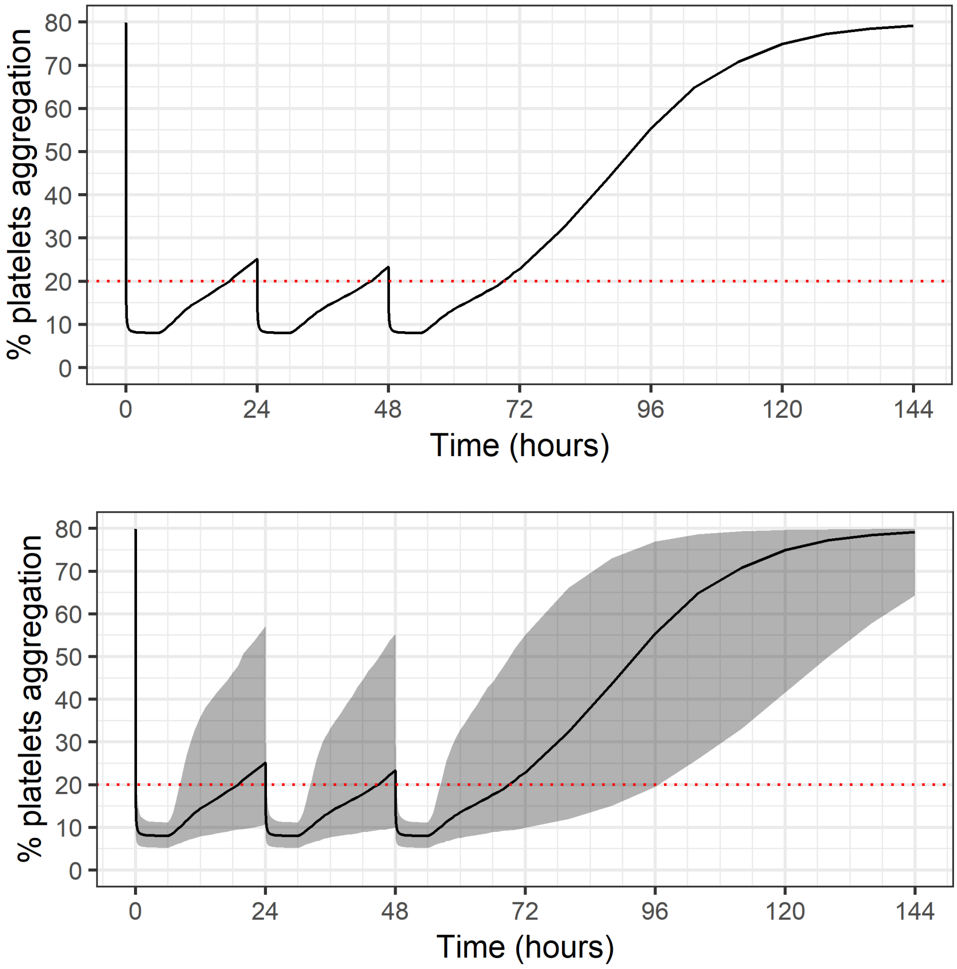

Figure 4: 166.67 mg/h as 6-h IV infusion q1day for 3 days (total dose of 3'000 mg): Predicted inhibition of collagen-induced platelet aggregation (1'000 simulations. Line = median, shaded area = 10th to 90th percentile).

- **Safety data from previous tested doses**

In healthy volunteers, the maximum administered dose was 2000mg over 6 hrs by IV infusion. No dose or time-related adverse event, and no bleeding event, even minor, was reported at any of the dose tested, and the 2000mg dose was considered, by default as the maximuml tolerated dose. No higher dose was tested as 2000mg was considered to be 2 to 4 times superior to the target active pharmacological dose.

In acute ischemic stroke patients, the maximum tested dose is currently 1000mg infused over 6hrs with ¼ of the dose injected as an IV bolus in 15 minutes at the initiation of

the infusion. In this case, 100% of treated patients receive also rtPA as a 0.9mg/kg I-hr infusion, within 4.5 hrs of the onset of stroke symptoms. Glenzocimab infusion initiation should take place less than 1 hr after the end or rtPA infusion, so that both products are present concomitantly in each patient for a period of time varying from 1 to 2 hours. So far the cohort 4 is ongoing, though not all patients have been dosed yet, there has been no specific safety signal attributable to the addition of glenzocimab to rtPA in what regards bleeding events, and no dose-related trend has been noted.

➢ **Immunogenicity**

Regarding immunogenicity, glenzocimab was assessed in silico using the EpiMatrix Protein Immunogenicity Scale from EpiVax. EpiMatrix predicted excess and shortfall in aggregate immunogenicity relative to a random protein standard. All scores are adjusted for the presence of Tregitopes. The submitted glenzocimab sequence scores on the low end of EpiMatrix scale. The combined analysis of glenzocimab heavy and light chains indicates that the immunogenic potential of the antibody is low. As compared to other antibodies, using this established method for calculating immunogenicity, it is estimated that glenzocimab antibody could yield an antitherapeutic response in approximately 2.00% (+/-5%) of exposed subjects.
In healthy volunteers, no dose- or time dependent changes in the immunogenicity ADA data were observed. In the current ACTIMIS study in acute ischemic stroke, no specific safety signal related to the immunogenicity was observed.

➢ **Conclusion**

Based on the above it is therefore the Applicant's opinion that there should be no rationale requiring additional, repeated dose non clinical and clinical (healthy volunteer) studies to support the repeat administration of glenzocimab at 1000 mg over 6hr infusion every 24hrs.

3. STUDY OBJECTIVES

3.1 PRIMARY OBJECTIVE

To evaluate the effect of glenzocimab in preventing clinical progression of disease, when added to Standard-of-Care in Covid-19 patients presenting with a progressive and moderate respiratory distress syndrome.

3.2 SECONDARY OBJECTIVE(S)

3.2.1 Efficacy:

- To assess the impact of treatment on overall disease control,
- To assess the impact of treatment on symptomatology and biological parameters.

3.2.2 Safety:

- To assess the number of the following events:
    - deaths,
    - serious adverse events (SAEs),
    - suspected unexpected serious adverse reactions (SUSARs)
    - medically important events,
    - bleeding-related events.

3.2.3 Pharmacokinetics

- Confirmation of overall PK profile on single and repeated administration (Days 1, 2 and 3), for comparison with initial PK-PD simulation:
    - baseline
    - Cmax: middle of infusion (3hrs post initiation)
    - elimination phase (3 hrs after the end of infusion)
    - residual plasma level: 24 hrs after first infusion initiation (i.e. 18 hrs after the end of infusion)

3.2.4 Exploratory:

- Evolution of pulmonary lesions on chest imaging,
- Evolution of biological parameters related to hemostasis, coagulation and inflammation,
- Determination of predictive factors for a response.

4. STUDY DESCRIPTION

4.1 STUDY DESIGN

A randomized, double blind, multicenter, placebo- controlled, parallel group, fixed dose, phase II study. The study evaluates the efficacy and safety of glenzocimab. Patients will be screened for eligibility and all tests should have results prior to any randomization, so as to avoid screening failures to a maximum extent. The turn-around time for these tests should be comprised within 24hrs to allow for early inclusions if needed. Eligible patients (n=68) will be randomized in a 1:1 ratio to glenzocimab or placebo. Patient inclusions will be fractioned into sequential (3-day apart) cohorts of growing size (2, 4 then 6 patients), each balanced between glenzocimab and placebo in order to check safety in a gradual manner.

Glenzocimab will be administered by IV infusion. The dosing regimen will be 1000mg over 6 hrs, every 24 hrs for 3 consecutive days. All patients will receive in parallel the best medical care at the discretion of the investigating center, or per local guidelines. Unless, patient's condition worsens after the first infusion and/or an untoward adverse drug reaction precludes it, the treatment should be readministered 24 hrs after the initiation of the first infusion, and likewise after the second infusion, so that a total of 3 subsequent infusions, that represent the expected standard treatment should be administered. The allocation of each patient in any given center to an active treatment or placebo will strictly follow a central randomization scheme. Clinical supplies allocation to centers should provide the necessary material so that any eligible patient can receive the assigned treatment. An IxRS will be used to manage randomization and drug shipment. The whole process will be handled in a manner that is blinded for the treatment received to all involved study personnel. The study period will be of a maximum of 40 days per patient. Patients will be closely monitored during the first 7 days following randomization with complete evaluations being performed at 24 hrs, 48 hrs, 72 hrs, then on Days 4 (96 hrs), 5 (120 hrs), and 7 (+/-1 day), 14 (+/-2 days), 20 (+/-2 days), 40 (+/-3 days). Should a patient being discharged before Day 40, a distant consultation should be undertaken if it is not deemed desirable that the patient comes back to the institution.

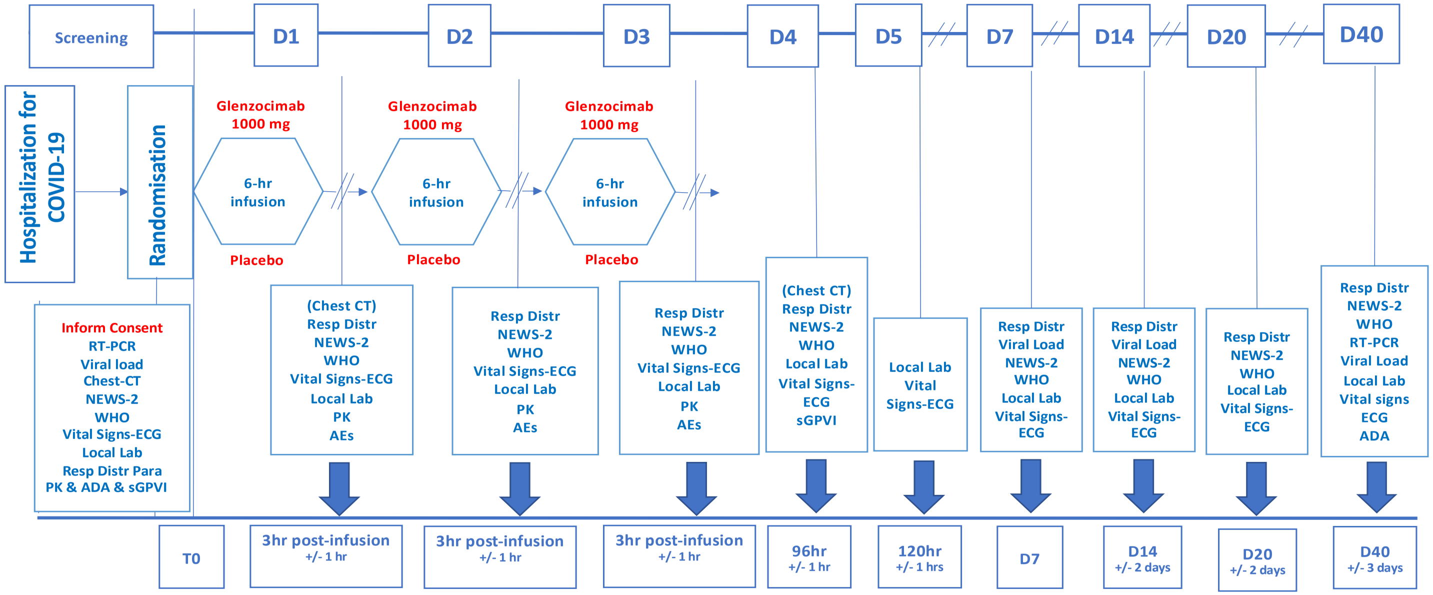

Positive RT-PCR or antigenic test (test previously done accepted), or with a highly positive serology AND clear symptoms of COVID-19
Resp Distr: RR, SpO_2, PaO_2 / FiO_2
PK (10 timepoints): Baseline, 3hr after start of infusion, 3hr post infusion & 24hr post infusion at D1, D2 and D3
ADA (2 timepoints): Baseline and D40
Soluble GPVI (2 timepoints): Baseline and D4
Local Lab: NFS, Platelet, INR/PTT, PT, Fibrinogen, D-Dimers, Serum Glucose, Urea, Creatinemia or GFR, LFTs (ASAT, ALAT), CRP, LDH, IL6, TnT, NT proBNP, Pro-calcitonin, Ferritin
Chest CT scan: Screening, optional at D1 and D4 and on a need basis

Figure 5: Study Design and Schedule of Visits

Patient Safeguards and Early Stopping Rules

Decisions regarding patient's safety are primarily made by the principal investigator and/or under his/her responsibility. This could entail discontinuing the IV infusion, not undertaking the following infusion(s), prescribing any other treatments as warranted by patient's status or evolution, terminating patient's participation to trial. However, in this latter case, close follow-up should be undertaken, notably in the presence of unresolved SAE/SUSAR. In all case, the Sponsor should be informed urgently.

Safety data will be reviewed by an independent Data Safety Monitoring Board (DSMB).

- First review: the first safety review will occur after the first 12 patients enrolled have received 3 days of infusion and have been followed for 3 days after the last infusion.
- Second review: the second safety review will occur after the first 30 patients enrolled have received 3 days of infusion and have been followed for 3 days after the last infusion.
- Additional ad-hoc DSMB meetings may be set up on a need basis, in particular if SAEs/SUSARs were to be recorded, or in case of any unforeseen significant medical event that would warrant an informed safety judgment, as per DSMB charter.
- The Sponsor Medical Team will set up a procedure to monitor this study on an ongoing basis and closely coordinate with the PI, Steering Committee and DSMB in all safety matters.

4.2 STUDY DURATION FOR THE PATIENT

For each given patient, schedule will include:

- A screening assessment upon admission to ensure of diagnosis and inclusion criteria
- At Day 1:
  - a baseline visit followed by a randomization procedure, initiation of first infusion,
  - a post-infusion evaluation at 3 hrs (+/-1hr) after the end of administration.
- At Day 2:
  - Initiation of second infusion (pending results of prior evaluation)
  - A post-infusion evaluation at 3 hrs (+/-1hr) after the end of second administration if a second administration takes place.
- At Day 3:
  - Initiation of third infusion (pending results of prior evaluation)
  - A post-infusion evaluation at 3 hrs (+/-1hr) after the end of third first administration if a third administration takes place.
- At day 4: a follow-up evaluation at 96 hrs (+/- 3 hrs), post beginning of first administration (primary end-point recording).
- At day 5: a follow-up evaluation at 120 hrs (+/-3 hrs), post beginning of first administration.
- In case, the administration is not repeated, post-infusion evaluation at 3hrs at Day 2 and Day 3, Day 4 (96 hrs), Day 5 (120 hrs) visits should take place anyway.
- A follow-up visit at Days 7 (+/-1 day), 14 (+/- 2 days) and 20 (+/-2 days),

- A 40-day (+/-3 days) post-treatment final evaluation, (End-Of- Study visit).

Additional follow-up visits, or phone calls may be required on a need basis notably in case of emerging or prolonged adverse events. Remote visits utilizing specific devices can be undertaken if it is not desirable that the patient, if discharged comes back to the institution.

4.3 PREMATURE STUDY DISCONTINUATION FOR A PATIENT

4.3.1 Discontinuation from Study Treatment

The investigator may permanently terminate the treatment administration (during the course of any of the 3 IV infusions) at any time notably in case of an adverse event judged unacceptable or a worsening of patient's condition (for detailed procedure, please refer to Section 6.3). If the reason for discontinuation is an adverse event, the patient must be followed until resolution or stabilisation of the event (see Section 9.6). In the case of a needed concomitant IV administration of another drug, the study treatment administration might be temporarly suspended, taking carefully note of the infusion stop and re-start times; however, the global study treatment administration must not last beyond 7 hours, including the suspension time-intervals.

4.3.2 Discontinuation from the Study

Patients have the right to withdraw from the study at any time for any reason. Considering the population of patients that will be included, an emergency inclusion process has been integrated to the protocol. In accordance with the requirement of this procedure patients will be asked to consent as soon as they become able to. If a patient decides to not consent at this point, follow-up assessments will be discontinued and therefrom:

- Data collection since the time the patient withdraws his consent will be permanently stopped.
- Data collected from the beginning of the patient's participation to study up to patient's withdrawal of consent will be analysed (except if this is not allowed according to local regulation).

4.3.3 Lost to Follow-Up

Investigators should make every effort to minimize the number of patients lost to follow-up and to obtain all relevant information on those patients, particularly in the search of any AEs. All contact attempts will be documented in the patient's medical records.

4.4 END OF STUDY

Trial termination will be defined as the last defined as last patient last visit and thus the trial is expected to last approximately 7,5 months (6 months of inclusion period plus 40 days of follow-up period). The Sponsor, in accordance with the Study Scientific Committee and/or the DSMB, may decide to stop the trial or part of the trial at any time.

The study may be stopped prematurely by the sponsor in accordance to the recommendation of the Scientific Committee or the DSMB if it is considered that the benefit/risk balance is no longer in favor of the patients (in regard with the study data or new data available coming from

other studies).
The study could also be stopped upon Health Authorities or IECs/IRBs request.

4.5 INDEPENDENT DATA SAFETY MONITORING BOARD (DSMB) AND SAFETY REVIEWS

An independent data safety monitoring board (DSMB) will be set up before the first enrolled patient.

The DSMB will include one clinician (expert in pneumology or infectious diseases), one clinical pharmacologist, and an expert statistician. All of them will be independent of the team in charge of the study conduct.

The primary objective of the DSMB is to review the safety data. At each review, the DSMB will review demographic, baseline, and safety data, including incidence rates of AEs and SAEs regardless of relatedness, events requiring the discontinuation of study drug, notable AEs and laboratory abnormalities, which will be summarized and tabulated by System Organ Class, preferred term, and severity grade prior to each meeting. Listings and/or narratives of "on-study" deaths and serious and significant AEs, including any early withdrawals due to AEs, will be provided. Data to be reviewed will be summarized by treatment arm and will include information on patient accrual, baseline characteristics, and key safety information.

Two safety review meetings are planed:

- First review : the first safety review will occur after the first 12 patients enrolled have received 3 days of infusion and have been followed for 3 days after the last infusion.
- Second review : the second safety review will occur after the first 30 patients enrolled have received 3 days of infusion and have been followed for 3 days after the last infusion.
- In addition to the scheduled meetings, **ad hoc DSMB meetings** for any safety issue can be hold on request of the glenzocimab study team (study investigators, Acticor and PV-CRO). In this regard, inclusions will be fractioned into sequential (3-day apart) cohorts of growing size, each balanced between glenzocimab and placebo in order to check safety in a gradual manner. In addition, a complete independent medical monitoring will occure after the 2 first patients, then the following 4 patients, then the next 6 patients.

Based on these safety data, the DSMB will make recommendations to the Sponsor as to whether the trial should continue without changes, be modified, or be stopped due to safety concerns. If the study is terminated early based on the DSMB recommendation, ACTICOR will notify the appropriate regulatory authorities.

Further details on the interim analyses and the responsibilities of the DSMB will be provided in the DSMB Charter.

4.6 OVERSIGHT COMMITTEES

A study scientific dedicated meeting was set up in order to validate the most important protocol choices.
Attendees were 1) clinicians from various countries, who have a strong recognized expertise in the field of infectious or pulmonary diseases, 2) Clinical pharmacologists and/or methodologists. During this meeting, there were also representatives from the Sponsor.

1) A Study Steering Committee comprised of members of the Sponsor, as well as a few of the Scientific Advisors, and the Study Global Coordinator will be responsible for the permanent study oversight. It is co-chaired by the Global Study Coordinator and [redacted], Infectious Diseases Specialist, Executive Medical Advisor to Acticor Biotech.

The Steering Committee may require the intermittent or permanent recourse to ad-hoc experts as needed.

4.6.1 Mission of the Study Steering Committee

The Study Steering Committee will be the oversight and decision body for this trial, though the liability for final decisions will remain that of sponsor, as per GCP and regulations.
The study steering committee will meet a need basis depending upon DSMB preconisation, and the necessity to discuss, make or endorse decisions. Should some face-to-face meetings be impossible to hold due to practical constraints, virtual (e.g. phone / webex) meetings should take place. In any case, whatever the modality, detailed minutes and decisions should be documented in writing.
The DSMB may ask specific questions to the Steering Committee, and vice-versa. In the event, rapid responses are required, Steering Committee Chairs should be contacted first. In any case, the DSMB will be responsible to preserve study integrity in its communicating to the Steering Committee, particularly so as the blind is maintained.

5. STUDY POPULATION

5.1 ELIGIBILITY CRITERIA

A total of 60 evaluable adult patients, hospitalized and presenting with acute respiratory distress syndrome that meet the following study criteria will be enrolled to the study.

5.1.1 Inclusion Criteria

1. Male or female hospitalized patients ≥ 18 years (i.e., at least 18 years old at the time of randomization) and < 80 years, having given their written consent.

2. Having a positive RT-PCR or antigenic test for COVID-19, or with a highly positive serology AND clear symptoms of COVID-19,

3. Presenting with symptoms of COVID-19, including:
   - Cough

   OR

- Shortness of breath or difficulty breathing
  OR at least 2 of the following
- Fever, defined as any body temperature 38°C
- Chills
- Repeated shaking with chills
- Muscle pain
- Headache
- Sore throat
- New loss of taste or smell

4. Presenting with signs of moderate but progressive pulmonary disease in the last 48 hours with:
   - Respiratory symptoms (cough, dyspnea, etc.),
   - Uni- or bilateral ground-glass opacities, or pulmonary infiltrates on chest radiograph and/or CT scan performed within the past 96hrs,
   - Clinical and/or biological evidence of progression over the past 48hrs.
   - Progression will be assessed as follows:
     - o symptom worsening: increase in the number of symptoms, and/or in the intensity of some of them
       And/or
     - o worsening of respiratory condition: increase in respiratory rate and/or decrease of Pa02/Fi02 in ambient air
       And/or
     - o worsening of biological markers: CRP increase or increase of other severity /prognostic markers, and/or increase of D-Dimers

5. Presenting with a **moderate** respiratory distress defined as:

   a) At least one of the following signs associated with the onset of ARDS:

   - Respiratory rate (RR) $\geq$ 24/min,
     OR
     - $SpO_2 \leq 93\%$ in ambient air. In case where oxygenotherapy cannot be discontinued, please refer to NEWS 2 Scale (item SpO2, scale 2) for adequate conversion (e.g. 93-94 on O2 corresponding to 86-87 in ambient air),

     OR
   - $PaO_2/FiO_2 \leq 200$mmHg (please refer to Appendix 3 for conversion).

   AND

   b) NOT in a **severe** respiratory distress defined as:

   - Respiratory rate (RR) < 30/min,

   And

   - $PaO_2/FiO_2 > 100$mmHg.

6. Presenting with signs of a pro-thrombotic status characterized by:

a. D-Dimers ≥ 0.5 µg/mL,
b. and/or Troponin T >2.5 µg/L(or by default Troponin I greater than local laboratory reference),
c. and/or signs of micro-angiopathy on a vascular enhanced chest CT-scan. *(Thrombocytopenia <150,000/mm^3 or prolonged Prothrombin Time (PT) >12s are additional signs of a pro-thrombotic status that are not necessary for eligibility).*

7. With one or more of the following biological markers of progression:
    - CRP ≥10 mg/L,
    - LDH > 250 U/L,
    - IL6 > 8 pg/mL,
    - Lymphocyte count <1 10^9/L,
    - NT proBNP > 88 pg/mL,
    - Pro-calcitonin > 0.5 ng/mL,
    - Ferritin > 400 µg/L,

8. Effective birth control that should have been in place for at least 2 months in non-menopausal women and 4 months for men after IMP administration. Birth control methods considered to be highly effective include:
    - Combined (estrogen-progestogen) hormonal contraception associated with the inhibition of ovulation: oral, intravaginal, transdermal,
    - Progesterone-only hormonal contraception associated with the inhibition of ovulation: oral, injectable, implantable,
    - Intrauterine device,
    - Intrauterine hormone-releasing system,
    - Bilateral tubal occlusion,
    - Vasectomized partner.

9. Women of child-bearing potential must have negative results of a urinary or plasma pregnancy test (serum HCG).

5.1.2 Non-Inclusion Criteria

1. Patients requiring invasive/assisted mechanical ventilation (intubation),
2. Obvious disseminated intravascular coagulation (DIC), (with e.g. a variable combination of the following: low platelet count (<100,000/mL), prolonged PT> 12sec and/or aPTT>60sec, presence of fibrin degradation products in the plasma, with or without clinically visible hemorrhagic signs). An isolated change of one of these parameters does not qualify for DIC,
3. ARDS of another origin,

4. Concomitant pulmonary infection (pneumoniae) with another agent, notably bacterial or fungal,
5. Patients presenting with hemoglobin < 9g/dL,
6. Patients under immunosuppressive agents,
7. Patients receiving an anti-cancer treatment (radiotherapy, chemotherapy, immunotherapy)
8. Initiation of a treatment with aspirine (previous stable preventative aspirin regimen from 75 to 160 mg per day is allowed),
9. Patients under anticoagulant therapy (except heparin and low-molecular weight heparin), and anti-Xa drugs achieving effective anticoagulation, as assessed by appropriate tests, or having received thrombolytics ≤24 hrs,
10. Patients receiving NSAIDs or anti-platelet agents with platelet suppression within the past 7 days,
11. Patients treated concomitantly with another monoclonal antibody (e.g. tocilizumab),
12. Ischemic stroke or transient ischemic attack within the past year,
13. Deep venous thrombosis or pulmonary embolism within the past year,

14. Severe renal insufficiency (Grades 4-5) with a glomerular filtration rate <30mL/Min/1.73m^2,
15. One of the following severe organ failures:
    - Hepatic with either Child Pugh score ≥C, or ASAT/ALAT ≥5 U.N.L,
    - Cardiac with NYHA≥Class II, unstable angina pectoris, myocardial infarct <1 year, supra-ventricular or ventricular arrhythmia,
16. Hereditary tendency to bleeding or coagulopathy,
17. Severe vascular disease (aneurysms, arterial surgery ≤6 months),
18. Unhealed wounds, gastrointestinal ulcers or perforation ≤6 months,
19. Major surgery <28 days, other surgery within the past 7 days,
20. Hemoptysis, GI bleeding, CNS bleeding <1 month,
21. Platelet count <50,000/mm3 (50 g/L),
22. Absolute Neutrophil Count ≤1,000/mm3 (1.0 g/L),
23. Terminal illness, including cancer (life expectancy <3 months),
24. Uncontrolled arterial hypertension (systolic blood pressure ≥185 mmHg and/or diastolic blood pressure ≥110 mmHg despite appropriate antihypertensive therapy,
25. Childbirth within <10 days,
26. Pregnancy or breastfeeding,
27. Prior cardiopulmonary resuscitation <10 days,
28. Allergy or hypersensitivity to drugs of the same class,
29. Participation in another interventional clinical trial 30 days prior to the study inclusion.

5.2 PATIENT IDENTIFICATION

Each patient will be identified with a specific patient's study number assigned by the central randomization system.

5.3 SCREEN FAILURES

Screen failures are defined as patients who consent to participate in the clinical study or for whom an emergency consent procedure is performed but are not subsequently randomized to any study treatment. A minimal set of screen failure information is required in the eCRF to ensure transparent reporting of screen failure participants, to meet the Consolidated Standards of Reporting Trials (CONSORT) publishing requirements, and to respond to queries from regulatory authorities. Minimal information includes date of consent, demography, screen failure details, eligibility criteria, and any serious AEs.

5.4 EVALUABLE PATIENTS

Any patient having received at least one of the three scheduled doses of the investigational drug followed by a clinical evaluation performed after administration (i.e. so long as the infusion has started) will be evaluable for both efficacy and safety.
Based on this definition, a minimum of 60 patients evaluable for efficacy will be required. Assuming a dropout rate of 12%, 68 patients will be enrolled in this study.

More details on the evaluation of populations will be provided in the statistical analysis plan.

6. INVESTIGATIONAL MEDICAL PRODUCT

6.1 ACT017 OR MATCHING PLACEBO

6.1.1 Glenzocimab (ACT017)

Glenzocimab is formulated for IV administration as a sterile product with 20 mM sodium citrate and 130 mM sodium chloride buffer at pH of 5.0. It is supplied for clinical trial use in vials containing 50 mL of the drug product at a concentration of 10 mg/mL. Each vial contains 500 mg of glenzocimab. No preservative is used since the vial is designed for single use. The primary packaging is a type 1 glass vial with an inert bromobutyl rubber stopper and sealed with a plastic flip-off cap.
For further details regarding the physico-chemical and pharmaceutical properties and formulation of glenzocimab, please refer to the IB in its last applicable version.
Glenzocimab is a solution for dilution, and the required amount of glenzocimab should be diluted in 0.9% Sodium Chloride. The infusion should be prepared according to the protocol described in the Pharmacy Manual.
Each unit box and vial is labelled, and labels will use a multi-language text related to all countries involved and must comply with the legal requirements of each applicable country. They include drug storage conditions for the drug and batch number.

6.1.2 Glenzocimab (ACT017) Matching Placebo

The matching placebo will be supplied for clinical trial use in vials containing 50 mL of a solution of a 0.9% sodium chloride. Similarly to the active drug ACT017, each unit box and vial is labelled following the same requirements.

6.2 MANAGEMENT OF IMP

6.2.1 Shipment and Receipt

The study drug (glenzocimab and matching placebo) are provided and sent by the Sponsor or its representatives to each investigational site. Administration kits (bags and tubing) are also provided and sent by the Sponsor or its representatives.
The Hospital Pharmacist or the appropriate person will be contacted prior to each shipment in order to ensure that receipt takes place under the best possible conditions.
Upon receipt of treatment supplies, the Hospital Pharmacist or the appropriate person will inventory the supplies and send the acknowledgement of receipt to the sponsor or its representatives. Should any abnormality of the supply boxes be observed, the Hospital Pharmacist or the appropriate person must immediately inform the Sponsor and quarantine the

supplies under appropriate storage conditions (+2 to +8°C) until instructions received by sponsor to destroy, keep in quarantine or safe to use drug/placebo.
The Pharmacist or the appropriate person must fill in the accountability forms with information concerning the reception of the study drug (glenzocimab and the matching placebo).

6.2.2 Storage Condition

The IMP (glenzocimab or matching placebo) should be stored in the pharmacy or in an appropriate secured limited-access area. Each vial should be kept in the outer carton, in order to protect from light.
Glenzocimab and matching placebo should be stored under appropriate storage conditions (+2 to +8°C) in accordance to the information available in the last applicable version of the IB.

6.2.3 Dispensing and Administration of IMP

Under no circumstances should the Investigator allow the study drug to be used other than as directed in the protocol.
For each administration, the Investigator will specify to the Hospital Pharmacist or his/her representative the exact dose to be administered to the patient and complete a prescription for the study drug.
It is the Investigator's responsibility to ensure that an accurate record of the investigational product issued and returned is maintained.

6.2.4 IMP Preparation and Dispensation

The study nurse or physician will prepare the glenzocimab as per the instructions of the Pharmacy Manual.
The study nurse or coordinator will complete the accountability forms with information concerning the dispensation of the study drug (glenzocimab or placebo).
Preparation of glenzocimab infusion bags will be done as per the IxRS Instructions following randomization in the system, by the study nurse or physician of the stroke unit according to instructions in the pharmacy manual for study drug.

6.2.5 Dose and Administration

Glenzocimab vials must be checked before being administered for transparency, color, aspect or possible deposits.
IMP must not be administered if it does not conform to the analytical references provided in terms of verifiable physical appearance. The Investigator or his/her representative must immediately inform the Monitor.
The administrations of IMP must be done under close supervision of a physician or qualified delegated staff, experienced in clinical trial management at the investigational center.
Treatment will be administered as a single dose in compliance with the dose corresponding to the dose allocated by central randomization to cohort in which the patient is included.
Glenzocimab or the matching placebo is intended to be administered as a 6-hr IV infusion.

6.3 IMP ALLOCATION

6.3.1 Randomization

Centralized randomization will be used in order to check eligibility and ship the IMP to the investigating site to allow patient's treatment.

- The allocation of the next patient in row in any given center to an active dose or placebo will have to strictly comply with this central randomization scheme. During the whole trial, patients will be randomized to a 1:1 ratio between active and placebo. One reference site will start the Inclusions as follow: inclusions will be fractioned into sequential (3-day apart) cohorts of growing size (2, 4 then 6 patients), each balanced between glenzocimab and placebo in order to check safety in a gradual manner.

Randomization will be done electronically in the IxRS. Procedure will be described separately in the dedicated manual.

6.3.2 Double-Blinding

The study will be performed in a double-blind fashion. The Investigator and study staff, the patient, the monitors and the Sponsor's staff and the DSMB will remain blinded to the treatment throughout the study unless DSMB requires an unblinded review for safety concerns. The study drug and its matching placebo are indistinguishable. Indeed, each center will be provided with numbered non-identifying vials of glenzocimab treatment or matching placebo and all subject kits will be packaged in the same way. No additional blinding material is required.

6.3.3 Unblinding Procedure

Blinding should be maintained strictly in order to ensure the reliability of the data obtained until final analysis. Nevertheless, in case of emergency (including suspected misuse or overdose) where patient management would be improved by the knowledge of the blinded treatment assignment, the investigator or one of its representative should immediately follow the unblinding procedures by connecting to the dedicated unblinding website. The Sponsor must be informed of a unblinding procedure as soon as possible before or after the code break.
The DSMB will also be authorized to require for unblinding after discussion with the sponsor at any time point. In this case, a dedicated unblinded statistician will transfer unblinded data to the DSMB statistician, but other study personnel should remain blinded.

6.4 COMPLIANCE AND TREATMENT ACCOUNTABILITY

Treatment compliance is defined as the IV administration of the study drug at prescribed doses and volumes. Prescribed doses and total volume administered will be recorded in the eCRF.
Study drug is to be administered only under the direct supervision of a physician familiar with the requirements of the study protocol, therefore compliance should not be an issue. Nevertheless, the reason for any non-compliance must be recorded in the eCRF.
All study treatments must be recorded on the study treatment batch accountability forms provided by the sponsor or its representative. The investigator, or designee, must ensure the input of the correct information on the study treatment batch accountability forms.
The Investigator or the appropriate person must complete the accountability forms with information concerning the administration of study drug.

The accountability forms should be filed in the Investigator site file. The Monitor will verify the drug accountability forms for completeness and accuracy at each site visit until the end of treatment period of the patients.
At the end of the study, it must be possible to reconcile delivery records with dispensing records and used/unused vials. Any discrepancy must be accounted for.
Used/unused vials of study drug should be kept in a secure place at the site until the accountability forms have been verified by the Monitor.
The Investigator or the Hospital Pharmacist or her/his representative is responsible for the traceability of study drug specifically used during the trial and must retain the documents for 15 years or per local requirements.

6.5 MANAGEMENT OF INFUSION RELATED REACTION (IRR) AND HYPERSENTIVITY REACTIONS

There are no expected acute Infusion-Related Reactions (IRR) to glenzocimab, based on previous human experience. However, a special attention should be paid to bleeding type events as well as to immunologic reactions (see Section 9.4.2).

No premedication is planned for the first patients treated with glenzocimab. Should an IRR of grade > 2 or + related to glenzocimab be observed in one patient, systematic premedication with paracetamol and antihistamines 30 min before infusion will be mandated for all subsequent patients.

A low rate of infusion, gradually increasing based on patient's behavior would be used. A careful observation of patients will be made from the beginning Blood pressure and heart rate monitoring by a qualified nurse or physician should be measured every 30 min during the infusion glenzocimab, so that immediate action can be taken in response to symptoms of an adverse reaction. Facilities and equipment for resuscitation should be immediately available with regards with the unit specialized in the indication.

In case of AEs occurrence during the infusion, the patients must be treated according to investigator's judgment and best clinical practice. The infusion rate will be paused or the infusion rate decreased, which may cause longer than anticipated infusion time.

In case of an Infusion Related Reaction Grade 1-2 (NCI-CTCAE v5.0), the infusion should be paused. When the patient is stable, the infusion can be restarted at the investigator's judgment. Upon restart, the infusion rate should be half of the infusion rate applied before the pause.

In case of an Infusion Related Reaction Grade ≥3, the infusion must be stopped and the patient observed carefully until resolution of the AE. If the patient experience a grade 3 and/or life threatening event of bronchospasm or laryngeal edema the patient must be withdrawn from treatment. If the intensity of any other AE is still grade 3 and/or life threatening after 2 hrs the patient must be withdrawn from treatment. If the intensity of any other AE decreases to grade ≤ 2 within 2 hrs, the infusion can be restarted at investigator's judgment.
Refer to Section 9.7.2 regarding information on reporting of SAEs, SUSARs or serious safety-related protocol deviations and measures for prompt communication.

6.6 EMERGENCY PROCEDURES REGARDING SAEs

Although it is not expected from both non-clinical and clinical phase 1 observations to anticipate SAEs related to glenzocimab administration, both the potential severity of the cases treated, and the standard of care added to the investigational drug might result in unexpected

medical situations. Hemorrhage, hemorrhagic transformation of the infarct zone could potentially be observed in an increased number of cases.

It is reminded, that the primary recourse for patients is the investigator and his/her representatives. These latter may decide to discontinue the infusion in the event patient deteriorates, and undertake any emergency measure they deem to be necessary.

There is no direct antidote to glenzocimab. However, patient's platelets remain normally aggregable to ADP and other pro-thrombotic agents.

The early dispatch to the monitor in charge, to the Pharmacovigilance group (AIXIAL), will help inform the DSMB members, the Study Scientific Committee and the Sponsor, so that any decision pertaining to the safe conduct of the study be taken with no delay.
The SDEA established between AIXIAL and ACTICOR will describe in details the various information flows and reporting obligations of the various parties involved. The detailed flowchart of the steps for handling ACT-CS-006 SAEs is included in.
Refer also to Appendix 5.

It will be then the Sponsor's responsibility to communicate with the Competent Authorities, should there be a matter for them to be informed, on top of pharmacovigilance reporting.

The Sponsor is also amenable to put the study on hold prior to informing the Authorities, as a conservative measure, if warranted.

6.7 CONCOMITANT MEDICATIONS

6.7.1 Non-Authorized Concomitant Treatments

Patients should not receive any other experimental therapies.
In addition to the aforementioned exclusion criteria, patients should not receive any of the following:

- Anticoagulants whether oral or injectable, with exception of heparin and low-molecular weight heparin will not be authorized during all treatment as well as during the first 24 hrs post-glenzocimab last administration.
- Anti-platelet agents whether oral or injectable, will not be authorized during all treatment as well as during the first 24 hrs post-glenzocimab last administration,
- Any off-label treatment, exept if officially recommended for COVID-19 treatment as SOC.

6.7.2 Authorized Treatments

Symptomatic treatments routinely used for disease-related symptoms will be allowed to all patients on this trial. Specific treatments for any adverse events will be allowed too.

The use of both antibiotics and antiviral agents will be authorized.

The use of treatments recently recommended for the treatment of SARS-CoV-2 infection is permitted. This includes corticosteroids. However, the use of other monoclonal antibodies, even recommended is not permitted.

The use of unfractionned heparin or low-molecular weight heparin is allowed.

Details of any treatments or procedures must be recorded in the eCRF.

7. SCHEDULE OF ASSESSEMENTS

7.1 STUDY FLOWCHART

ACT-CS-006 - Garden Study - Study Flowchart

Visit No.	Visit 1	Visit 2 Day 1			Visit 3 Day 2		Visit 4 Day 3		Visit 5 Day 4	Visit 6 Day 5	Visit 7 Day 7	Visit 8 Day 14	Visit 9 Day 20	Visit 10 Day 40	Unscheduled At any day/time if required
Visit Name / Timelines	Screening	Baseline within 24hrs	1st infusion 6 hr IV infusion	Evaluation at 3hrs post 1st infusion (+/- 1hr)	2nd infusion 6 hr IV infusion	Evaluation at 3hrs post 2nd infusion (+/- 1hr)	3rd infusion 6 hr IV infusion	Evaluation at 3hrs post 3rd infusion (+/- 1hr)	Evaluation at 96hrs (+/- 1hr)	Evaluation at 120hrs (+/- 1hr)	(+/- 1hr)	(+/- 2 days)	(+/- 2 days)	End of Study (+/- 3 days)	
Informed Consent	X														
Eligibility criteria	X	X													
Demographics Medical/Surgical History	X														
Concomitant Medication	X	X	X	X	X	X	X	X	X	X	X	X	X	X	X
RT-PCR	X													X	
Viral Load	X										X	X		X	
Chest CT-Scan	X	X (1)		X (1)					X(1)						X
Respiratory Distress parameters (2)	X	X		X		X		X	X		X	X	X	X	
NEWS-2 & WHO Scales		X		X		X		X	X		X	X	X	X	
IP/Placebo IV administration			X		X		X								
PK		prior infusion	3hr after start	3h after end	Prior infusion & 3h after start	3h after end	Prior infusion & 3h after start	3h after end	24h after start of 3rd infusion						
ADA		X												X	
Soluble GPVI		X							X						
12-lead ECG	X			X		X		X	X	X	X	X	X	X	
Clinical evaluation	X			X		X		X	X	X	X	X	X	X	X
Vital Signs	X	X	X (3)	X (3)	X (3)	X (3)	X (3)	X (3)			X	X	X	X	X
Local blood sample (4)	X	X		X		X		X	X	X	X	X	X	X	X
Urine or serum pregnancy test	X													X	
Urinalysis (5)	X (5)			X		X		X				X			
IxRS (randomisation & kit allocation)		X		X		X									
Adverse Event / SAE	X	X	X	X	X	X	X	X	X	X	X	X	X	X	X

(1) Chest CT-Scan In exceptional cases, chest radiogram should not be repeated if previous CT scan done within 48hrs prior to Baseline visit
D1 and D4 optional

(2) Respiratory Distress parameters Respiratory Rate (RR), Oxygen Saturation (SpO2), Ratio PaO2/FiO2

(3) Vital Signs Blood Pressure and Heart Rate will be assessed every 30 min during the 6h (infusion) then 3 hours up to 24 h

(4) Local blood sample
Hematology: NFS, Platelet
Coagulation: INR, aPTT, PT
Biologigcal markers: D-dimers, CRP, TnT, Fibrinogen, IL6, NT proBNP, Pro-calcitonin, Ferritin
Blood Chemistry: GFR, Urea, Serum glucose, ASAT, ALAT, LDH

(5) Urinalysis dipstick or lab urinalysis: urobilinogen, glucose, protein

7.2 SCREENING

Screening period will start as soon as the written informed consent is signed, or the consent is obtained as per the local requirement for emergency inclusion procedure, the following examination will be performed before randomization:

- Informed consent form;
- Demographic data (age, gender…);
- Relevant Medical history;
- Eligibility criteria;
- Positive RT-PCR or antigenic test (test previously done accepted), or with a highly positive serology AND clear symptoms of COVID-19;
- Viral load test;
- Radiograph/ Chest CT scan (no more than 96hrs prior to randomization);
- Clinical evaluation / physical exam including height and weight (weight may be estimated by PI or delegate as patient's condition may prevent proper weighing at this time;
- Vital signs (BP, Pulse);
- Respiratory Distress Parameters
    - Respiratory Rate (RR),
    - Oxygen saturation (SpO_2),
    - PaO_2/FiO_2
- 12-lead ECG;
- Local blood sample test;
- Urine or serum pregnancy test;
- Urinalysis;
- Concomitant medication;

7.3 BASELINE / RANDOMIZATION

Randomization will be performed automatically via the IxRS by the site. Detailed procedure will be described in the dedicated instruction manual. The following examination will be performed before randomization. General and specific examinations and tests that are part of the standard-of-care for Covid-19 management are not study specifics and results can be used for the screening/eligibility assessment of the patients before the signature of the informed consent.

- Validation of eligibility criteria assessed during screening visit
- Vital signs (BP, Pulse);1-time-point pharmacokinetics (baseline);
- Blood sample for Anti-glenzocimab antibodies (ADA) assessments will be collected and sent to the central laboratory;

- Soluble GPVI will be collected and sent to the central laboratory;
- Local blood sample test;
- Respiratory distress parameters:
  - Respiratory Rate (RR),
  - Oxygen saturation (SpO_2),
  - PaO_2/FiO_2 (please refer to Appendix 3 for conversion);
- Chest CT-scan (to be repeated only if the previous one done more than 48 hrs prior to randomization);
- NEWS-2 and/or WHO scales;
- Concomitant medication;
- Recording of adverse events according to NCI-CTCAE;
- IxRS (Randomisation and Day 1 kit allocation).

The randomization visit should be performed within 24 hrs after the screening visit, once all eligibility criteria have been collected and checked.

7.4 TREATMENT PERIOD: 1st INFUSION AND DAY-1 EVALUATION (3 hrs (+/-1 hr) after the end of the first infusion)

Glenzocimab or the matching placebo is intended to be administered as a 6 hr IV infusion. The following examination will be performed:

- Clinical evaluation;
- Vital signs;
- 12-lead ECG;
- Pharmacokinetics: 3 sampling time-points:
  - Baseline, before the study drug administration,
  - 3 hrs after the beginning of the study drug administration,
  - 3 hrs after the end of the study drug administration,
- Local blood sample test;
- Respiratory distress parameters:
  - Respiratory Rate (RR),
  - Oxygen saturation (SpO_2),
  - PaO_2/FiO_2;
- NEWS-2 and/or WHO scales;
- Optional Chest CT-scan;
- Urinalysis;
- Concomitant medication;
- Recording of adverse events according to NCI-CTCAE;
- IxRS for Day 2 kit allocation.

For the 1-day evaluation, a window of ± 1 hr is allowed.

7.5 TREATMENT PERIOD: 2nd INFUSION and DAY-2 EVALUATION (3 hrs (+/-1 hr) after the end of the second infusion)

The following examinations will be performed:

- Clinical evaluation;
- Vital signs;
- 12-lead ECG;
- Pharmacokinetics: 3-sampling time-points:
  - Before treatment initiation (i.e. 24 hrs after first infusion initiation)
  - 3 hrs after the beginning of the study drug administration,
  - 3 hrs after the end of the study drug administration,
- Local blood sample test;
- Respiratory distress parameters:
  - Respiratory Rate (RR),
  - Oxygen saturation (SpO_2),
  - PaO_2/FiO_2;
- NEWS-2 and/or WHO scales;
- Urinalysis;
- Concomitant medication;
- Recording of adverse events according to NCI-CTCAE;
- IxRS for Day 3 kit allocation.

For the 2-day evaluation, a window of ± 1 hr is allowed.

7.6 TREATMENT PERIOD: 3rd INFUSION and DAY-3 EVALUATION (3 hrs (+/-1 hr) after the end of the third infusion)

The following examinations will be performed:

- Clinical evaluation;
- Vital signs;
- 12-lead ECG;
- Pharmacokinetics: 3 sampling time-points:
  - Before treatment initiation (i.e. 24 hrs after the second infusion initiation)
  - 3 hrs after the beginning of the study drug administration,
  - 3 hrs after the end of the study drug administration,

- Local blood sample test;
- Respiratory distress parameters:
  - Respiratory Rate (RR),
  - Oxygen saturation (SpO_2),
  - PaO_2/FiO_2;
- NEWS-2 and/or WHO scales;
- Urinalysis;
- Concomitant medication;
- Recording of adverse events according to NCI-CTCAE.

For the 3-day evaluation, a window of ± 1 hr is allowed.

7.7 DAY-4 EVALUATION (96 hrs)

Day 4, is the Day where the Primary efficacy endpoint is recorded

The following examinations will be performed:

- Clinical evaluation;
- 12-lead ECG;
- Respiratory distress parameters:
  - Respiratory Rate (RR),
  - Oxygen saturation (SpO_2),
  - PaO_2/FiO_2;
- NEWS-2 and/or WHO scales;
- Optional Chest CT-scan;
- Local blood sample test;
- Soluble GPVI;
- Concomitant medication;
- Recording of adverse events according to NCI-CTCAE.

For the 4-day evaluation, a window of ± 1 hr is allowed.

7.8 DAY-5 EVALUATION (120 hrs)

The following examinations will be performed:

- Clinical evaluation;
- 12-lead ECG;
- Local blood sample test;
- Concomitant medication;
- Recording of adverse events according to NCI-CTCAE.

For the 5-day evaluation, a window of ± 1 hr is allowed.

7.9 DAY-7 EVALUATION

The following examinations will be performed:

- Clinical evaluation;
- Vital signs (BP, Pulse);
- Viral load
- 12-lead ECG;
- Respiratory distress parameters:
  - Respiratory Rate (RR),
  - Oxygen saturation (SpO_2),
  - PaO_2/FiO_2;
- NEWS-2 and/or WHO scales;
- Local blood sample test;
- Concomitant medication;
- Recording of adverse events according to NCI-CTCAE.

For the 7-day evaluation, these assessments can be performed at any daily visit at ICU.

7.10 DAY-14 EVALUATION

The following examinations will be performed:

- Clinical evaluation;
- Vital signs (BP, Pulse);
- Viral load
- 12-lead ECG;

- Respiratory distress parameters:
  - Respiratory Rate (RR),
  - Oxygen saturation (SpO_2),
  - PaO_2/FiO_2;
- NEWS-2 and/or WHO scales;
- Local blood sample test;
- Urinalysis;
- Concomitant medication;
- Recording of adverse events according to NCI-CTCAE.

For the 14-day evaluation, a window of ± 2 days is allowed.

7.11 DAY-20 EVALUATION

The following examinations will be performed:

- Clinical evaluation;
- Vital signs (BP, Pulse);
- 12-lead ECG;
- Respiratory distress parameters:
  - Respiratory Rate (RR),
  - Oxygen saturation (SpO_2),
  - PaO_2/FiO_2;
- NEWS-2 and/or WHO scales;
- Local blood sample test;
- Concomitant medication;
- Recording of adverse events according to NCI-CTCAE (version 5.0) and SAEs.

For the 20-day evaluation, a window of ± 2 days is allowed.

7.12 DAY-40: END OF STUDY VISIT

The following examinations will be performed:

- Clinical evaluation;
- Vital signs (BP, Pulse);
- RT-PCR test;
- Viral load test;
- 12-lead ECG;

- Respiratory distress parameters:
  - Respiratory Rate (RR),
  - Oxygen saturation (SpO_2),
  - PaO_2/FiO_2;
- NEWS-2 and/or WHO scales;
- Urine or serum (ß-hCG) pregnancy test;
- Local blood sample test;
- Blood sample for Anti-glenzocimab antibodies (ADA) assessments will be collected and sent to the central laboratory;
- Concomitant medication;
- Recording of adverse events according to NCI-CTCAE (version 5.0) and SAEs.

For the 40-day evaluation, a window of ± 3 days is allowed.

7.13 ASSESSMENTS AND PROCEDURES

7.13.1 Efficacy Assessment

Efficacy assessments will consist of evaluating respiratory distress parameters, two specific scales and chest CT-scans.

NEWS-2

NEWS2 is the latest version of the National Early Warning Score (NEWS), first produced in 2012 and updated in December 2017, which advocates a system to standardize the assessment of and response to acute illness (*see Appendix 1*).

NEWS is based on a simple aggregate scoring system in which a score is allocated to physiological measurements, already recorded in routine practice, when patients present to, or are being monitored in, hospital. Six simple physiological parameters form the basis of the scoring system:

1. Respiration rate
2. Oxygen saturation
3. Systolic blood pressure
4. Pulse rate
5. Level of consciousness or new confusion*
6. Temperature.

**The patient has new-onset confusion, disorientation and/or agitation, where previously their mental state was normal – this may be subtle. The patient may respond to questions coherently, but there is some confusion, disorientation and/or agitation. This would score 3 or 4 on the GCS (rather than the normal 5 for verbal response), and scores 3 on the NEWS system.*

A score is allocated to each parameter as they are measured, with the magnitude of the score reflecting how extremely the parameter varies from the norm. The score is then aggregated and uplifted by 2 points for people requiring supplemental oxygen to maintain their recommended oxygen saturation.

WHO COVID Ordinal Scoring Scale

Ordinal Scale for Clinical Improvement (see Appendix 2)

CHEST CT-Scan (or in exceptional cases, chest radiogram)

All patients enrolling in the study will undergo a baseline Chest CT-Scan, among other assessments, in order to support the diagnosis and verify complete fulfillment of the inclusion criteria.

Several different patterns should be considered as possibly being related to SARS-CoV-2 infectious pneumonia *(from current published experience)*:

- Ground-glass opacity (≥50%),
- Bilateral patchy shadowing (≥50%),
- Local patchy shadowing (30-50%),
- Interstitial abnormalities (10-25%).

A CT-Scan could be performed at Day 1 (24 hrs) and Day 4 and/or any time thereafter depending on the patient's disease course.

The surface area of lesions should be recorded and compared across all examinations from baseline onwards.

Soluble GPVI

Soluble GPVI (sGPVI) levels will be measured in aliquots of frozen citrated platelet poor plasma (PPP) using a sandwich immunoassay using the MesoScale Discovery (MSD) technology.

The concentrations will be extrapolated from standard curves generated by serial dilutions of recombinant GPVI ectodomain into 5% vol/vol GPVI depleted plasma (see section 7.12.2).

AE and SAE

All AEs experienced during the trial will be recorded in the patient's medical charts and they will be reported in the eCRF in accordance with the AE reporting described in Section 9.6. AEs may be spontaneously reported by the patient or elicited as a result of general questioning by the study staff. Whenever possible, symptoms should be grouped as a single syndrome or diagnosis.

AEs of special interest (AESIs) will comprise the following:

- Bleeding-related events
- Thrombotic events

- Hypersensitivity events

Reference for these events is the last available version of the MeddrA dictionary.

Occurrence and follow-up of AE should be assessed at each visit, and essential data listed hereafter should be recorded in the patient's eCRF and patient's medical file as well:

- the description of the AE;
- the seriousness of the event;
- the onset and end dates of the AE, including the time to onset since the last IMP infusion (if less than 24 hrs);
- the severity or grade of the AE;
- the causality with regards to study drug (glenzocimab or placebo);
- the action(s) taken regarding investigational product;
- the corrective treatment/therapy given;
- the event outcome.

Laboratory tests

All clinical laboratory analyses will be performed at the local laboratory of the investigational site complying with good clinical practice (GCP) and local requirements.
At any time during the study, abnormal laboratory parameters deemed clinically relevant by the investigator (e.g., require delay in administration or discontinuation of study drug, lead to clinical symptoms or signs, or require therapeutic intervention or a change in concomitant treatments), whether specifically requested in the protocol or not, must be recorded as an adverse event on the appropriate section of the eCRF.
Following assessments will be performed at the local laboratory as described in the flowchart:

- Hematology: Hemoglobin, and platelet counts;
- Coagulation: INR, aPTT, PT;
- Biological markers: D-dimers, C Reactive Protein (CRP), Troponin T (TnT), Fibrinogen, IL6, NT proBNP, Pro-calcitonin, Ferritin
- Blood chemistry:
    - Renal function tests: Glomerular Filtfration Rate (GFR), Urea;
    - Other: Serum glucose, LFTs (ASAT, ALAT), LDH.
- Urine or serum (ß-hCG) pregnancy test for women of child bearing potential.

All laboratory tests performed as part of standard of care or for assessment of a possible AE outside of the scheduled visits will be collected as unscheduled assessments.

Vital sign, physical exams and other assessments

The following physical examination, vital signs, cardiac examinations will be performed/measured as described in the flowchart:
Vital signs including heart rate, blood pressure (systolic/diastolic blood pressure, supine), respiratory rate and body temperature (°C);
12-lead ECG. This examination can be repeated in case of occurrence of an adverse event;

The physical examination always comprises the following body systems: cardiovascular, central nervous system, respiratory system.
Clinically significant abnormalities of physical examinations, vital signs, ECG, echocardiogram examinations must be recorded as an adverse event on the appropriate section of the eCRF.
All ECGs as part of standard of care or for assessment of a possible AE outside of the scheduled visits will be collected as unscheduled assessments.

7.13.2 Pharmacokinetics, soluble GPVI and Immunogenicity Assessments

It is of utmost importance to collect all blood samples at the specified times and according to the specifications.
Samples missed or lost, for any reason, should be recorded. Actual date and times of blood collection must be recorded in the eCRF. The date and the time of drug administration should also be precisely recorded.
PK will be assessed and sampling times for blood collection can be found in the study flow chart (see Section 7.1).
Plasma samples for PK, soluble GPVI and immunogenicity assessments will be analyzed by a Central Laboratory and those samples should be transferred according to the detailed procedure described in the Central laboratory instruction manual.

Visit	Time	Sample nr PK	Sample nr ADA	Sample nr sGPVI
Visit 2 Day 2 (V2D2)	Baseline	201	211	221
	3hrs after start of infusion	202		
	3hrs after end of infusion	203		
Visit 3 Day 3 (V3D3)	24hrs after 1st infusion	301		
	3hrs after start of infusion	302		
	3hrs after end of infusion	303		
Visit 4 Day 4 (V4D4)	24hrs after 2nd infusion	401		
	3hrs after start of infusion	402		
	3hrs after end of infusion	403		
Visit 5 Day 5 (V5D5)	24hrs after 3rd infusion	501		
	96hrs evaluation			521
Visit 10 Day 40 (V10D40)	EOS		1011	

Table 1: laboratory assessment and sample numbers

PK analyses will be done at QPS (see Section 9.4.1).

Potential presence of ADA will be determined at QPS in all patients enrolled and sampling times for blood collection can be found in the study flow chart (see Section 7.1). In case the patient presents clinical symptoms suggesting the potential presence of ADA, a blood sample may be collected outside of the scheduled visits as unscheduled ADA assessment see Section 9.4.2).

Sample handling, storage, and shipping conditions will be described in a specific manual intended for the investigating team.
QPS should be provided in due time and with a procedure that complies with blinding conditions with the list of tubes (both for PK and ADA) that stem from patients having received the active IMP.
To avoid any unintended unblinding, all the results related to PK and ADA won't be shared with either the sites staff, the Sponsor nor the CRO staff involved in the study management before the database lock.

8. PRIMARY ENDPOINT

8.1 EFFICACY

8.1.1 Primary Efficacy Endpoint : Progression from moderate to severe respiratory distress assessed at Day 4

The primary efficacy endpoint is a composite failure endpoint defined as the occurrence of at least one of the following failure events at Day 4 :

- RR ≥ 30/min, or/and
- SpO2 decrease > 5% in ambient air. In case where oxygenotherapy cannot be discontinued, please refer to NEWS 2 Scale (item SpO2, scale 2) for adequate conversion (e.g. 93-94 on O2 corresponding to 86-87 in ambient air), PaO_2/FiO_2 ≤ 100mmHg or/and
- Death occurring prior to or on Day 4

9. SECONDARY ENDPOINTS

9.1 EFFICACY

- All cause Death at day 40 and Overall Survival
- WHO-COVID-19 Scale
- NEWS-2 Scale
- Respiratory Rate status defined as::
  - Normal: < 20/min,
  - Mild: 20 ≤ RR < 24/min,
  - Moderate: 24 ≤ RR < 30/min,
  - Severe: ≥ 30/min,
  - Death.
- Hypoxemia status defined as::
  - Normal: > 300 mmHg,
  - Mild: 200 mmHg < PaO2/FiO_2 ≤ 300 mmHg,
  - Moderate: 100 mmHg <PaO2/FIO2 ≤200mmHg,
  - Severe: PaO2/FIO2 ≤ 100mmHg,
  - Death.
- SpO2 status defined as:
  - Normal: > 95%
  - Mild: 93 < SpO2 ≤ 95%,
  - Moderate: 90 < SpO2 ≤ 93%,
  - Severe: ≤ 90%,
  - Death.
- Chest CT-Scan (or in exceptional cases, chest radiogram)
- Oxygen-free days (over the study period = 40 days),
- Need fo mechanical ventilation,

- Mechanical ventilation-free days,
- Hospital-free days (over study period = 40 days),
- Clinical Recovery and Time to Clinical recovery (over study period = 40 days),
- Cure and Time-to-cure (over study period = 40 days).

9.2 SAFETY

- Incidence, nature and severity of Adverse Events, SAEs, SUSARs and Treatment-Emergent Adverse Events (TEAEs),
- Incidence of bleeding-related events,
- Incidence of hypersensitivity reations,
- Changes to vital signs over the course of the study versus screening,
- Change to clinical laboratory assessments (hematology, biochemistry, urinalysis) over the course of the study versus screening,
- ECG over the course of the study versus screening.

9.3 EXPLORATORY VARIABLES (wherever possible)

- C-reactive protein,
- Soluble GPVI (sGPVI),
- D-Dimers,
- Prothrombin time,
- Troponine T,
- IL-6
- Procalcitonin,
- Ferritin
- Lactate dehydrogenase.

9.4 OTHER BIOLOGICAL

9.4.1 Pharmacokinetics (PK)

Glenzocimab plasma concentration will be assayed as described below.
At Days 1, 2 and 3 the following 3 blood samples should be taken:

1. Baseline at the beginning of each treatment period,
2. 3 hrs after the beginning of the first study drug administration,
3. 3 hrs after the end of the study drug administration,

On Day 4, a last sample will take place 24hrs (+/-1hr) after initiation of the last infusion performed at Day 3.

Determination of glenzocimab is done is human platelet-poor plasma. Isolation of glenzocimab is performed by immunocapture, reduction, alkylation, and digestion followed by solid phase

extraction. After evaporation, the reconstituted extracts are analyzed using high performance liquid chromatography-tandem mass spectrometry (LC-MS/MS). The method has been validated at QPS (NL), that will perform the assays for the present study too.
PK results WILL NOT be forwarded to investigators during the course of the study, with exception of an emergency procedure for the management of an SAE if deemed appropriate, this in order to keep the study blind.

9.4.2 Immunogenicity-ADA

Dosing with glenzocimab may lead to the development of glenzocimab-specific antibodies. Presence of anti-glenzocimab antibodies (ADA) will be assessed at the last visit (D20, end of study), as well as in case of immunologic adverse reactions. A validated assay will be used to assess the presence of anti-glenzocimab antibodies in human serum samples. The anti-glenzocimab antibody assay runs on the MSD ECL platform. The samples are incubated with biotinylated glenzocimab (ACT017-Bio) and SULFO-tag labeled ACT017 (ACT017-SULFO). The samples are then transferred to a standard Streptavidin Gold Multi-array 96 well plate. The ACT017-Bio in the formed complex binds to the streptavidin on the plate and detection is performed via the ACT017-SULFO in a Sector Imager S 600 after applying a current to the plate. The intensity of the emitted light is measured.
This method has been validated at QPS (NL) that will performed the assays for the present study too.
Assays should be performed in totality after the end of all patients clinical administration. However, should an SAE be suspected to be of immunologic origin, an emergency assay should be performed immediately upon notification.

9.5 DEFINITIONS

9.5.1 Adverse Event (AE)

An adverse event is any untoward medical occurrence in a patient or clinical trial subject administered a medicinal product and which does not necessarily have a causal relationship with this treatment. An adverse event can therefore be any unfavorable and unintended sign (including an abnormal result of an investigation), symptom or disease.
In clinical studies, an AE can include an undesirable medical condition occurring at any time, including run-in or wash-out periods, even if no experimental treatment has been administered.

9.5.2 Treatment Emergent Adverse Event (TEAE)

Treatment emergent adverse events are defined as adverse events which occur during or within the 30 days after the administration of the first dose of the investigational medicinal product (IMP) or if present before the first administration of IMP, worsens on study treatment.

9.5.3 Adverse Drug Reaction (ADR)

An adverse drug reaction is any untoward and unintended response to a medicinal product related to any dose administered.

9.5.4 Unexpected Adverse Drug Reaction (ADR)

Any adverse drug reaction the nature, severity or outcome of which is not described in the applicable product information.

In this study, the reference document to be used to evaluate the unexpectedness of the adverse drug reactions is section “summary of data and guidance for the investigator” in the Investigator's Brochure (most recent version) for glenzocimab.

9.5.5 Adverse Event of Special Interest (AESI)

Bleeding-related adverse events, thrombotic events, and hypersensitivity events will be subject to a specific analysis.

9.5.6 Laboratory Test Abnormalities

Abnormal laboratory test value should only be reported as an AE if any of the following apply:

- it is accompanied by clinical symptoms or is considered as medically significant by the investigator;
- it results in a change in study treatment schedule of administration (for example, delay in administration, temporary or permanent discontinuation);
- it requires a therapeutic intervention or a change in concomitant treatments.

9.6 REPORTING OF ADVERSE EVENTS

All AEs (related and unrelated to the IMP, expected and unexpected) occurring in the course of the study, from the signature of the study informed consent form and until 30 days after the last dose of the study drug, are to be reported by the investigator.
The Investigator should specify the onset date (with the time to onset since the last IMP infusion (if less than 24 hrs), end date, severity, seriousness, action taken with respect to Investigational Product (glenzocimab or placebo), corrective treatment/therapy given, outcome and his/her opinion as to whether there is a reasonable possibility that the Adverse Event was caused by the (glenzocimab or placebo).
During the study follow-up period (beyond 30 days after the last dose of the study drug) all AEs related to the IMP (glenzocimab or placebo) will be reported.
All AEs reported spontaneously by the patient, elicited as a result of general questioning or observed by the investigator, will be recorded on the AE page(s) of the eCRF. AEs already recorded and described as being "ongoing" must be reviewed at each subsequent evaluation.
For all AEs, the investigator must search and obtain the necessary information to determine the outcome of the AE, whether it corresponds to the definition of a DLT and to assess whether it meets one of the criteria for classification as a serious adverse event (SAE) requiring an immediate notification to the PV Department of AIXIAL.
Any AE considered as related to the IMP (glenzocimab or placebo), will be followed until resolution or stabilization.

9.6.1 Intensity of AE

AEs will be documented and graded according to the NCI-CTCAE version 5.0.
If, for a given AE, the NCI-CTCAE scale is not applicable, the following equivalences will be applied:

- NCI-CTCAE grade 1 corresponds to a mild intensity;
- NCI-CTCAE grade 2 corresponds to a moderate intensity;

- NCI-CTCAE grade 3 corresponds to a severe intensity or a disabling event;
- NCI-CTCAE grade 4 corresponds to a life-threatening event with urgent intervention indicated;
- NCI-CTCAE grade 5 corresponds to death (related to the AE).

9.6.2 Causality

The relationship of an AE to the IMP (glenzocimab or placebo) will be classified according to the following:
Related: an event is considered as related when a causal relationship between the event and the study drug can reasonably be suspected. Reports include good reasons and sufficient information (e.g. plausible time sequence, dose-response relationship, pharmacology) to assume a causal relationship with the study drug in the sense that it is plausible, conceivable or likely.
Not related: an event is considered as not related when a causal relationship between the event and the study drug cannot reasonably be suspected. Reports include good reasons and sufficient information (e.g. implausible time sequence and/or attributable to concomitant disease or other drugs) to rule out a causal relationship with the study drug.

9.7 SERIOUS ADVERSE EVENT

9.7.1 Definitions

Serious Adverse Event (SAE)

Any harmful clinical manifestation that, at any dose, fulfills at least one of the following criteria:

- is fatal (results in death; NOTE : death is an outcome, not an event);
- is life-threatening (NOTE: a "life-threatening event" refers to an event in which the patient was at risk of death at the time of the event; it does not refer to an event which could hypothetically might have caused a death if it were more severe);
- requires hospitalization or prolongation of existing hospitalization;
- results in persistent or significant disability or incapacity*;
- is defined as a congenital anomaly or a birth defect;
- is a medically significant event.

*The terms "disability" and “incapacity” mean any temporary or permanent physical or mental disability clinically significant which affects the physical activity and/or quality of life of the patient.
Is considered medically significant any clinical event or laboratory results considered as serious by the investigator and not corresponding to the seriousness criteria defined above. It may pose a risk to the patient and may require a medical intervention to prevent an outcome corresponding to one of the previously mentioned seriousness criteria (for example, an overdose, a second cancer, pregnancy or any new event which is susceptible to affect the safety of the patients may be considered as medically significant).

In this study, all adverse events of grade 4 will be notified as SAE including laboratory test abnormalities.

Suspected Unexpected Serious Adverse Reaction (SUSAR)

All adverse events, considered as related to the study drug(s) (study drug (glenzocimab or placebo) and which are unexpected and serious (SUSARs) are subject to expedited reporting to the Competent Authorities (CAs) and to the Ethics Committees (EC), in compliance with the local regulatory requirements.
The sponsor or designate will be responsible for reporting the SUSARs, on the basis of the information provided by the investigator.

9.7.2 Reporting

Reporting of SAEs, SUSARs or Serious Safety-related Protocol Deviations and Measures for Prompt Communication

The investigator informs the sponsor of all SAEs (expected and unexpected) whether or not considered as related to the study drug(s) (glenzocimab or placebo) which occur during the study from the signature of the study informed consent and until 30 days after the last dose of the IMP.
The SAE must be reported by the investigator **immediately, or within no more than 24 hrs from the date of the first awareness to the PV department of AIXIAL** (*see Appendix 5*).
The SAE report form must be signed and dated by the principal investigator or by any other investigator designated by the principal investigator as authorized to notify safety issues.
All available information concerning the SAE (anonymous copies of laboratory results, other examinations, hospitalization reports, autopsy report and all other relevant documents) will be transmitted anonymously with the SAE report form.
Further to the notification of a SAE, additional information relative to the SAE may be requested by the sponsor or its representative (by fax, telephone, mail or visit). The investigator must respond to these requests for additional information.
All SAEs must be documented in the patient's medical file, and the SAE report forms (initial report and follow-up reports) must be kept in the investigator's file.
The full requirements of the "ICH Guideline for Clinical Safety Data Management, Definitions and Standards for Expedited Reporting, Topic E2" will be adhered to. The study will be conducted in compliance with all local regulatory requirements.
In compliance with the provisions set forth in the EU guideline issued in July 2017 (EMEA/CHMP/SWP/28367/07 Rev. 1) related to early clinical trials, particular care should be paid to the following:

- All study staff (investigator's, sponsor's, sponsor's representatives and vendors involved in the conduct, monitoring, follow-up or reporting of the present trial should be appropriately trained to the detection, handling, and reporting of the SUSARs, SAEs, and important medical events, with a particular emphasis on those possibly related to a sICH.
- A 24/7 responsible point of contact will have to be in place (with available telephone number) both at sponsor's representative CRO, and at the pharmacovigilance hub (AIXIAL), with for this latter, a direct access to the randomization code, in case of an emergency unblinding (see Section 6.3.3).

- In case a particular related SAEs or the number of defined for triggering an immediate decision on trial continuation need that the inclusions of new patients in the study are immediately suspended, this information should be immediately communicated to the central randomization unit, and to all investigators, participants involved at any site, at least prior to the next dosing. This information should also contain the implementation of corrective or preventative measures to the extent necessary.

The detailed flowchart for processing such information is included in Follow-up of Serious Adverse Events

For any new information concerning an SAE that has already been notified, the investigator must complete a new SAE report form (follow-up report) which will be sent by email **immediately, or within no more than 24 hrs** to the **PV Department of AIXIAL,** accompanied by anonymized copies of the corresponding laboratory results, other examinations and/or hospitalization reports.

The reporting of the SAE follow-up information will be done in the same manner as the initial SAE reports.

Any SAE regardless of relationship to the IMP must be followed until its resolution or stabilization (in case of sequelae). This may lead to the follow-up of the patient beyond the study period.

Serious Adverse Events occurred after the study

No active screening of potential Serious Adverse Events occurring after study discharge (40 days post treatment administration) is expected. However, in case an SAE considered as reasonably related to the study drug(s) *(glenzocimab or placebo))* or to a study procedure would occur after study end, this SAE must be reported to the sponsor or its representative(s) according to the same reporting procedure. It would be forwarded to the competent authorities as for routine pharmacovigilance, analysed and maintained in the product safety database.

Pregnancy

Pregnancy is an exclusion criterion of study but if a patient becomes pregnant within 4 months after the study drug injection, the pregnancy must be reported;

Pregnancies must be reported by the investigator, like SAEs, within 24 hrs of awareness.

The reporting of pregnancy will be done according to the procedure used for the SAEs reporting.

The sponsor or its representative must follow each pregnancy to term.

The pregnancy outcome must be reported by the investigator, to the sponsor or its representative.

10. DATA HANDLING

10.1 CASE REPORT FORM (CRF)

An electronic data capture system will be used for this study. An electronic CRF (eCRF) is designed to record all the data required by the protocol and collected by the Investigator in compliance with Sponsor specifications and requirements regulatory.

Data entry at the investigator's site will be performed by the Investigator or by the designed person from his/her team using the eCRF screens designed with web technologies.

Data entered via the Internet will be directly recorded in the study database.

The Investigator or the designated person from his/her team agrees to complete the eCRF, at each patient visit, and all other documents provided by the Sponsor.

All corrections of data on the eCRF must be made by the Investigator or the designated person from his/her team using electronic data clarifications according to the provided instructions. Any data modification will be recorded in chronological order using the audit trail feature of Oracle inForm database, including date and reason for modification.
In order to ensure confidentiality and security of the data, usernames and passwords will be used to restrict system access to authorized personnel only, whether resident within the investigator's sites, Sponsor or third parties.
The monitor must make certain that all data are completed on the eCRF.
After the last visit of the participant and the lock of the eCRF by the Data Management Department, the Investigator must attest by entering his/her user name and password:

- the authenticity of the data collected in the eCRF;
- the coherence between the data in the eCRF and those in the source documents, with the exception of those data recorded directly in the eCRF and considered as source data.

After comparing the data to the source documents, the monitor will request corrections/clarification from the Investigator using electronic data clarifications using functionalities on line of Source Data Verification.

10.2 DATA MANAGEMENT

SAS® software version 9.3 or later is used for data management process and data are stored in the clinical database stored in a secure folder.
The Data Management Department of the CRO is responsible for data processing including drafting of Data Management Plan, development of Data base structure, drafting the Data Validation Plan (list of edit checks, and SAS Listings), data transfer, SAE reconciliation and data coding: medical history, adverse events and signs and symptoms are coded using the current version of the MedDRA. Prior and concomitant medications are coded using World Health Organization (WHO) drug dictionary (last version available).
When data validation is completed, a review of the data is performed according to the CRO standard operating procedure.
A final database will be declared when all data has been entered, the data validated and the database defined as clean by the Data Manager. After declaration of a final database, the data will be exported from the database to SAS datasets and both the database and the SAS datasets will be frozen and protected from changes. All statistical analyses for the final analysis will be performed on the frozen SAS datasets

11. STATISTICAL METHODS

Complete details of the statistical analyses will be provided in the Statistical Analysis Plan (SAP) which will be finalized prior to the locking and unblinding of the database.

Any modification of the initial statistical method and the reason why they were performed will be described in a specific section of the study statistical analyses plan (SAP) as well as in the clinical study report.

11.1 General considerations

In general, summary tabulations will be presented by treatment arm and will display the number of observations, mean, standard deviation, median, minimum, and maximum for continuous variables, and the number and percent per category for categorical data. The Kaplan-Meier survival curves and 25th, 50th (median), and 75th percentiles will be provided along with their 2-sided 95% confidence intervals (CIs) for time-to-event data.

The nominal one-sided alpha level of significance considered for statistical tests will be 0.025.

As this is essentially an estimation study, no adjustment for multiplicity will be proposed.

Methods for handling missing data will be further detailed in the SAP.

11.2 Randomization and Stratification

Eligible patients will be randomized in a 1:1 allocation ratio either to ACT017 or placebo. Details on the randomization process will be described separately.

11.3 Analysis Sets

- **Safety set:** All enrolled patients having received at least one dose of the prescription of study medication (either ACT017 or placebo). The Safety set will be the basis for safety analyses. Patients will be assigned to the treatment group as treated for the Safety set.

- **Randomized Set (ITT set):** All patients who are randomized into the study and considered as randomized, i.e. with study drug assignments designated according to initial randomization, regardless of whether subjects received what was assigned. Sensitivity efficacy analyses conducted on ITT set could be considered.

- **Full Analysis Set (FAS):** All randomized patients having received at least one dose of the prescription of study drug (either ACT017 or placebo). Patients will be assigned to the treatment group as randomized (i.e. with study drug assignments designated according to initial randomization, regardless of whether subjects received what was assigned). The FAS will be considered as the primary set for efficacy analyses.

- **Per protocol (PP) set:** All FAS patients without any major protocol deviation. Major protocol deviations will be identified during data review meetings that will be held before unblinding and database lock.

11.4 Sample Size Determination

As this is essentially an exploratory study which aim is to estimate the treatment difference in order to better design the future trials, the sample size cannot be justified on a formal power calculation based on a reasonable expected difference.

60 (sixty) patients are considered sufficient to estimate the treatment effect on the efficacy parameters.

As a rough order of magnitude, if the true death rate is assumed to be 0.4 in the placebo group, then a true death rate of 0.10 should be assumed to achieve at least a 80% power to detect this difference in rates at the 1-sided alpha level of 0.025, using an unpooled variance Z test. If the true death rate is assumed to be lower, such as 0.3 in the placebo group, then a true death rate of 0.04 should be assumed to achieve at least a 80% power to detect this difference in rates at the 1-sided alpha level of 0.025, using an unpooled variance Z test.

Assuming a dropout rate of 12%, 68 patients will be enrolled in this study.

With regard to the primary safety endpoint, 60 (2X30) patients randomized will be sufficient to observe in the ACT017 arm at least one serious adverse event which incidence is greater than or equal to 5% with a probability of at least 79%.

11.5 Analyses

11.5.1 Efficacy Analyses

Full details of the analysis of efficacy endpoints will be provided in the SAP.

Primary efficacy endpoint: Progression from moderate to severe respiratory distress assessed at Day 4

Progression from moderate to severe respiratory distress assessed at Day 4 is a composite failure endpoint defined as the occurrence of at least one of the following failure events at Day 4 :

- RR ≥ 30/min, or
- SpO2 decrease > 5% in ambient air. In case where oxygenotherapy cannot be discontinued, please refer to NEWS 2 Scale (item SpO2, scale 2) for adequate conversion (e.g. 93-94 on O2 corresponding to 86-87 in ambient air),
- PaO2/FiO2 ≤ 100mmHg
- Death occurring prior to or on Day 4

The composite event rate will be estimated within each group. The difference in composite event rates and the odds ratio will be estimated along with their asymptotic and exact 95% confidence intervals. p-values from the chi-square and exact Fisher test will also be provided.

Handling missing data:
When the composite endpoint contains missing components, the composite endpoint is a failure if at least one non missing component is a failure. If all non missing components are successes, the composite endpoint is not computable and missing components should be imputed. In that latter case, a procedure for imputing missing components depending on the status of the last available component value (failure or success) will be proposed and detailed in the SAP.

Component-wise secondary analysis: an analysis of each individual binary component will be performed as an aid to the interpretation of the overall result of the composite. The difference in component event rates and the odds ratio will be estimated along with their asymptotic and exact 95% confidence intervals. p-values from the chi-square and exact Fisher test will also be provided. No adjustment for multiplicity will be used.

Multi-component analysis: an additional secondary analysis will be performed to assess the proportion of patients with one failure only, 2 failures and 3 failures among the 3 components (except death).

Sensitivity analyses will be proposed and detailed in the SAP.

Additional analyses of the primary endpoint: the primary composite endpoint will be analyzed in a logistic regression model including treatment effect and relevant covariates (e.g. baseline WHO-Covid-19 scale) that will be specified in the SAP.

Subgroup analyses:
If feasible (i.e. when subgroup sizes are sufficient), subgroup analyses of the primary endpoint will be performed by BMI (<30 vs >=30), Age (< 60 vs >=60) , by baseline WHO-Covid-19 scale , comorbidity, concomitant medications will be provided and further detailed in the SAP.

The same analysis proposed for the primary efficacy endpoint assessed at day 4 will be provided at other time points (Days 1, 2, 3, 7, 14, 20 and 40). The primary endpoint and its components will also be described via plots (e.g., stacked bar plots representing the proportion of subjects in each category (failure or not) , by treatment arm, over time).

Secondary efficacy endpoints

All-Cause Mortality at day 40 (ACM20)

Death rates will be estimated at day 40 within each group.

The difference in ACM20 rates and the odds ratiowill be estimated along with their asymptotic and exact 95% confidence intervals. p-values from the chi-square and exact Fisher test will also be provided.
The All-cause mortality at Day 40 will also be analyzed in a Logistic regression model including treatment, BMI (>30 , <30) , Age (>60 , <60) and baseline respiratory distress status for each primary component (moderate or not). The adjusted odds ratio with its 95% CI for treatment will be estimated in this model.

Subgroup analyses will also be analyzed in the same logistic regression model including treatment, each factor (separately) and a term for treatment-by-factor interaction.

Overall survival

Overall Survival will be defined as the time from the date of randomization to the date of All-cause death.

Patients without documentation of death at the time of analysis (i.e. lost to follow-up) will be censored at the last date they were known to be alive.

Kaplan-Meier (KM) survival curves will be provided by treatment. KM survival medians with their 2-sided 95% Cis will be provided (if estimable) for each treatment group. KM

estimates at Days 1, 2, 3, 4, 14, 20 and 40 will be provided within each group of treatment. A log-rank test will be used to compare treatment arms.

In addition, a Proportional Hazard (PH) Cox model adjusting for treatment will be fitted to estimate the treatment effect expressed in terms of a hazard ratio with the 95% CI and p-value.

Subgroup analyses:
If feasible (i.e. when subgroup sizes are sufficient), analyses of OS will be performed by BMI (<30 vs ≥ 30), Age (< 65 vs ≥ 65) , by baseline WHO-Covid-19 scale, comorbidity, concomitant medications, considering a PH Cox regression model including treatment, each studied factor (considered separately) and a term for treatment-by-factor interaction. The treatment effect (Hazard ratio) by subgroup will be estimated in this model.

WHO COVID-19 Scale

The WHO COVID-19 Scale is 9-point ordinal scale and will not be considered as an interval scale. A categorical analysis will be performed.

A descriptive analysis of each category (count and percentage) will be performed by treatment group at each assessment day. The distribution of outcomes on WHO COVID-19 Ordinal Scoring Scale will also be described via plots (e.g., stacked bar plots representing the proportion of subjects in each category, by treatment arm, over time).

The distribution of the 9-point ordinal scale at each post randomization time point will be compared between groups with a Cochran-Mantel Haenszel test using modified ridit scores (i.e. categorical counterpart of the Wilcoxon test) and stratified by baseline WHO COVID-19 status.

In addition, an analysis will be performed on the change from baseline to each time point which will be transformed into a categorized 3-point ordinal scale as follows: worsened, unchanged, and improved. A descriptive analysis of each category (count and percentage) will be performed by treatment group at each assessment day. The distribution of outcomes will also be described via plots (e.g., stacked bar plots representing the proportion of subjects in each category, by treatment arm, over time).

The analysis of the 3-point ordinal scale will be performed with the same approach proposed for the 9-point ordinal scale. The analysis of the binary endpoint will be performed using a Cochran-Mantel Haenszel test (using modified ridit scores) stratified by baseline WHO COVID-19 status.

Missing data will be replaced by the last available value.

Subgroup analyses:
If feasible (i.e. when subgroup sizes are sufficient), analyses of the WHO COVID-19 scale will be performed by BMI (<30 vs ≥ 30), Age (< 65 vs ≥ 65) , by baseline WHO-Covid-19 scale, comorbidity, concomitant medications with the same approach proposed for the FAS.

NEWS-2 scale

NEWS2 is the latest version of the National Early Warning Score (NEWS) updated in December 2017. As the news-2 scale is not an interval scale, a categorical ordinal analysis will be performed. Thresholds on the aggregated score suggested by the Royal College of Physicians will be used to transform the NEWS-2 scale into a categorized scale (called hereafter the "categorized news-2 scale"). Further details on the thresholds will provided in the SAP.

Missing values of the NEWS-2 scale not due to death will replaced by the last available news-2 scale value before deriving the category of the categorized NEWS-2 scale. Missing values of the NEWS-2 scale due to death occurring prior to or on day D4 will be imputed in the highest category.

A descriptive analysis of each category (count and percentage) will be performed by treatment group at day D4.

The distribution of the categorized news-2 scale at Day D4 will be compared between groups with a Cochran-Mantel Haenszel test using modified ridit scores (i.e. categorical counterpart of the Wilcoxon test) and stratified by baseline categorized news-2 scale values.

The same analysis will be proposed at other time points (Days 1, 2, 3, 14, 20 and 40).

The distribution of outcomes on the categorized news-2 scale will also be described via plots (e.g., stacked bar plots representing the proportion of subjects in each category, by treatment arm, over time).

Other secondary endpoints

The analysis of the other secondary endpoints will be provided in the SAP.

11.5.2 Safety Analyses

Usual descriptive statistics will be used and detailed in the SAP to analyze the safety parameters as follows:

- Deaths, SAEs, TEAEs and bleeding events, and of patients withdrawn because of safety concerns.
- Type, time of occurrence post dosing, frequency, seriousness, severity and relatedness of IMP emergent adverse events (TEAEs) will be analyzed. TEAEs will be analyzed according to current version of the MedDRA (Medical Dictionary for Regulatory Affairs) dictionary.
- Vital signs will be described and analyzed as needed at the various time-points across the whole study period.
- Laboratory abnormalities will be described and listed by dose level and for the placebo. Reference will be made to multiples of the upper normal limit (UNL) as defined by the laboratory's specifications. Shift tables will be issued.
- Immunogenicity will be analyzed both in frequency (including the presence of non-clinically relevant ADA) and severity if clinically expressed, and per dose-group level.

11.5.3 Pharmacokinetic Endpoints Analysis

Pharmacokinetics will be described using all time-points and will be represented graphically.

12. QUALITY CONTROL/ MONITORING

Monitoring is the act of overseeing the progress of a clinical trial, and of ensuring that it is conducted, recorded, and reported in accordance with the protocol, Standard Operating Procedures (SOPs), Good Clinical Practice (GCP), and the applicable regulatory requirement(s).
The main responsibilities of the CRA are to verify investigators' adherence to the protocol and that informed consent is obtained and recorded for all patients prior to implementation of any study procedure.
The CRA will contact and visit the investigator at regular intervals during the study. He will compare the CRFs with medical records and other relevant documentation through direct access, during the on-site monitoring visits. He ensures the completeness, consistency and accuracy of the data being recorded in the CRF by the investigator. Monitoring activities will be adapted according to local guidelines provided by competent authorities during the COVID-19 pandemic period.
The CRA will explain the protocol and study related procedures to all study staff, including the investigator. If new collaborators are included during the course of the study, additional training sessions will be organised by the investigator and/or the CRA.
As part of the supervision of the study progress other sponsor personnel or the CRO may, on request, accompany the CRA on visits to the study site. The investigator and his collaborators commit to cooperate with the CRA to resolve any problems, corrections, or possible misunderstandings concerning the findings detected in the course of these monitoring visits.

13. RESPONSIBILITIES

13.1 SPONSOR'S RESPONSIBILITIES

The sponsor or its representative will submit an application to Ethics Committees//Institutional Review Boards (whether central or local) and Competent Authorities for approval of the clinical study. A copy of the Ethics Committee's/IRB's and Competent Authorities' approvals must be received by the sponsor before the study starts.
In accordance with the provisions of the law and the GCP, the sponsor will have an insurance policy intended to guarantee against possible damage resulting from the research.
The studies and/or experiments performed on behalf of the sponsor will be specifically and expressly guaranteed. It is advisable to underline that noncompliance with the Research Legal Conditions is a cause for guarantee exclusion.

13.2 INVESTIGATOR'S RESPONSIBILITIES

The investigator is responsible for ensuring the accuracy, completeness, legibility, and timeliness of the data reported. All source documents should be completed in a neat, legible manner and kept up-to-date to ensure accurate interpretation of data.
The investigator will maintain adequate and accurate records in accordance with ICH Good Clinical Practice to enable the conduct of the study to be fully documented and the study data to be subsequently verified for trial-related monitoring, audits, IRB/IEC review, and regulatory inspection.
Agreement of the investigator to conduct and administer this study in accordance with the protocol will be documented in separate study agreements.
The investigator will be responsible for giving information and training about the study to all staff members involved in the study or in any element of subject management, both before

starting the practical performance of the study and during the course of the study (e.g., when new staff become involved).
The investigator will maintain a record of all individuals involved in the study (medical, nursing and other staff) and will specify, for each person, the tasks delegated for the study. The investigator is responsible for ensuring the privacy, health, and welfare of the patients during and after the study.
The investigator must be familiar with the background and requirements of the study and with the properties of the investigational product as described in the SmPC/USPI/IB/Product Labeling Information as well as the synthetic data for the use of IMP.

14. CONFIDENTIALITY AND ARCHIVING

14.1 PERSONAL DATA PROTECTION AND CONFIDENTIALITY

The Sponsor (or the delegated representative) is the data controller of the study and the protocol will be conducted in accordance with the European Regulation No. 2016/679 of 27 April 2016 (General Data Protection Regulation) and national law.
The patients will be informed by the information and consent form that their personal data collected for the purpose of the study will be kept confidential and will be processed by the Sponsor and its representatives. The patients will also be informed of their rights according to General Data Protection Regulation.
The investigator must assure that the personal data of patients, including their identity and all other personal medical information, will be kept confidential at any time.
Patient number and initials will identify the patients in the CRF. On other documents or photographic materials (including the results of imaging) submitted to the sponsor, patients will not be identified by their names but by an identification code (e.g. patient number).
By signing this protocol, the investigator undertakes that the protocol and all attached information are and will remain confidential. The investigator agrees that after providing the protocol and all information necessary for the personnel involved, he remains responsible for their total confidentiality. Such obligation is detailed in the confidentiality agreement signed by the investigator before the initiation of the study.
The investigator agrees that, subject to local regulations and ethical considerations, a sponsor representative or any regulatory agency may consult directly and/or copy study documents in order to verify a case report, provided that the subject's identity remains anonymous.
The investigator undertakes to treat all subjects data used or disclosed in connection with the conduct of study in compliance with European and local applicable laws relating to data protection.
The investigator will be responsible for keeping a list of all enrolled patients including patient numbers, full names and date of birth.

14.2 STUDY DOCUMENTATION & ARCHIVING

The investigator must maintain adequate and accurate records to enable the conduct of the study to be fully documented and the study data to be subsequently verified. The study documents, including patient CRFs, should be classified in the investigator's file.
The investigator's file will contain the protocol/amendments, independent ethics committee and health authorities with correspondence, sample informed consent, IMP records, staff curriculum vitae and authorization forms, correspondence, etc.
The investigator must keep the Study File until the Sponsor authorization of destruction and by default during at least 15 years after completion or discontinuation of the study.

Should the investigator wish to assign the study records to another party or move them to another location, the sponsor must be notified in advance.

15. ETHICAL AND REGULATORY ASPECTS

15.1 PATIENT'S INFORMATION NOTE AND WRITTEN CONSENT FORM

The information note must be given to the patients before their decision to participate or abstain from participation, according to local requirements and Good Clinical Practice. This information is based on the elements set out in the Declaration of Helsinki and the ICH-GCP Guidelines. It must also describe the measures taken to safeguard patient's privacy and protection of personal data, according to European RGPD. Restraints and risks must be explained, as well as the right to discontinue participation in the study at any stage, without affecting their further relationship with the investigator and/or their future care.
The information note and written consent form must be submitted by the investigator to the patient with an oral explanation. The consent must be agreed and signed by the patient or legal representant according to local regulations before any study-related procedure starts.
Moreover, regarding the population to be included emergency consent procedure will be implemented in the study. The procedure will be adapted in each country according to local regulation on emergency consent and inclusion procedures. Consent confirmation will be systematically done when patient become able of consent by themselves in accordance to local regulation requirement.
The consent form is made in duplicate: the original copy is kept by the Investigator and one copy is given to the patient.
If any information becomes available during the trial that may be relevant to the patient's willingness to keep on participating in the trial, an updated written informed consent must be submitted to the patient to confirm his agreement to continue participating.

15.2 ACCESS TO SOURCE DATA

The investigator must authorize the Clinical Research Associate (CRA) to have direct access to all source documents concerning the patient necessary for the verification of data listed in the CRF.
At the time of the visit of the CRA, the investigator must provide the CRA with printouts of electronic medical files, signed by the investigator. The CRA must date and sign all pages of each printout after comparing them to the CRF.
In case of requests from the Health Authorities, The Investigator will permit direct access to all study documents, IP accountability records, medical records and source data.
According to ICH-GCP requirements, the patient will be informed in writing about the need for Source Data Verification (SDV) for quality control, and audits/inspections.

15.3 STUDY CONDUCT

The study will be conducted according to the ICH Good Clinical Practices standards, Declaration of Helsinki, as well as any European and/or local applicable laws and regulations relating to the conduct of the study.

15.4 ETHICS COMMITTEES AND COMPETENT AUTHORITIES

This protocol and other documents (e.g. Investigator's Brochure, Patient Information, etc.) will be submitted by the sponsor/CRO to an IEC/IRB. Before starting the study, the investigator

must have received written approval of the Study Protocol and the Patient Information Consent Form from the IEC/IRB. The IEC/IRB approval must reveal the Study Protocol version as well as the documents reviewed.
During the study, the sponsor must inform the IEC of any changes to the protocol, all SUSARs and any new information that may affect the safety of the patients or the conduct of the study.
As required by local regulations, the sponsor and the investigators, are required to verify that all legal aspects are respected and that the approval of the relevant regulatory authorities where appropriate has been obtained, prior to study initiation.
The Summary of Product Characteristics and/or the Investigator's Brochure/The operating instructions, the Study Protocol, the name and site of the investigators, the opinion of the IEC(s)/IRBs will be submitted to the National Authorities before the beginning of the study.
The relevant local authorities have to be informed by the sponsor about the clinical study implementation and its status.

15.5 AMENDMENTS

Neither the investigator nor the sponsor may alter the protocol without the authorization of the other party. Any amendment that may be issued, must be dated and signed by both parties and must appear as an amendment to the protocol before implementation.
Substantial amendments are submitted for opinion/approval/authorization to IEC/IRB and CA before implementation.
Substantial amendments on safety measures are submitted for approval/authorization to EC and CA but could be implemented immediately under specific conditions defined with the sponsor.

15.6 AUDIT AND INSPECTION

The investigator must accept that the source documents for this trial should be made available to auditors (qualified personnel from the sponsor or its designees) or to Health Authority inspectors after appropriate notification. The CRF data verification must be carried out by direct control of source documents. The investigator will be available to answer any question asked by the auditors/inspectors and will make every effort to facilitate the proper conduct of the audit/inspection.
In case of inspection, the investigator will inform the sponsor who should participate to the inspection.

16. PUBLICATION OF DATA

The data and research results are the exclusive property of the sponsor. No information or publication may be made without prior authorization of the sponsor.
The sponsor reserves the right to publish or use them in any form whatsoever, to submit them to Health Authorities of any country, on behalf or on behalf of its subsidiaries.
Should the study generate results likely to be patented, then only the sponsor will be authorized to file such a patent, in his name and at his expenses.
The final clinical study report will be written according to the ICH E3 Guideline (Structure and Content of Clinical Study Reports) and according to all applicable regulations.

17. BIBLIOGRAPHY

1) Coomes E, Haghbayan H. Interleukin-6 in COVID-19: A Systematic Review and Meta-Analysis. *medRxiv* **2020**.

2) Channappanavar R, Perlman S. Pathogenic human coronavirus infections: causes and consequences of cytokine storm and immunopathology. *Semin Immunopathol* **2017**; 39:529-39.

3) Jin Y, Yang H, Ji W, et al. Virology, Epidemiology, Pathogenesis, and Control of COVID-19. *Viruses* **2020**; 12.

4) Herold T, Jurinovic V, Arnreich C, et al. Level of IL-6 predicts respiratory failure in hospitalized symptomatic COVID-19 patients. *medRxiv* **2020**.

5) Huang C, Wang Y, Li X, et al. Clinical features of patients infected with 2019 novel coronavirus in Wuhan, China. *Lancet* **2020**; 395:497-506.

6) Kemps B, Hauffman C. *COVID REFERENCE* 2020.03, Sprinhauser Verlag, downloaded 29.4.2020

7) Lescure F-X, Boudama L, Nguyen D et al. Clinical and virolgical data of the first cases of Covid-19 in Europe: a case series. Lancet, published online March 27, 2020. doi.org/10.1016/S1473-3099(20)30200-0

8) Luo P, Liu Y, Qiu L, Liu X, Liu D, Li J. Tocilizumab treatment in COVID-19: a single center experience. *J Med Virol* **2020**

9) McGonagel D, O'Donnell JS, Sharif K et al. Immune mechanisms of pulomonary coagulopathy in Covid-19 pneumonia. *Lancet Rhumatol* 2020. doi.org/10.1016/S2665-9913.

10) McGonagel D, Sharif K, O'Regan A et al. The role of cytokines including interleukin-6 in Covid 19 induced pneumonia and macro[hage activating syndrome-loke disease. Autoimmunity Reviews 2020. doi.org/10.1016/j.autrev.2020.102537.

11) Michot JM, Albiges L, Chaput N, et al. Tocilizumab, an anti-IL6 receptor antibody, to treat Covid-19-related respiratory failure: a case report. *Ann Oncol* **2020**.

12) Richardson S, Hirsch J, Narasimhan M, et al. Presenting Characteristics, Comorbidities, and Outcomes Among 5700 Patients Hospitalized With COVID-19 in the New York City Area. *JAMA* April 22, 2020.

13) Toniati P, Piva S, Cattalini M, et al. Tocilizumab for the treatment of severe COVID-19 pneumonia with hyperinflammatory syndrome and acute respiratory failure: A single center study of 100 patients in Brescia, Italy. *Autoimmun Rev* **2020**:102568.

14) Wu Z, McGoogan JM. Characteristics of and Important Lessons From the Coronavirus Disease 2019 (COVID-19) Outbreak in China: Summary of a Report of 72314 Cases From the Chinese Center for Disease Control and Prevention. *JAMA* **2020**.

18. APPENDICES

Appendix 1: NEWS-2 Scale

National Early Warning Score (NEWS)*

PHYSIOLOGICAL PARAMETERS	3	2	1	0	1	2	3
Respiration Rate	≤8		9 - 11	12 - 20		21 - 24	≥25
Oxygen Saturations	≤91	92 - 93	94 - 95	≥96			
Any Supplemental Oxygen		Yes		No			
Temperature	≤35.0		35.1 - 36.0	36.1 - 38.0	38.1 - 39.0	≥39.1	
Systolic BP	≤90	91 - 100	101 - 110	111 - 219			≥220
Heart Rate	≤40		41 - 50	51 - 90	91 - 110	111 - 130	≥131
Level of Consciousness				A			V, P, or U

Appendix 2: WHO COVID-19 Ordinal Scoring Scale

Patient State	Descriptor	Score
Uninfected	No clinical or virological evidence of infection	0
Ambulatory	No limitation of activities	1
	Limitation of activities	2
Hospitalized Mild disease	Hospitalized, no oxygen therapy	3
	Oxygen by mask or nasal prongs	4
Hospitalized Severe Disease	Non-invasive ventilation or high-flow oxygen	5
	Intubation and mechanical ventilation	6
	Ventilation + additional organ support – pressors, RRT, ECMO	7
Dead	Death	8

Appendix 3 : Conversion tables for estimation of PaO_2 and FiO_2

MAY 8, 2007 EPIC II

Instructions to complete the CRFs

Conversion tables

1 Estimating PaO_2 from a given SO_2

SO_2 (%)	PaO_2 (mmHg)
80	44
81	45
82	46
83	47
84	49
85	50
86	52
87	53
88	55
89	57
90	60
91	62
92	65
93	69
94	73
95	79
96	86
97	96
98	112
99	145

2 Estimating FiO_2

Method	O_2 flow (l/min)	Estimated FiO2 (%)
Nasel cannula	1	24
	2	28
	3	32
	4	36
	5	40
	6	44
Nasopharyngeal catheter	4	40
	5	50
	6	60
Face mask	5	40
	6-7	50
	7-8	60
Face mask with reservoir	6	60
	7	70
	8	80
	9	90
	10	95

Appendix 4: Helsinki Declaration

WORLD MEDICAL ASSOCIATION - DECLARATION OF HELSINKI
Ethical Principles for Medical Research Involving Human Subjects

Adopted by the 18th WMA General Assembly, Helsinki, Finland, June 1964
and amended by the:
29th WMA General Assembly, Tokyo, Japan, October 1975
35th WMA General Assembly, Venice, Italy, October 1983
41st WMA General Assembly, Hong Kong, September 1989
48th WMA General Assembly, Somerset West, Republic of South Africa, October 1996
52nd WMA General Assembly, Edinburgh, Scotland, October 2000
53rd WMA General Assembly, Washington DC, USA, October 2002 (Note of Clarification added)
55th WMA General Assembly, Tokyo, Japan, October 2004 (Note of Clarification added)
59th WMA General Assembly, Seoul, Republic of Korea, October 2008
64th WMA General Assembly, Fortaleza, Brazil, October 2013

Preamble

1. The World Medical Association (WMA) has developed the Declaration of Helsinki as a statement of ethical principles for medical research involving human subjects, including research on identifiable human material and data.

The Declaration is intended to be read as a whole and each of its constituent paragraphs should be applied with consideration of all other relevant paragraphs.

2. Consistent with the mandate of the WMA, the Declaration is addressed primarily to physicians. The WMA encourages others who are involved in medical research involving human subjects to adopt these principles.

General Principles

3. The Declaration of Geneva of the WMA binds the physician with the words, "The health of my patient will be my first consideration," and the International Code of Medical Ethics declares that, "A physician shall act in the patient's best interest when providing medical care."

4. It is the duty of the physician to promote and safeguard the health, well-being and rights of patients, including those who are involved in medical research. The physician's knowledge and conscience are dedicated to the fulfilment of this duty.

5. Medical progress is based on research that ultimately must include studies involving human subjects.

6. The primary purpose of medical research involving human subjects is to understand the causes, development and effects of diseases and improve preventive, diagnostic and therapeutic interventions (methods, procedures and treatments). Even the best proven interventions must be evaluated continually through research for their safety, effectiveness, efficiency, accessibility and quality.

7. Medical research is subject to ethical standards that promote and ensure respect for all human subjects and protect their health and rights.

8. While the primary purpose of medical research is to generate new knowledge, this goal can never take precedence over the rights and interests of individual research subjects.

9. It is the duty of physicians who are involved in medical research to protect the life, health, dignity, integrity, right to self-determination, privacy, and confidentiality of personal information of research subjects. The responsibility for the protection of research subjects must always rest with the physician or other health care professionals and never with the research subjects, even though they have given consent.

10. Physicians must consider the ethical, legal and regulatory norms and standards for research involving human subjects in their own countries as well as applicable international norms and standards. No national or international ethical, legal or regulatory requirement should reduce or eliminate any of the protections for research subjects set forth in this Declaration.

11. Medical research should be conducted in a manner that minimises possible harm to the environment.

12. Medical research involving human subjects must be conducted only by individuals with the appropriate ethics and scientific education, training and qualifications. Research on patients or healthy volunteers requires the supervision of a competent and appropriately qualified physician or other health care professional.

13. Groups that are underrepresented in medical research should be provided appropriate access to participation in research.

14. Physicians who combine medical research with medical care should involve their patients in research only to the extent that this is justified by its potential preventive, diagnostic or therapeutic value and if the physician has good reason to believe that participation in the research study will not adversely affect the health of the patients who serve as research subjects.

15. Appropriate compensation and treatment for subjects who are harmed as a result of participating in research must be ensured.

Risks, Burdens and Benefits

16. In medical practice and in medical research, most interventions involve risks and burdens. Medical research involving human subjects may only be conducted if the importance of the objective outweighs the risks and burdens to the research subjects.

17. All medical research involving human subjects must be preceded by careful assessment of predictable risks and burdens to the individuals and groups involved in the research in comparison with foreseeable benefits to them and to other individuals or groups affected by the condition under investigation.

Measures to minimise the risks must be implemented. The risks must be continuously monitored, assessed and documented by the researcher.

18. Physicians may not be involved in a research study involving human subjects unless they are confident that the risks have been adequately assessed and can be satisfactorily managed.

When the risks are found to outweigh the potential benefits or when there is conclusive proof of definitive outcomes, physicians must assess whether to continue, modify or immediately stop the study.

Vulnerable Groups and Individuals

19. Some groups and individuals are particularly vulnerable and may have an increased likelihood of being wronged or of incurring additional harm.

All vulnerable groups and individuals should receive specifically considered protection.

20. Medical research with a vulnerable group is only justified if the research is responsive to the health needs or priorities of this group and the research cannot be carried out in a non-vulnerable group. In addition, this group should stand to benefit from the knowledge, practices or interventions that result from the research.

Scientific Requirements and Research Protocols

21. Medical research involving human subjects must conform to generally accepted scientific principles, be based on a thorough knowledge of the scientific literature, other relevant sources of information, and adequate laboratory and, as appropriate, animal experimentation. The welfare of animals used for research must be respected.

22. The design and performance of each research study involving human subjects must be clearly described and justified in a research protocol.

The protocol should contain a statement of the ethical considerations involved and should indicate how the principles in this Declaration have been addressed. The protocol should include information regarding funding, sponsors, institutional affiliations, potential conflicts of interest, incentives for subjects and information regarding provisions for treating and/or compensating subjects who are harmed as a consequence of participation in the research study.

In clinical trials, the protocol must also describe appropriate arrangements for post-trial provisions.

Research Ethics Committees

23. The research protocol must be submitted for consideration, comment, guidance and approval to the concerned research ethics committee before the study begins. This committee must be transparent in its functioning, must be independent of the researcher, the sponsor and any other undue influence and must be duly qualified. It must take into consideration the laws and regulations of the country or countries in which the research is to be performed as well as applicable international norms and standards but these must not be allowed to reduce or eliminate any of the protections for research subjects set forth in this Declaration.

The committee must have the right to monitor ongoing studies. The researcher must provide monitoring information to the committee, especially information about any serious adverse

events. No amendment to the protocol may be made without consideration and approval by the committee. After the end of the study, the researchers must submit a final report to the committee containing a summary of the study's findings and conclusions.

Privacy and Confidentiality

24. Every precaution must be taken to protect the privacy of research subjects and the confidentiality of their personal information.

Informed Consent

25. Participation by individuals capable of giving informed consent as subjects in medical research must be voluntary. Although it may be appropriate to consult family members or community leaders, no individual capable of giving informed consent may be enrolled in a research study unless he or she freely agrees.

26. In medical research involving human subjects capable of giving informed consent, each potential subject must be adequately informed of the aims, methods, sources of funding, any possible conflicts of interest, institutional affiliations of the researcher, the anticipated benefits and potential risks of the study and the discomfort it may entail, post-study provisions and any other relevant aspects of the study. The potential subject must be informed of the right to refuse to participate in the study or to withdraw consent to participate at any time without reprisal. Special attention should be given to the specific information needs of individual potential subjects as well as to the methods used to deliver the information.

After ensuring that the potential subject has understood the information, the physician or another appropriately qualified individual must then seek the potential subject's freely-given informed consent, preferably in writing. If the consent cannot be expressed in writing, the non-written consent must be formally documented and witnessed.

All medical research subjects should be given the option of being informed about the general outcome and results of the study.

27. When seeking informed consent for participation in a research study the physician must be particularly cautious if the potential subject is in a dependent relationship with the physician or may consent under duress. In such situations the informed consent must be sought by an appropriately qualified individual who is completely independent of this relationship.

28. For a potential research subject who is incapable of giving informed consent, the physician must seek informed consent from the legally authorised representative. These individuals must not be included in a research study that has no likelihood of benefit for them unless it is intended to promote the health of the group represented by the potential subject, the research cannot instead be performed with persons capable of providing informed consent, and the research entails only minimal risk and minimal burden.

29. When a potential research subject who is deemed incapable of giving informed consent is able to give assent to decisions about participation in research, the physician must seek that assent in addition to the consent of the legally authorised representative. The potential subject's dissent should be respected.

30. Research involving subjects who are physically or mentally incapable of giving consent, for example, unconscious patients, may be done only if the physical or mental condition that prevents giving informed consent is a necessary characteristic of the research group. In such circumstances the physician must seek informed consent from the legally authorised representative. If no such representative is available and if the research cannot be delayed, the study may proceed without informed consent provided that the specific reasons for involving subjects with a condition that renders them unable to give informed consent have been stated in the research protocol and the study has been approved by a research ethics committee. Consent to remain in the research must be obtained as soon as possible from the subject or a legally authorised representative.

31. The physician must fully inform the patient which aspects of their care are related to the research. The refusal of a patient to participate in a study or the patient's decision to withdraw from the study must never adversely affect the patient-physician relationship.

32. For medical research using identifiable human material or data, such as research on material or data contained in biobanks or similar repositories, physicians must seek informed consent for its collection, storage and/or reuse. There may be exceptional situations where consent would be impossible or impracticable to obtain for such research. In such situations the research may be done only after consideration and approval of a research ethics committee.

Use of Placebo

33. The benefits, risks, burdens and effectiveness of a new intervention must be tested against those of the best proven intervention(s), except in the following circumstances:

Where no proven intervention exists, the use of placebo, or no intervention, is acceptable; or

Where for compelling and scientifically sound methodological reasons the use of any intervention less effective than the best proven one, the use of placebo, or no intervention is necessary to determine the efficacy or safety of an intervention and the patients who receive any intervention less effective than the best proven one, placebo, or no intervention will not be subject to additional risks of serious or irreversible harm as a result of not receiving the best proven intervention.

Extreme care must be taken to avoid abuse of this option.

Post-Trial Provisions

34. In advance of a clinical trial, sponsors, researchers and host country governments should make provisions for post-trial access for all participants who still need an intervention identified as beneficial in the trial. This information must also be disclosed to participants during the informed consent process.

Research Registration and Publication and Dissemination of Results

35. Every research study involving human subjects must be registered in a publicly accessible database before recruitment of the first subject.

36. Researchers, authors, sponsors, editors and publishers all have ethical obligations with regard to the publication and dissemination of the results of research. Researchers have a duty to make publicly available the results of their research on human subjects and are accountable for the completeness and accuracy of their reports. All parties should adhere to accepted guidelines for ethical reporting. Negative and inconclusive as well as positive results must be published or otherwise made publicly available. Sources of funding, institutional affiliations and conflicts of interest must be declared in the publication. Reports of research not in accordance with the principles of this Declaration should not be accepted for publication.

Unproven Interventions in Clinical Practice

37. In the treatment of an individual patient, where proven interventions do not exist or other known interventions have been ineffective, the physician, after seeking expert advice, with informed consent from the patient or a legally authorised representative, may use an unproven intervention if in the physician's judgement it offers hope of saving life, re-establishing health or alleviating suffering. This intervention should subsequently be made the object of research, designed to evaluate its safety and efficacy. In all cases, new information must be recorded and, where appropriate, made publicly available.

Appendix 5: Steps for Handling ACT-CS-006 SAEs

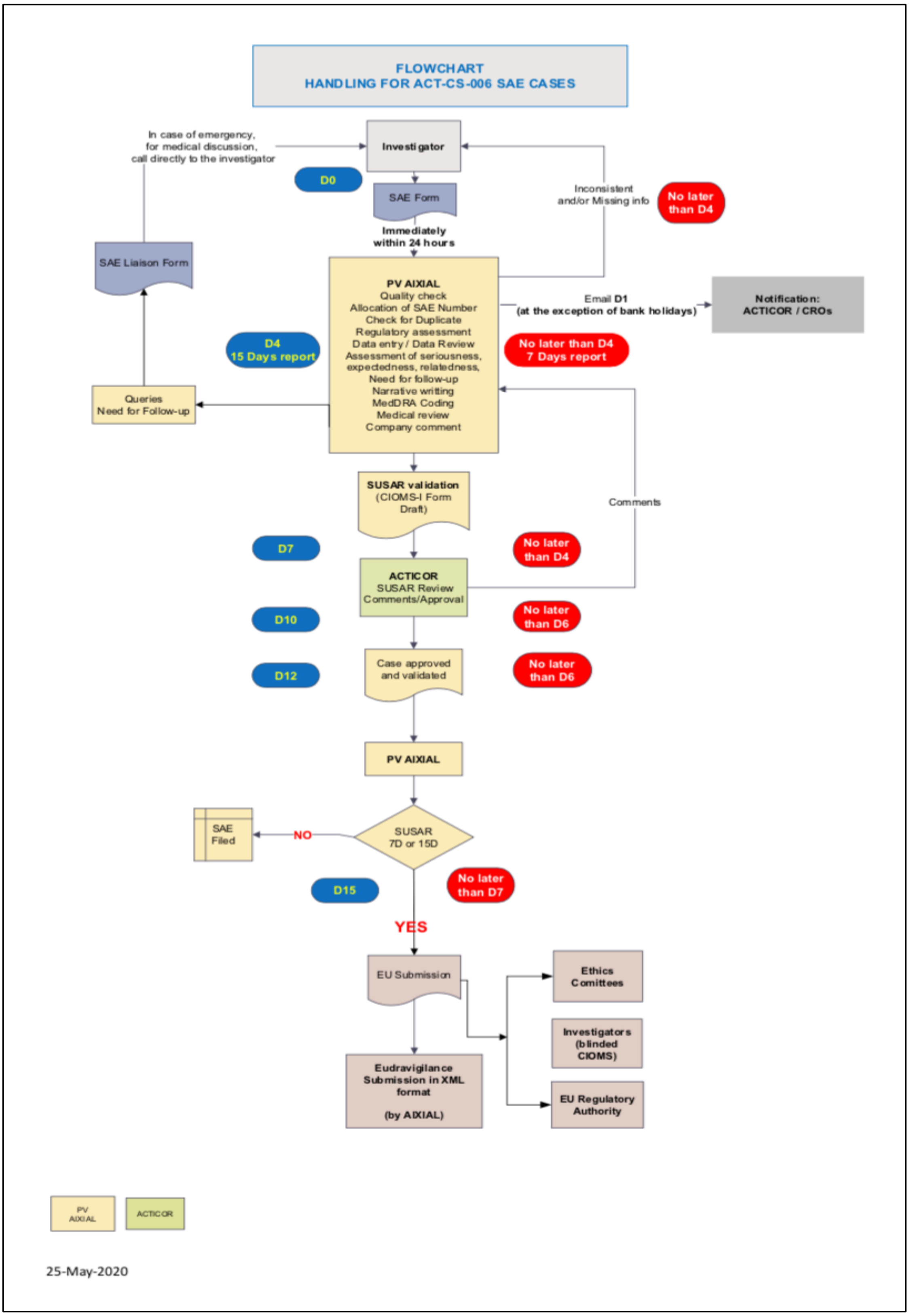

Supplement: S2 Appendix — (PDF) [file pone.0302897.s007.pdf]
